# A systematic review of substance use and substance use disorder research in Kenya

**DOI:** 10.1371/journal.pone.0269340

**Published:** 2022-06-09

**Authors:** Florence Jaguga, Sarah Kanana Kiburi, Eunice Temet, Julius Barasa, Serah Karanja, Lizz Kinyua, Edith Kamaru Kwobah

**Affiliations:** 1 Department of Mental Health, Moi Teaching & Referral Hospital, Eldoret, Kenya; 2 Department of Mental Health, Mbagathi Hospital, Nairobi, Kenya; 3 Department of Mental Health & Behavioral Sciences, Moi University School of Medicine, Eldoret, Kenya; 4 Population Health, Academic Model Providing Access to Healthcare, Eldoret, Kenya; 5 Department of Mental Health, Gilgil Sub-County Hospital, Gilgil, Kenya; 6 Intensive Care Unit, Aga Khan University Hospital, Nairobi, Kenya; University of Washington, UNITED STATES

## Abstract

**Objectives:**

The burden of substance use in Kenya is significant. The objective of this study was to systematically summarize existing literature on substance use in Kenya, identify research gaps, and provide directions for future research.

**Methods:**

This systematic review was conducted in line with the PRISMA guidelines. We conducted a search of 5 bibliographic databases (PubMed, PsychINFO, Web of Science, Cumulative Index of Nursing and Allied Professionals (CINAHL) and Cochrane Library) from inception until 20 August 2020. In addition, we searched all the volumes of the official journal of the National Authority for the Campaign Against Alcohol & Drug Abuse (the African Journal of Alcohol and Drug Abuse). The results of eligible studies have been summarized descriptively and organized by three broad categories including: studies evaluating the epidemiology of substance use, studies evaluating interventions and programs, and qualitative studies exploring various themes on substance use other than interventions. The quality of the included studies was assessed with the Quality Assessment Tool for Studies with Diverse Designs.

**Results:**

Of the 185 studies that were eligible for inclusion, 144 investigated the epidemiology of substance use, 23 qualitatively explored various substance use related themes, and 18 evaluated substance use interventions and programs. Key evidence gaps emerged. Few studies had explored the epidemiology of hallucinogen, prescription medication, ecstasy, injecting drug use, and emerging substance use. Vulnerable populations such as pregnant women, and persons with physical disability had been under-represented within the epidemiological and qualitative work. No intervention study had been conducted among children and adolescents. Most interventions had focused on alcohol to the exclusion of other prevalent substances such as tobacco and cannabis. Little had been done to evaluate digital and population-level interventions.

**Conclusion:**

The results of this systematic review provide important directions for future substance use research in Kenya.

**Systematic review registration:**

PROSPERO: CRD42020203717.

## Introduction

Globally, substance use is associated with significant morbidity and mortality. In the 2017 Global Burden of Disease (GBD) study, substance use disorders (SUDs) were the second leading cause of disability among the mental disorders with 31,052,000 (25%) Years Lived with Disability (YLD) attributed to them [1]. In 2016, harmful alcohol use resulted in 3 million deaths (5.3% of all deaths) worldwide and 132.6 (5.1%) million disability-adjusted life years (DALYs) [2]. Tobacco use, the leading cause of preventable death, kills more than 8 million people worldwide annually [3]. Alcohol and tobacco use are leading risk factors for non-communicable diseases for example cardiovascular disease, cancer, and liver disease [3, 4]. Even though the prevalence rate of opioid use is small compared to that of tobacco and alcohol use, opioid use disorder contributes to 76% of all deaths from SUDs [4]. Other psychoactive substances such as cannabis and amphetamines are associated with mental health consequences including increased risk of suicidality, depression, anxiety and psychosis [5, 6]. In addition to the effect on health, substance use is associated with significant socio-economic costs arising from its impact on health and criminal justice systems [7].

Low- and middle-income countries (LMICs) bear the burden of substance use. Over 80% of the 1.3 billion tobacco users worldwide live in LMICs [3]. In 2016, the alcohol-attributable disease burden was highest in LMICs compared to upper-middle-income and high-income countries (HICs) [2]. In Kenya, a nationwide survey conducted in 2017 reported that over 10% of Kenyans between the ages of 15 to 65 years had a SUD [8]. In another survey, 20% of primary school children had ever used at least one substance in their lifetime [9]. Moreover, Kenya has the third highest total DALYs (54,000) from alcohol use disorders (AUD) in Africa [4] Unfortunately, empirical work on substance use in LMICs is limited [10, 11]. In a global mapping of SUD research, majority of the work had been conducted in upper-middle income and HICs (HICs) [11]. In a study whose aim was to document the existing work on mental health in Botswana, only 7 studies had focused on substance use [10]. Information upon which policy and interventions could be developed is therefore lacking in low-and-middle income settings.

Since the early 1980s, scholars in Kenya began engaging in research to document the burden and patterns of substance use [12]. In 2001 the National Authority for the Campaign Against Alcohol and Drug Abuse (NACADA) was established in response to the rising cases of harmful substance use in the country particularly among the youth. The mandate of the Authority was to educate the public on the harms associated with substance use [13]. In addition to prevention work, NACADA contributes to research by conducting general population prevalence surveys every 5 years and recently launched its journal, the African Journal of Alcohol and Drug Abuse (AJADA) [14]. The amount of empirical work done on substance use in Kenya has expanded since these early years but has not been systematically summarized. The evidence gaps therefore remain unclear.

In order to guide future research efforts and adequately address the substance use scourge in Kenya, there is need to document the scope and breadth of available scientific literature. The aim of this systematic review is therefore: (i) to describe the characteristics of research studies conducted on substance use and SUD in Kenya; (ii) to assess the methodological quality of the studies; (iii) to identify areas where there is limited research evidence and; (iv) to make recommendations for future research. This paper is in line the Vision 2030 [15], Kenya’s national development policy framework, which directs that the government implements substance use treatment and prevention projects and programs, and target 3.5 of the Sustainable Development Goals (SDGs) which requires that countries strengthen the treatment and prevention for SUDs [16].

## Materials and methods

### Protocol and registration

In conducting this systematic review we adhered to the recommendations from the Preferred Reporting Items for Systematic Reviews and Meta-Analyses (PRISMA) statement [17]. A 27-item PRISMA checklist is available as an additional file to this protocol (S1 Checklist). Our protocol was registered in the International Prospective Register of Systematic Reviews (PROSPERO): CRD42020203717.

### Search strategy

A search was carried out in five electronic databases on 20^th^ August 2020: PubMed, PsychINFO, Web of Science, Cumulative Index of Nursing and Allied Professionals (CINAHL) and Cochrane Library. The full search strategy can be found in S1 File and takes the following form: *(terms for substance use) and (terms for substance use outcomes of interest) and (terms for region)*. The searches spanned the period from inception to date. No filter was applied. A manual search was done in Volumes 1, 2 and 3 (all published volumes by the time of the search) of the recently launched AJADA journal by NACADA, and additional articles identified.

[14, 18, 19].

### Study selection

Following the initial search, all articles were loaded onto Mendeley reference manager where initial duplicate screening and removal was done. After duplicate removal, the articles were loaded onto Rayyan, a soft-ware for screening and selecting studies during the conduct of systematic reviews [20]. The abstract and titles of retrieved articles were independently screened by two authors based on a set of pre-determined eligibility criteria. A second screening of full text articles was also done independently by two authors and resulted in an 88.7% agreement. Disagreements during each stage of the screening were resolved through discussion and consensus.

### Inclusion criteria

Since we sought to map existing literature on the subject, our inclusion criteria were broad. We included articles on substance use if (i) the sample or part of the sample was from Kenya, (ii) they were original research articles, (iii) they had a substance use or SUD exposure, (iv) they had a substance use or SUD related outcome such as prevalence, pattern of use, prevention and treatment, and (iv) they were published in English or had an English translation available. We included studies conducted among all age groups and studies that used all designs including quantitative, qualitative and mixed methods.

### Exclusion criteria

Studies were excluded if: (i) they were cross-national and did not report country specific results (ii) they did not report substance use or SUD as an exposure, and did not have substance use or SUD related outcomes or as part of the outcomes, (iii) they were review articles, dissertations, conference presentations or abstracts, commentaries or editorials, (iv) and the full text articles were not available.

### Data extraction

We prepared 3 data extraction forms based on three emerging categories of studies i.e.:

- Studies reporting on the epidemiology of substance use or SUD
- Studies evaluating substance use or SUD interventions and programs
- Studies qualitatively exploring various themes on substance use or SUD (but not evaluating interventions or programs)

The forms were piloted by F.J. and S.K. and adjustments made to the content. Data extraction was then done using the final form by all authors and double checked by F.J. for completeness and accuracy. Discrepancies were resolved by discussion with S.K. and E.T. until consensus was achieved. The following data was extracted for each study category:

1. Studies reporting on the epidemiology of substance use or SUD: study design, study population characteristics, study setting, sample size, age and gender distribution, substance(s) assessed, standardized tool or criteria used, main findings (prevalence, risk factors, other key findings).
2. Studies evaluating substance use or SUD interventions and programs: study design, study objective, sample size, name of the intervention or program, person delivering intervention, outcomes and measures, and main findings.
3. Studies qualitatively exploring various aspects of substance use or SUD other than programs and interventions: study objective, methods of data collection, study setting, study population, age and gender distribution, theoretical framework used, and main findings.

### Data synthesis

The results have been summarized descriptively and organized by the three categories above. Within each category, a general description of the study characteristics has been provided followed by a narrative synthesis of findings organized by sub-themes inductively derived from the data. The sub-themes within each category are as follows:

**Studies reporting on the epidemiology of substance use or SUD**: Epidemiology of alcohol use, epidemiology of tobacco use, epidemiology of khat use, epidemiology of cannabis use, epidemiology of opioid and cocaine use, epidemiology of other substance use (sedatives, inhalants, hallucinogens, prescription medication, emerging drugs, ecstasy).
**Studies evaluating substance use or SUD interventions and programs: *Individual level interventions*** (Individual-level interventions for harmful alcohol use, individual-level interventions for khat use, individual level intervention for substance use in general); ***Programs*** (Methadone programs, needle-syringe programs, tobacco cessation programs, out-patient SUD treatment programs); ***Population-level interventions***: Population-level tobacco interventions, population-level alcohol interventions.
**Studies qualitatively exploring various aspects of substance use or SUD other than programs and interventions**: Injecting drug use and heroin use, alcohol use, substance use among youth and adolescents, other topics.

### Quality assessment of the studies

Quality assessment was conducted by S.K. using the Quality Assessment Tool for Studies with Diverse Designs (QATSDD) [21]. F.J. & J.B. double checked the scores for completeness and accuracy. Any disagreements were discussed and resolved by consensus. We had initially planned to use the National Institute of Health (NIH) set of quality assessment tools but due to the diverse nature of study designs, the authors agreed to use the QATSDD tool. The QATSDD is a 16-item tool for both qualitative and quantitative studies. Each item is scored on a 4-point scale (0–3), with a total of 14 criteria for each study design and 16 for studies with mixed methods. Scoring relies on guidance notes provided as well as judgment and expertise from the reviewers. The criteria used are: (i) theoretical framework; (ii) statement of aims or objectives; (iii) description of research setting; (iv) sample size consideration; (v) representative sample of target group (vi) data collection procedure description; (vii) rationale for choice of data collection tool(s); (viii) detailed recruitment data; (ix) statistical assessment of reliability and validity of measurement tools (quantitative only); (x) fit between research question and method of data collection (quantitative only); (xi) fit between research question and format and content data collection (qualitative only); (xii) fit between research question and method of analysis; (xiii) justification of analytical method; (xiv) assessment of reliability of analytical process (qualitative only); (xv) user involvement in design and (xvi) discussion on strengths and limitations[21]. Scores are awarded for each criterion as follows: 0 = no mention at all; 1 = very brief description; 2 = moderate description; and 3 = complete description. The scores of each criterion are then summed up with a maximum score of 48 for mixed methods studies and 42 for studies using either qualitative only or quantitative only designs. For ease of interpretation, the scores were converted to percentages and classified as low (<50%), medium (50%–80%) or high (>80%) quality of evidence [22].

## Results

### Search results

**Fig 1:**
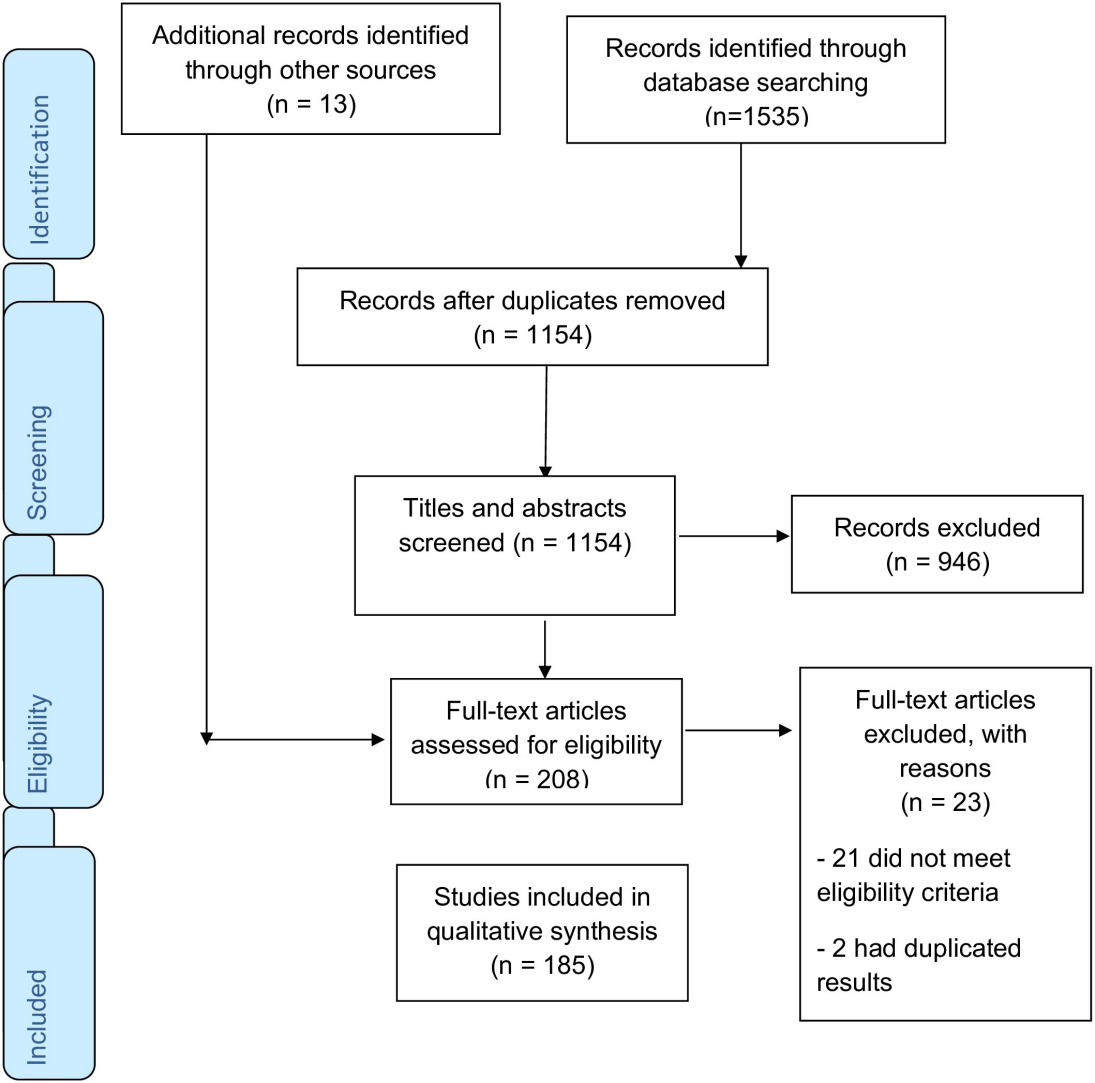
PRISMA flow chart.

The search from the five electronic databases yielded 1535 results: 950 from PubMed, 173 from PsychINFO, 210 from web of science, 123 from CINAHL and 79 from Cochrane library. Thirteen additional studies were identified through a manual search of the AJADA journals (Volumes 1, 2 and 3). Studies were assessed for duplicates and 1154 articles remained after removal of duplicates. The 1154 studies underwent an initial screening based on abstracts and titles, and 946 articles were excluded. A second screen of full text articles was done for the 208 studies that were potentially eligible for the review. Twenty three studies were excluded as follows: 21 did not meet the eligibility criteria and 2 had duplicated results. A total of 185 studies were found to meet the inclusion criteria and were included in the review (Fig 1).

### General characteristics of the studies

**Fig 2:**
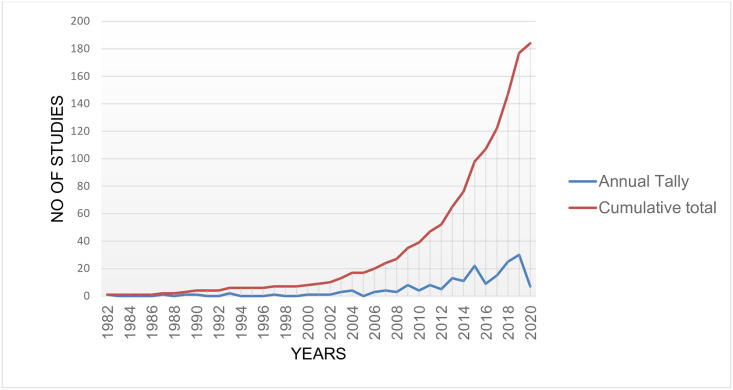
Line graph showing publication trends for substance use research in Kenya.

Of the 185 studies included in this review, 144 (77.8%) investigated the epidemiology of substance use or SUD, 18 (9.7%) evaluated substance use or SUD interventions and programs, and 23 (12.4%) were qualitative studies exploring perceptions on various substance use or SUD topics other than interventions and programs (Table 4). The studies were published between 1982 and 2020. The number of studies published has gradually increased in number over the years, particularly in the past decade. Fig 2 shows the publication trends for substance use research in Kenya.

### Quality assessment

The QATSDD scores ranged from 28.6% [23] to 92.9% [24]. Only 14 studies [12, 23, 25–36] (all quantitative) had scores of less than 50%. Of these, the main items driving low quality were: no mention of user involvement in study design (n = 14) [12, 23, 25–36], no explicit mention of a theoretical framework (n = 10) [12, 23, 25–28, 30, 33, 35, 36] and a lack of a statistical assessment of reliability and validity of measurement tools (n = 10) [12, 23, 25, 28, 30–33, 35, 36] Table 1.

**Table 1:** Quality assessment.

**Mixed Methods studies**																		
**Author, year**	**1**	**2**	**3**	**4**	**5**	**6**	**7**	**8**	**9**	**10**	**11**	**12**	**13**	**14**	**15**	**16**	**Total/48**	**Percentage of total**
Kamenderi et al., 2020 [37]	1	3	3	3	3	3	2	3	2	3	3	3	2	0	0	1	35	72.9
Mackenzie et al., 2009 [38]	1	3	1	3	3	3	3	3	2	3	3	3	3	0	0	0	34	70.8
Mutai et al., 2020 [39]	3	3	3	2	3	3	2	3	1	3	3	3	1	0	0	1	34	70.8
Papas et al., 2010 [40]	2	3	3	2	3	3	3	3	2	3	3	3	3	3	1	2	42	87.5
**Qualitative studies**																		
**Author, Year**	**1**	**2**	**3**	**4**	**5**	**6**	**7**	**8**	**9**	**10**	**11**	**12**	**13**	**14**	**15**	**16**	**Total/42**	**Percentage of total**
Bazzi et al., 2019 [41]	3	3	2	1	2	2	1	2	NA	NA	3	2	2	3	0	2	28	66.7
Beckerleg 2004 [42]	0	2	3	3	2	1	2	2	NA	NA	3	3	1	1	0	0	23	54.8
Ezard et al., 2011 [43]	2	3	2	2	3	3	2	3	NA	NA	3	3	3	3	1	0	33	78.6
Guise et al., 2015 [44]	2	3	3	3	3	3	3	3	NA	NA	3	3	3	3	0	2	37	88.1
Guise et al., 2019 [45]	3	3	2	2	2	2	1	2	NA	NA	3	3	2	3	0	1	29	69.0
Kibicho & Campbell, 2019 [46]	2	3	3	3	2	3	3	3	NA	NA	3	3	2	2	0	3	35	83.3
Mburu et al., 2018 [47]	1	3	3	2	3	2	3	3	NA	NA	3	3	2	3	0	2	33	78.6
Mburu et al., 2019 [48]	3	2	3	3	3	2	2	2	NA	NA	3	3	3	3	0	3	35	83.3
Mburu et al., 2020 [49]	1	2	3	3	3	3	2	3	NA	NA	3	3	3	3	0	2	34	81.0
Mburu et al., 2019 [50]	3	3	3	3	3	3	2	3	NA	NA	3	NA	3	3	0	3	35	83.3
Mburu, 2018[51]	3	3	3	3	3	3	3	3	NA	NA	3	NA	3	3	0	3	36	85.7
Mital et al., 2016 [52]	1	3	3	3	2	3	2	2	NA	NA	3	3	3	3	0	2	33	78.6
Muturi, 2014 [53]	2	3	1	3	3	2	2	2	NA	NA	3	3	3	3	0	3	33	78.6
Muturi, 2015 [54]	3	3	1	3	3	2	2	2	NA	NA	3	3	3	3	0	0	31	73.8
Muturi et al., 2016 [55]	3	3	1	3	3	2	2	2	NA	NA	3	3	3	3	0	2	33	78.6
Ndimbii et al., 2015 [56]	1	1	2	3	3	2	1	2	NA	NA	3	3	3	3	0	0	27	64.3
Ndimbii et al., 2018 [57]	1	3	2	3	3	3	3	3	NA	NA	3	3	2	3	2	3	37	88.1
Njue et al., 2009 [58]	0	2	2	3	2	2	1	2	NA	NA	3	3	2	2	2	2	28	66.7
Njue et al., 2011 [59]	0	3	2	3	3	3	2	2	NA	NA	3	3	2	2	2	0	30	71.4
Othieno et al., 2012 [60]	2	2	3	2	2	3	1	2	NA	NA	3	3	1	2	0	2	27	64.3
Rhodes et al., 2015 [61]	3	2	3	3	3	3	1	3	NA	NA	3	3	3	3	0	0	33	78.6
Rhodes, 2018 [62]	2	2	3	3	3	2	1	2	NA	NA	3	3	3	3	0	0	30	71.4
Ssewanyana et al., 2018 [63]	3	3	3	3	3	3	2	3	NA	NA	3	3	3	3	0	3	38	90.5
Syvertsen et al., 2016 [64]	0	2	2	2	3	1	0	1	NA	NA	3	3	2	2	0	1	22	52.3
Syvertsen et al., 2019 [65]	0	2	1	1	3	1	1	1	NA	NA	3	3	3	3	0	1	23	54.8
Velloza et al., 2015 [66]	3	3	3	3	3	3	2	3	NA	NA	3	3	3	3	0	3	38	90.5
Yotebieng et al., 2016 [67]	1	3	3	3	3	3	2	3	NA	NA	3	3	3	3	0	2	35	83.3
**Quantitative studies**																		
**First author, Year**	**1**	**2**	**3**	**4**	**5**	**6**	**7**	**8**	**9**	**10**	**11**	**12**	**13**	**14**	**15**	**16**	**Total/42**	**Percentage of total**
Aden et al., 2006 [23]	0	3	3	0	1	2	0	3	0	0	NA	0	0	NA	0	0	12	28.6
Akiyama et al., 2019 [68]	1	3	2	3	3	2	1	2	1	3	NA	3	3	NA	0	3	30	71.4
Anundo, 2019 [69]	3	3	3	2	2	3	3	2	2	3	NA	3	1	NA	0	0	30	71.4
Asiki et al., 2018 [70]	3	3	3	1	3	3	3	3	3	3	NA	3	3	NA	2	2	38	90.5
Astrom et al., 2004 [71]	0	3	3	3	3	2	3	1	1	3	NA	2	0	NA	0	0	24	57.1
Atwoli et al., 2011 [72]	1	3	3	3	3	3	3	3	1	3	NA	3	2	NA	0	2	33	78.6
Ayah et al., 2013 [73]	1	3	3	3	3	3	3	3	1	3	NA	3	2	NA	0	3	34	81.0
Ayaya et al., 2002 [74]	1	3	3	2	2	3	2	3	1	3	NA	3	2	NA	0	2	30	71.4
Balogun et al., 2014 [75]	2	3	2	1	3	1	1	1	1	3	NA	3	3	NA	0	3	27	64.3
Beckerlerg et al., 2006 [76]	0	3	3	2	3	3	1	3	2	3	NA	3	1	NA	0	0	27	64.3
Bengston et al., 2014 [77]	1	3	3	2	2	2	3	2	2	3	NA	3	2	NA	0	3	31	73.8
Budambula et al., 2018 [78]	1	3	1	3	3	3	2	2	2	3	NA	3	3	NA	3	3	35	83.3
Cagle et al., 2018 [79]	1	3	3	0	2	2	2	2	2	3	NA	2	2	NA	0	2	26	61.9
Chersich et al., 2014 [80]	3	3	3	2	2	3	3	3	2	3	NA	3	3	NA	0	3	36	85.7
Christensen et al., 2009 [81]	0	3	3	2	3	3	3	3	2	3	NA	3	3	NA	0	0	31	73.8
Cleland et al. 2007 [82]	0	3	3	0	0	3	3	3	0	3	NA	3	3	NA	0	3	27	64.2
De Menil et al., 2014 [83]	1	3	3	1	2	2	2	1	1	3	NA	2	1	NA	0	2	24	57.1
Deveau Dhadphale, 2010 [84]	1	2	3	2	3	2	1	3	1	3	NA	3	1	NA	0	0	25	59.5
Dhadphale et al., 1982 [12]	0	2	1	1	2	1	0	1	0	3	NA	3	0	NA	0	0	14	33.3
Dhadphale, 1997 [27]	0	3	1	1	2	1	1	1	2	3	NA	3	0	NA	0	0	18	42.9
Embleton, 2012 [85]	3	3	3	3	2	3	3	3	2	3	NA	3	3	NA	0	3	37	88.1
Embleton et al., 2013[86]	2	3	3	3	2	3	3	3	2	3	NA	3	3	NA	0	3	36	85.7
Embleton et al., 2017 [87]	1	3	3	3	2	3	3	3	2	3	NA	3	3	NA	0	3	35	83.3
Gathecha et al., 2018 [88]	2	3	2	2	3	3	3	3	2	3	NA	3	3	NA	0	3	35	83.3
Gichuki et al., 2015 [89]	3	3	3	3	2	3	3	3	3	3	NA	3	2	NA	1	2	37	88.1
Gitatui et al., 2019 [24]	2	3	3	3	2	3	3	3	3	3	NA	3	3	NA	3	2	39	92.9
Giusto et al., 2020 [90]	3	3	3	2	3	3	3	2	3	3	NA	3	3	NA	1	3	38	90.5
Goldblatt et al., 2015 [91]	2	3	3	2	2	3	3	3	3	3	NA	3	3	NA	1	3	37	88.1
Goodman et al., 2017 [92]	2	3	3	3	3	3	3	3	2	3	NA	3	3	NA	0	3	37	88.1
Hall et al., 1993 [93]	1	2	3	2	3	2	3	2	2	3	NA	3	3	NA	0	0	29	69.0
Harder et al., 2019 [94]	3	3	3	3	3	3	3	3	2	3	NA	3	3	NA	0	3	38	90.5
Haregu et al., 2019 [95]	1	3	3	3	3	3	2	3	2	3	NA	3	3	NA	0	2	34	81.0
Hulzebosch et al., 2015 [96]	1	3	3	2	3	3	2	3	2	3	NA	3	3	NA	0	3	34	81.0
Jenkins et al., 2017 [97]	1	3	3	2	2	3	3	3	2	3	NA	3	3	NA	0	2	33	78.6
Joshi et al., 2015 [98]	1	2	3	3	2	3	3	3	2	3	NA	3	3	NA	0	3	34	81.0
Kaai et al., 2019 [99]	1	2	2	2	3	2	2	2	1	3	NA	3	3	NA	0	2	28	66.7
Kaduka et al., 2017 [100]	0	3	3	3	3	2	2	1	2	3	NA	3	2	NA	0	0	27	64.3
Kamau et al., 2017 [101]	0	3	3	2	2	2	3	3	2	3	NA	3	2	NA	0	2	30	71.4
Kamenderi, 2019 [102]	1	3	2	3	3	1	1	2	1	3	NA	3	3	NA	0	0	26	61.9
Kamenderi et al., 2019 [103]	1	3	3	3	3	3	1	3	1	3	NA	3	2	NA	0	0	29	69.0
Kamenderi et al., 2019 [104]	1	3	3	3	3	3	2	3	2	3	NA	3	2	NA	0	1	32	76.2
Kamotho et al., 2004[105]	1	3	2	2	2	2	2	2	1	2	NA	3	2	NA	0	2	26	61.9
Kanyanya et al., 2007 [31]	2	3	3	1	3	1	0	1	0	3	NA	3	0	NA	0	0	20	47.6
Kaplan et al., 1990 [106]	1	2	3	2	3	2	1	2	0	3	NA	3	0	NA	0	1	23	54.8
Kendagor et al., 2018 [107]	2	3	3	2	3	2	3	2	2	3	NA	3	3	NA	0	2	33	78.6
Khasakhala et al., 2013 [108]	1	3	3	3	2	3	3	3	2	3	NA	3	3	NA	0	2	34	81.0
Khasakhala et al., 2013 [109]	1	2	3	3	2	3	3	3	2	3	NA	3	3	NA	0	2	33	78.6
Kiburi et al., 2018 [110]	1	3	3	2	2	2	3	2	2	3	NA	3	3	NA	0	2	31	73.8
Kimando et al., 2017 [111]	1	2	3	2	2	2	2	2	1	3	NA	3	3	NA	0	0	26	61.9
Kimani et al., 2019 [112]	1	3	3	3	2	2	1	2	0	3	NA	3	3	NA	0	2	28	66.7
Kimbui et al., 2018[113]	3	3	3	2	3	3	3	3	2	3	NA	3	3	NA	0	2	36	85.7
Kinoti et al., 2011 [114]	1	3	3	1	2	2	2	2	1	3	NA	3	0	NA	0	2	25	59.5
Kinyanjui & Atwoli, 2013 [115]	0	3	3	2	3	3	3	3	2	3	NA	3	2	NA	0	2	32	76.2
Kisilu et al., 2019 [29]	1	3	1	2	2	1	1	1	1	2	NA	3	1	NA	0	0	19	45.2
Komu et al., 2009[116]	0	3	2	2	2	1	1	1	0	3	NA	3	1	NA	0	2	21	50.0
Korhonen et al., 2018 [117]	1	2	3	1	3	3	3	3	2	3	NA	3	3	NA	0	3	33	78.6
Kunzweiler et al., 2017 [118]	0	3	3	2	0	3	0	3	0	3	NA	3	3	NA	0	3	26	61.9
Kunzweiler et al., 2018 [119]	2	3	3	3	3	3	3	3	2	3	NA	3	3	NA	0	3	37	88.1
Kuria et al., 2012 [120]	1	3	3	2	2	3	3	3	1	3	NA	3	2	NA	0	2	31	73.8
Kurth et al., 2015 [121]	1	3	3	2	3	3	3	3	1	3	NA	3	3	NA	0	2	33	78.6
Kurui & Ogoncho, 2019 [122]	1	3	3	3	2	2	1	2	0	3	NA	3	1	NA	0	0	24	57.1
Kurui & Ogoncho, 2020 [123]	1	3	2	2	3	1	1	1	1	3	NA	3	1	NA	0	0	22	52.4
Kwamanga et al., 2001 [124]	0	3	3	3	3	2	1	2	0	3	NA	3	1	NA	3	0	27	64.3
Kwamanga et al., 2003 [125]	1	3	3	2	3	2	1	2	0	2	NA	3	1	NA	0	0	23	54.8
Kwobah et al., 2017 [126]	0	3	3	3	3	3	3	3	2	3	NA	3	2	NA	0	2	33	78.6
L’Engle et al., 2014 [127]	1	3	3	3	3	3	3	3	2	3	NA	3	3	NA	0	0	33	78.6
Lo et al., 2013 [128]	1	3	3	3	3	3	1	3	1	3	NA	3	2	NA	0	3	32	76.2
Luchters et al., 2011 [129]	1	3	3	2	3	3	3	2	2	3	NA	3	3	NA	0	2	33	78.6
Lukandu et al., 2015 [130]	1	3	1	1	3	3	1	3	0	3	NA	3	1	NA	0	0	23	54.8
Macigo et al., 2006 [32]	1	3	1	1	2	2	0	2	0	3	NA	3	1	NA	0	1	20	47.6
Magati et al., 2018 [131]	0	3	2	3	3	1	1	1	0	3	NA	3	3	NA	0	0	23	54.8
Maina et al., 2015 [132]	1	3	3	3	2	2	3	2	2	3	NA	3	2	NA	0	2	31	73.8
Mannik et al., 2018 [133]	0	2	3	2	3	2	1	2	2	3	NA	3	2	NA	0	3	28	66.7
Maru et al., 2003 [30]	0	3	3	2	3	1	0	1	0	3	NA	3	0	NA	0	0	19	45.2
Mburu et al., 2018 [134]	2	2	2	2	2	1	1	1	1	3	NA	3	3	NA	0	2	25	59.5
Medley et al., 2014 [135]	1	2	1	2	3	3	2	2	1	3	NA	3	3	NA	0	2	28	66.7
Menach et al., 2012 [136]	1	3	3	2	2	3	2	2	1	3	NA	3	1	NA	0	1	27	64.3
Menya et al., 2019 [137]	1	2	3	2	3	3	1	3	0	3	NA	3	3	NA	0	3	30	71.4
Micheni et al., 2015 [33]	0	2	1	1	3	1	0	1	0	3	NA	3	3	NA	0	2	20	47.6
Mkuu et al., 2018 [138]	1	3	2	3	3	2	2	2	1	3	NA	3	3	NA	0	2	30	71.4
Mohammed et al., 2018 [139]	0	3	3	3	3	2	2	2	2	3	NA	3	3	NA	0	3	32	76.2
Mokaya et al., 2016 [140]	1	3	3	3	3	3	3	3	1	2	NA	3	3	NA	0	0	31	73.8
Moscoe et al., 2019 [141]	1	2	2	3	2	2	1	2	1	3	NA	3	3	NA	0	3	28	66.7
Mundan et al., 2013 [142]	1	2	3	2	2	2	2	2	1	3	NA	3	3	NA	0	3	29	69.0
Mungai & Midigo, 2019 [143]	1	3	3	2	3	1	1	1	0	3	NA	3	0	NA	0	0	21	50.0
Muraguri et al., 2015 [144]	0	3	2	2	3	2	0	1	0	3	NA	3	0	NA	0	2	21	50.0
Muriungi & Ndetei, 2013[145]	3	3	3	3	3	3	3	3	1	3	NA	3	3	NA	0	2	36	85.7
Muthumbi et al., 2017 [146]	1	2	2	2	3	2	0	2	0	3	NA	3	3	NA	0	3	26	61.9
Mutiso et al., 2019 [147]	3	3	3	3	3	3	3	3	2	3	NA	3	3	NA	0	3	38	90.5
Muture et al., 2011 [148]	1	3	2	2	3	2	1	2	0	3	NA	3	3	NA	0	1	26	61.9
Mwangi et al., 2019[149]	2	3	3	3	3	3	3	3	1	3	NA	3	3	NA	0	3	36	85.7
Nall et al., 2019 [150]	2	3	1	3	3	2	2	2	1	3	NA	3	3	NA	0	3	31	73.8
Ndegwa & Waiyaki, 2020 [151]	2	3	3	3	2	3	2	2	1	3	NA	3	1	NA	0	0	28	66.7
Ndetei et al., 2008 [28]	0	3	3	0	3	1	1	1	0	3	NA	3	0	NA	0	0	18	42.9
Ndetei et al., 2008 [152]	1	1	3	1	2	1	2	1	1	3	NA	3	3	NA	0	0	22	52.4
Ndetei et al., 2009 [153]	0	3	3	2	3	2	1	2	1	3	NA	3	1	NA	0	0	24	57.1
Ndetei et al., 2009 [154]	0	3	3	2	3	2	1	2	0	3	NA	3	2	NA	0	0	24	57.1
Ndetei et al., 2010 [34]	1	2	1	2	2	1	2	1	1	3	NA	3	1	NA	0	0	20	47.6
Ndetei et al., 2012 [155]	0	3	3	0	0	3	3	3	0	3	NA	3	3	NA	0	3	27	64.2
Ndugwa et al., 2011 [156]	3	3	3	3	3	3	3	2	2	3	NA	3	3	NA	0	3	37	88.1
Ng’ang’a et al., 2018 [157]	1	3	3	3	2	3	3	2	2	3	NA	3	3	NA	0	2	33	78.6
Ngaruyia et al., 2018 [158]	1	3	3	3	3	3	3	3	2	3	NA	3	3	NA	0	2	35	83.3
Nguchu et al., 2009 [159]	0	3	3	2	2	3	2	3	2	3	NA	3	1	NA	0	1	28	66.7
Ngure et al., 2019 [160]	1	3	2	3	3	2	2	2	2	3	NA	3	1	NA	0	0	27	64.3
Nielsen et al., 1989 [161]	0	3	1	1	2	2	3	2	3	3	NA	3	1	NA	0	0	24	57.1
Njoroge et al., 2017 [162]	0	2	2	2	3	2	1	2	0	3	NA	3	3	NA	0	0	23	54.8
Njuguna et al., 2013 [25]	0	1	3	1	2	1	0	1	0	3	NA	3	0	NA	0	1	16	38.1
Ogwell et al., 2003 [163]	0	3	3	3	3	3	1	3	0	3	NA	3	3	NA	0	2	30	71.4
Okal et al., 2013 [164]	0	1	2	3	3	3	1	3	0	3	NA	3	1	NA	0	3	26	61.9
Olack et al., 2015 [165]	1	3	3	2	2	2	3	2	1	3	NA	3	2	NA	0	3	30	71.4
Ominde et al., 2019 [35]	0	3	2	2	2	2	1	1	0	3	NA	3	1	NA	0	0	20	47.6
Omolo & Dhadphale, 1987 [36]	0	3	3	2	2	2	0	2	0	3	NA	3	0	NA	0	0	20	47.6
Ongeri et al., 2019 [166]	1	3	3	3	3	3	3	3	2	3	NA	3	3	NA	0	2	35	83.3
Onsomu et al., 2015[167]	1	2	2	3	3	3	2	2	2	3	NA	3	3	NA	0	2	31	73.8
Othieno et al., 2000 [168]	0	3	2	2	3	2	1	2	1	3	NA	3	1	NA	0	0	23	54.8
Othieno et al., 2014[169]	1	3	3	3	2	2	1	2	1	3	NA	3	3	NA	0	2	29	69.0
Othieno et al., 2015a[170]	1	3	3	3	2	2	1	2	1	3	NA	3	3	NA	0	2	29	69.0
Othieno et al., 2015b [171]	1	3	3	2	3	2	2	2	2	3	NA	3	3	NA	0	2	31	73.8
Owuor et al., 2019 [172]	3	3	3	3	3	3	3	2	3	3	NA	3	2	NA	1	1	36	85.7
Oyaro et al., 2018 [173]	0	3	3	2	3	3	1	2	1	3	NA	3	1	NA	0	0	25	59.5
Pack et al., 2014 [174]	1	3	3	2	3	3	2	2	2	3	NA	3	2	NA	0	3	32	76.2
Papas et al., 2011 [175]	2	3	3	2	3	3	3	3	2	3	NA	3	3	NA	1	2	36	85.7
Papas et al., 2016[176]	1	3	3	2	2	3	3	3	2	3	NA	3	3	NA	0	2	33	78.6
Papas et al., 2017 [177]	2	3	3	3	2	3	3	3	3	3	NA	3	3	NA	3	3	40	95.2
Parcesepe et al., 2016 [178]	1	3	3	2	3	3	3	3	2	3	NA	3	3	NA	0	2	34	81.0
Patel et al., 2013 [179]	1	3	2	2	3	1	1	1	0	3	NA	3	1	NA	0	0	21	50.0
Peltzer et al., 2009 [180]	1	3	2	2	3	3	3	2	2	3	NA	3	3	NA	0	3	33	78.6
Peltzer et al., 2011 [181]	1	3	1	2	3	3	1	2	1	3	NA	3	3	NA	0	2	28	66.7
Pengpid & Peltzer, 2019 [182]	0	3	2	2	3	3	3	2	2	3	NA	3	3	NA	0	2	31	73.8
Perl et al., 2015 [183]	1	2	1	2	3	2	1	2	0	3	NA	3	3	NA	0	3	26	61.9
Ploubidis, 2013 [184]	3	3	3	2	3	2	1	2	0	3	NA	3	3	NA	0	3	31	73.8
Roth et al., 2017 [185]	1	2	3	2	3	2	1	2	0	3	NA	3	2	NA	0	2	26	61.9
Rudatsikira et al., 2007 [186]	0	2	2	2	3	2	1	2	0	3	NA	3	3	NA	0	2	25	59.5
Sanders et al., 2007 [187]	0	2	2	3	3	2	1	2	0	3	NA	3	3	NA	0	3	27	64.3
Saunders et al., 1993 [188]	1	3	2	2	3	3	2	3	2	3	NA	3	3	NA	0	0	30	71.4
Secor et al., 2015 [189]	2	3	2	2	3	3	2	2	2	3	NA	3	2	NA	0	2	31	73.8
Syvertsen et al., 2015 [190]	0	3	3	0	0	3	0	2	0	3	NA	3	3	NA	0	3	23	54.8
Shaffer et al., 2004 [191]	0	2	3	2	3	2	2	2	1	3	NA	3	1	NA	0	1	25	59.5
Takahashi et al., 2017 [192]	0	3	3	3	3	3	3	3	3	3	NA	3	3	NA	0	3	36	85.7
Takahashi et al., 2018 [193]	2	3	3	3	2	3	2	3	1	3	NA	3	3	NA	0	2	33	78.6
Tang et al., 2018 [194]	1	3	2	1	3	2	1	2	3	0	NA	3	2	NA	0	3	26	61.9
Tegang et al., 2010[195]	0	3	3	2	3	3	2	2	3	2	NA	3	3	NA	0	1	30	71.4
Thuo et al., 2008 [26]	0	3	2	1	2	1	1	1	3	0	NA	3	0	NA	0	0	17	40.5
Tsuei et al., 2017 [196]	0	3	2	2	2	2	1	2	3	0	NA	3	3	NA	0	2	25	59.5
Tun et al., 2015 [197]	1	2	3	2	2	3	2	3	3	1	NA	3	2	NA	0	3	30	71.4
Wekesah et al., 2018 [198]	1	2	3	2	2	2	2	2	3	1	NA	3	3	NA	0	2	28	66.7
Were et al., 2014 [199]	1	3	2	2	3	2	1	2	3	1	NA	3	1	NA	0	1	25	59.5
White et al., 2016 [200]	1	2	3	2	2	2	2	2	3	1	NA	3	2	NA	0	3	28	66.7
Widmann et al., 2014 [201]	1	3	3	2	3	3	3	3	3	3	NA	3	3	NA	2	3	38	90.5
Widmann et al., 2017 [202]	2	3	3	2	3	3	3	2	3	3	NA	3	3	NA	2	3	38	90.5
Wilson et al., 2016 [203]	1	2	1	3	3	3	3	2	3	3	NA	3	3	NA	3	3	36	85.7
Winston et al., 2015 [204]	1	3	2	3	2	3	2	2	3	2	NA	3	3	NA	0	3	32	76.2
Winter et al., 2020 [205]	2	3	3	2	2	3	3	2	3	1	NA	3	3	NA	1	3	34	81.0
Woldu et al., 2019 [206]	1	3	3	3	2	3	3	3	2	3	NA	3	3	NA	3	3	38	90.5

### Studies examining the epidemiology of substance use or SUD

#### General description of epidemiological studies

One hundred and forty-four studies examined the prevalence and or risk factors for various substances. The studies were published between 1982 and 2020. The four main study designs used were cross-sectional (n = 126), cohort (n = 5), case-control (n = 10), and mixed methods (n = 2). One study used a combination of the multiplier method, Wisdom of the Crowds (WOTC) method, and a published literature review to document the size of key populations [164]. The sample size for this category of studies ranged from 42 [130] to 72292 [128].

The studies were conducted in diverse settings including the community (n = 72), hospitals (n = 40), institutions of learning (n = 24), streets (n = 5), prisons and courts (n = 3), charitable institutions (n = 1), methadone maintenance therapy (MMT) clinics (n = 1), and in needle-syringe program (NSP) sites (n = 1). Of the studies conducted within the community, 12 were conducted in informal settlements. The study populations were similarly diverse as follows: general population adults & adolescents (n = 39), persons with NCDs (n = 11), primary and secondary school students (n = 15), people who inject drugs (PWID) (n = 11), general patients (n = 5), men who have sex with men (MSM) (n = 8), university and college students (n = 9), commercial sex workers (n = 7), psychiatric patients (n = 6), orphans and street connected children and youth (n = 6), people living with HIV (PLHIV) (n = 6), healthcare workers (n = 3), law offenders (n = 3), military (n = 1), and teachers (n = 1). Only one study was conducted among pregnant women [131].

Sixty-nine studies (47.6%) used a standardized diagnostic tool to assess for substance use. The Alcohol Use Disorder Identification Test (AUDIT) (n = 21) and the Alcohol, Smoking & Substance Use Involvement Screening Test (ASSIST) questionnaire (n = 10) were the most frequently used tools. Most papers assessed for alcohol (*n* = 109) and tobacco use (*n* = 80). Other substances assessed included khat (n = 34), opioids (n = 21), sedatives (n = 19), cocaine (n = 19), inhalants (n = 16), cannabis (n = 14), hallucinogens (n = 7), prescription medication (n = 4), emerging drugs (n = 1) and ecstasy (n = 1). Most studies (n = 93) assessed for more than one substance.

#### Epidemiology of alcohol use

One hundred and nine papers assessed for the prevalence and or risk factors for alcohol use. Using the AUDIT, the 12-month prevalence rate for hazardous alcohol use ranged from 2.9% among adults drawn from the community [97] to 64.6% among female sex workers (FSW) [77]. Based on the same tool, the lowest and highest 12-month prevalence rates for harmful alcohol use were both reported among FSWs i.e. 9.3% [80] and 64.0% [174] respectively, while the prevalence of alcohol dependence ranged from 8% among FSWs living with HIV [203] to 33% among MSM who were commercial sex workers [144]. The highest lifetime prevalence rate for alcohol use was reported by Ndegwa & Waiyaki [151]. The authors found that 95.7% of undergraduate students had ever used alcohol.

Alcohol use, was associated with several socio-demographic factors including being male [50, 112, 114, 140, 158, 168, 182, 191], being unemployed [114], being self-employed [97], having a lower socio-economic status (SES) [128], being single or separated, living in larger households [97], having a family member struggling with alcohol use, and alcohol being brewed in the home [143]. Alcohol use was linked to various health factors including glucose intolerance [81], poor cardiovascular risk factor control [111], having a diagnosis of diabetes mellitus [134], hypertension [112, 139], default from tuberculosis (TB) treatment [148], depression [113], psychological Intimate Partner Violence (IPV) [205], tobacco use [182, 205], and increased risk of esophageal cancer [137, 179]. Finally, alcohol use was associated with involvement in Road Traffic Accidents (RTAs) [88], and having injuries [88, 171] and suicidal behavior [109].

#### Epidemiology of tobacco use

Eighty papers assessed for the prevalence and risk factors for tobacco use. The lifetime prevalence of tobacco use ranged from 23.5% among healthcare workers (HCWs) [140] to 84.3% among psychiatric patients [110]. The highest lifetime prevalence rate for tobacco use was reported by Ndegwa & Waiyaki [151]. The authors found that 95.7% of undergraduate students had ever used tobacco.

Tobacco use was associated with socio-demographic factors such as being male [112, 140, 168] and living in urban areas [163]. Several health factors were linked to tobacco use including hypertension [112], development of oral leukoplakia [32], pneumonia [146], increased odds of laryngeal cancer [136], ischemic stroke [100] and diabetes mellitus [134]. In addition, tobacco use was associated with having had an injury in the last 12 months [171], emotional abuse [110], and psychological IPV [205]. Longer duration of smoking was associated with a diagnosis of diabetes mellitus [73], lower SES [128], and hypertension [98, 142]. Peltzer et al. [181] reported that early smoking initiation among boys was associated with ever drunk from alcohol use, ever used substances, and ever had sex. Among girls, the authors found that early smoking initiation was associated with higher education, ever drunk from alcohol use, parental or guardian tobacco use, and suicide ideation.

#### Epidemiology of khat use

The epidemiology of khat use was investigated by 34 studies. The lifetime prevalence rate for khat use ranged from 10.7% among general hospital patients [168] to 88% among a community sample [23]. Khat use was associated with being male [114, 168]; unemployment [114]; being employed [25]; younger age (less than 35 years), higher level of income, comorbid alcohol and tobacco use [166] and age at first paid sex of less than 20 years among FSWs [195]. Further, khat use was associated with increased odds of negative health outcomes [130, 146, 166, 201].

Higher odds of reporting psychotic [166, 201], and PTSD (Post-Traumatic Stress Disorder) symptoms [201], having thicker oral epithelium [130], and pneumonia [146], were reported among khat users compared to non-users.

#### Epidemiology of cannabis use

Fourteen studies evaluated the prevalence of cannabis use. The lifetime prevalence rate of cannabis use ranged from 21.3% among persons with AUD [120] to 64.2% among psychiatric patients [110]. Cannabis use was associated with being male [140, 168], and with childhood exposure to physical abuse [110].

#### Epidemiology of opioid and cocaine use

Twenty-one studies investigated the prevalence of opioid use. The lifetime prevalence rate of opioid use ranged from 1.1% among PLHIV [132] to 8.2% among psychiatric patients [110].

Nineteen studies assessed for the prevalence of cocaine use. The highest reported prevalence rates were 76.2% among PWID use (current use) [190]; 8.8% among healthcare workers (lifetime use) [140]; and 6.7% among PLHIV (lifetime use) [132].

#### Epidemiology of IDU

One study assessed the prevalence for IDU. Key population size estimates for PWID use was reported as 6107 for Nairobi [164]. IDU was associated with depression, risky sexual behavior [149], Hepatitis-C Virus (HCV) infection [173], and HIV-HCV co-infection [68].

#### Epidemiology of other substance use (sedatives, inhalants, hallucinogens and prescription medication, emerging drugs, ecstasy)

The epidemiology of sedative use was investigated by 19 studies, inhalant use by 16 studies, hallucinogen use by 7 studies, prescription medication by 4 studies, and emerging drugs and ecstasy by one study each. The highest lifetime prevalence rate for sedative use was reported as 71.4% among a sample of psychiatric patients [28], while the highest prevalence rate for inhalant use was 67% among children living in the streets [86]. The lifetime prevalence rates for hallucinogen use ranged from 1.4% among university students [160] to 3.7% among psychiatric patients [110]. The highest prevalence rate for the use of prescription medication was reported as 21.2% among PWID [190]. One study each reported on the prevalence of emerging drugs [122] and ecstasy [153]. The studies were both conducted among adolescents and youth. The authors found the lifetime prevalence rates for the two substances to be 11.8% [122] and 4.0% [153] respectively.

#### Other topics explored by the epidemiology studies

In addition to prevalence and associated factors, the epidemiological studies explored other topics.

Papas et al. [176] explored the agreement between self-reported alcohol use and the biomarker phosphatidyl ethanol and reported a lack of agreement between self-reported alcohol use and the biomarker phosphatidyl ethanol among PLHIV with AUD.

One study investigated the self-efficacy of primary HCWs for SUD management and reported that self-efficacy for SUD management was lower in those practicing in public facilities and among those perceiving a need for AUD training. Higher self-efficacy was associated with attending to a higher proportion of patients with AUD, and the belief that AUD is manageable in outpatient settings [196].

Five studies investigated the reasons for substance use. Common reasons for substance use included leisure, stress and peer pressure among psychiatric patients[28], curiosity, fun, and peer influence among college students [123], peer influence, idleness, easy access, and curiosity among adults in the community [25], and peer pressure, to get drunk, to feel better and to feel warm among street children [74]. Atwoli et al. 2011 [72] reported that most students were introduced to substances by friends.

Kaai et al. [99] conducted a study regarding quit intentions for tobacco use and reported that 28% had tried to quit in the past 12 months, 60.9% had never tried to quit, and only 13.8% had ever heard of smoking cessation medication. Intention to quit smoking was associated with being younger, having tried to quit previously, perceiving that quitting smoking was beneficial to health, worrying about future health consequences of smoking, and being low in nicotine dependence. A complete description of the prevalence studies has been provided in Table 2.

**Table 2:** Studies reporting on the epidemiology of substance use or SUDs.

Author, Year	Study design	Study population/study setting	Sample size	Age; gender distribution	Substance(s) assessed	Standardized tool/criteria used	Main findings (prevalence, risk factors, other key findings)
Dhadphale et al. 1982 [12]	Cross-sectional	Students (Secondary school)	2870	Age range: 14–20 years Male to female ratio 2:1	Alcohol, tobacco, cannabis	None	Prevalence of tobacco use 3 or more times a week—16.1% Prevalence of alcohol use 3 or more times a week—10.3% Prevalence of cannabis use was 13.5% at a rate of 1 time per month
Omolo & Dhadphale 1987 [36]	Cross-sectional	General patients (Hospital)	100	Age distribution not reported Males 50%	Khat^a^	None	Lifetime prevalence khat use was 29%. Mild and moderate chewing significantly associated with age < 20 years (p<0.001)
Nielsen et al. 1989 [161]	Cross-sectional	Outpatients (Hospital)	112	18–65 years Males 50%	Alcohol	DSM-III	30 patients met the criteria for both alcohol abuse and alcohol dependence. 8 patients received a diagnosis of alcohol abuse only and 6 patients received a diagnosis of alcohol dependence only. 39% of the sample exceeded the cut off score for one or both DSM diagnoses
Kaplan et al. 1990 [106]	Cross-sectional	Adults (Community)	Not indicated	Age range: 20–40 years Gender distribution not reported	Tobacco	None	Highest prevalence lifetime tobacco smoking was by luo community: 63% males and 67% females Reasons for smoking: positive feelings, to work harder
Hall et al. 1993 [93]	Cross-sectional	General patients (Hospital)	105	Mean age: 35.4 years Males 78.1%	Alcohol	DSM-III-R/ ICD-I0.	Prevalence of weekly alcohol use was 48%
Saunders et al. 1993 [188]	Cross-sectional	General patients (Hospital)	Country specific sample size not reported	Country specific demographics not reported	Alcohol	None	Prevalence of alcohol use for Kenya ->40g per day was 43%, and >60g per day was 37%
Dhadphale 1997 [27]	Cross-sectional	Psychiatric patients (Hospital)	220	Age range: 18–55 years Males 50.9%	Alcohol	MAST and ICD-9 criteria	Lifetime prevalence of alcohol use among patients with psychiatry morbidity was 12.7% (and 3.1% of those attending outpatient care)
Othieno et al. 2000 [168]	Cross-sectional	General patients (Hospital)	150	Modal age group: 20–39 years Males 50%	Alcohol, tobacco, khat, cannabis, methaqualone	DSM IV Criteria	Lifetime prevalence: alcohol use (56.7%), tobacco use (32%), khat use (10.7%), cannabis use (5.3%), methaqualone use (0.7%) Alcohol use (p = 0.000), tobacco use (p = 0.000), khat use (p = 0.045), cannabis use (p = 0.004) associated with being male
Ayaya et al. 2001 [74]	Case-control	Children living in the streets	191	Mean age: 14.03 (SD2.4) Gender distribution not reported	Alcohol, tobacco, cannabis, glue, cocaine	None	Prevalence for drug abuse was 545 per 1000 children. Specific substance prevalence: tobacco 37.6%; sniffing glue 31.2%; alcohol 18.3%; cannabis 8.3%; and sniffing cocaine 4.6% Reasons for substance use included peer pressure, to get drunk, to feel better and to feel warm
Kwamanga et al. 2001 [124]	Cross-sectional	Teachers (School)	800	Median age: 35 years, Males 74.5%	Tobacco smoking	WHO standard self- administered questionnaire	50% of males and 3% of females reported tobacco smoking. Peer pressure (63%) and advertisements (21%) are major drivers of smoking
Christensen et al. 2009 [81]	Cross-sectional	Adults (Community)	1179	Mean age: 38.6 years Males 42%	Alcohol, tobacco	None	Tobacco use was 6.6% in females and 16.2% in males; Alcohol use was 5.4% in females and 20.9% in males Daily alcohol use in males associated with glucose intolerance (p<0.01)
Kwamanga et al. 2003 [125]	Cross-sectional	Students (Secondary school)	5311	Mean age: 16.7 years, Males 68.1%	Tobacco smoking	A WHO standard self- administered questionnaire	Prevalence of current smoking was 10.5%. A total of 12.4% of male students and 6.4% of female students were current smokers. Smoking associated with older age (p<0.001), being in a private school (p<0.001). Reduced odds of stopping smoking with increase in number of tobacco smoked (OR 0.22; 95% CI = 0.19, 0.26; p<0.001)
Maru et al. 2003 [30]	Cross-sectional	Children and youth (Juvenile court)	90	Age range: 8–18 years Males 71.1%	Alcohol, khat, tobacco, volatile hydrocarbons, sedatives, cannabis	None	Overall prevalence of substance use 43.3%. Tobacco 32.2%; volatile hydrocarbons 21.1%; cannabis 8.9%; alcohol 6.7%; khat 5.6%; sedatives 3.3% Substance use associated with being male (p = 0.0134)
Ogwell et al. 2003 [163]	Cross-sectional	Pupils (Primary school)	1130	Mean age: 14.1 (SD 0.9) years Males 52%	Tobacco	None	Lifetime tobacco use was 31%, lifetime use of smokeless tobacco was 9%, 55% had friends who smoked Rates of lifetime smoking higher in urban than in suburban students (p<0.005)
Astrom et al. 2004 [71]	Cross-sectional	Pupils (Primary school)	1130	Mean age: 14.1 (SD 0.9) years Males 52%	Tobacco	None	Tobacco smoking; 31% reported ever smoking tobacco Sources of anti-tobacco messages: broadcast media (47%), Newspapers and magazines (45%), schoolteachers (32%), health workers (29%)
Kamotho et al. 2004 [105]	Cross-sectional	Patients undergoing coronary angiography (Hospital)	144	Coronary artery disease (CAD): Mean age: 54.4 years, male to female ratio -5.5:1; No CAD: Mean age: 49.8 years, male to female ratio 2.3:1	Alcohol, tobacco smoking,	None	CAD: Smoking prevalence 15.4%, alcohol 32.7; No CAD: smoking prevalence 13.0%, alcohol 36.9% There was no difference in prevalence of smoking (p = 0.227) and alcohol use (p = 0.67) between those with CAD and those without
Shaffers et al. 2004 [191]	Cross-sectional	General patients (Hospital)	299	Mean age: 38 (SD 8) years Males 55%	alcohol	AUDIT	Prevalence of hazardous drinking 53.5%, (males 76. %, female 25%), Being male associated with hazardous drinking (p = 0.01)
Aden et al. 2006 [23]	Cross-sectional	Adults (Community)	50	Age range: 15–34 years Males 80%	Khat	None	Prevalence of khat use was 88%
Beckerleg et al. 2006 [76]	Cross- sectional	Adults (Community)	496	Age data not given Males 95%	Heroin	None	Prevalence of lifetime heroin injection was 15%; current injection was 7% Average number of years of heroin use was 11.1 years
Macigo et al. 2006 [32]	Case-control	Adults and adolescents (Community)	226	Age: 15 years and above Males 100%	Tobacco	None	Smoking tobacco was associated with development of oral leukoplakia among those who brushed (RR 4.6 95%CI 2.9–5.1 p<0.001) and those who did not brush teeth (RR 7.3 95%CI 3.6–16.3 p<0.001)
Cleland et al. 2007 [82]	Cross-sectional	PWID use (Community)	106	Mean age (SD): Males 29 [7]; Female 28 [8] Males 87%	Injection drugs (not specified)	None	Receptive sharing 26% Distributive sharing 41%
Kanyanya et al. 2007 [31]	Cross-sectional	Inmates (Prison)	76	Mean age: 33.5 years Males 100%	alcohol	DSM-IV Criteria	71.1% had lifetime abuse or dependence of alcohol
Rudatsikira et al. 2007 [186]	Cross-sectional study	Pupils (Primary school)	242	Age: 54.3% aged >15 years Gender distribution: males 55.7%	Alcohol, tobacco smoking, other drugs (not specified)	None	Lifetime use: alcohol 10.7%, smoking 10.3%, other drugs 8.4% Past month use: alcohol 9.1%, smoking 6.0% The risk factors for having sex among males were: ever smoked (OR = 2.05, 95%CI 1.92, 2.19), currently drinking alcohol (OR = 1.13, 95%CI 1.06, 1.20), ever used drugs (OR = 2.36, 95%CI 2.24, 2.49) and among females ever used drugs (OR = 2.85, 95%CI 2.57, 3.15).
Sanders et al. 2007 [187]	Cross-sectional study	Men who have Sex with Men Exclusively (MSME) and Men who have Sex with both Men and Women (MSMW) (Community)	285	Median age (IQR): MSME 27 [23–29]; MSMW 28[23–35] all males	Injection drugs (not specified)	None	Prevalence of IV drug use among MSME was 0.9% and among MSMW was 1.8%
Ndetei et al. 2008 [28]	Cross-sectional	Psychiatric Patients (Hospital)	691	78% aged between 21–45 years Males: 63%	Alcohol, opioid, sedatives, khat	SCID-I for DSM IV	Prevalence substance abuse disorder—34.4%. Alcohol use disorder (52%), opiate use disorder (55.5%), sedative use disorder (71.4%), khat use disorder (58.8%) Leisure, stress and peer pressure were the most common reasons given for abusing substances
Ndetei et al. 2008 [152]	Cross-sectional	Adults (Community)	1420	Mean age: 29.2 years Gender distribution not reported	Alcohol, tobacco, khat, cocaine, heroin, sedatives, opioids, inhalants, phencyclidine, prescription pills, amphetamines	None	Alcohol use prevalence was 36.3% and cocaine 2.2% (most and least abused substances nationally). Prevalence of other substances not stated. Reasons for substance use: leisure, stress and peer pressure
Thuo et al. 2008 [26]	Cross-sectional	Psychiatric Patients (Hospital)	148	Mean age: 31 years Males nearly two-thirds	Alcohol	SCID for DSM IV	More males (*n* = 39) than females (*n* = 6) were abusing substances (*p<*0.001); Significant associations between PDs and substance abuse dependence (*p<*0.001)
Komu et al. 2009 [116]	Cross-sectional	Students (University)	281	Age data not given Males 60.4%	Tobacco smoking	None	Prevalence of current tobacco smoking was 12.1% and lifetime prevalence was 38%
Ndetei et al. 2009 [153]	Cross-sectional	Students (Secondary school)	1252	Mean age: 17 years males 62.5%	Alcohol, tobacco, amphetamines, sedatives, cannabis, hallucinogens, cocaine, methaqualone, ecstasy, heroin, inhalants.	School Toolkit by UNODC	Lifetime smoking reported by 25,3%, daily smoking reported by 3.9% Lifetime use: alcohol 19.6%; heroin 4.0%, amphetamines 18.3%, sedatives 7.0%, cannabis 7.1%, hallucinogen 4.1%, cocaine 4.2%, mandrax 4.0%, ecstasy 4.0%, inhalants 6.6% Age at first use as low as below 11 years
Ndetei et al. 2009 [154]	Cross-sectional	Students (Secondary school)	1328	Mean age: 16 years Males 58.9%	Not specified	DUSI-R	Prevalence of substance abuse was 33.9% but substances not specified Substance use associated with psychiatric morbidity, school performance, social competence, peer relations, involvement in recreation, behavior problems (p<0.001 in each case).
Nguchu et al. 2009 [159]	Cross-sectional	Patients with diabetes (Hospital)	400	Mean age: 63.3 years Males 60%	Tobacco smoking	None	Prevalence of tobacco smoking was 8.4%
Peltzer et al. 2009 [180]	Cross-sectional	Students (School)	2758^c^	13–15 years Country specific gender distribution not reported	Alcohol, tobacco, illicit drugs (not specified)	Global School-Based Health Survey questionnaire	Prevalence tobacco use 17.5%, illicit drug use 9.5%, risky drinking 4.7%
Ndetei et al. 2010 [34]	Cross-sectional	Students (Secondary school)	343	Mean age: 16.8 years Males 64.1%	Alcohol, tobacco, cannabis, khat, cocaine, heroin	None	Alcohol, tobacco, khat and cannabis were the most commonly reported substance of use, with user prevalence rates of 5.2%, 3.8%, 3.2%, and 1.7%, respectively.
Tegang et al. 2010 [195]	Cross-sectional	FSWs (Community)	297	Median age 25 (IQR 21–29) All female	Tobacco smoking, khat, alcohol, heroin	None	Lifetime prevalence:91% for alcohol, 71% for *khat*, 34% for cannabis, and 6% for heroin, cocaine, glue or petrol. Lifetime prevalence of at least one substance was 96%, at least two substances 80% Lifetime use khat associated with age at first paid sex of <20 years (p<0.01); lifetime use tobacco associated with engagement in sex work of >5years (p<0.05); lifetime use heroin/cocaine/ glue/petrol associated with sex with 2 or more partners (p<0.005).
Atwoli et al. 2011 [72]	Cross-sectional	Students (University)	500	Mean age: 22.9 (SD2.5) Males 52.2%	Alcohol, tobacco	WHO Model Core Questionnaire	Alcohol use: lifetime prevalence was 51.9%; Current prevalence was 50.7%; Among those using alcohol, 50.4% used 5 or more drinks per day, on 1 or 2 days and 9.2% used for3 or more days. Lifetime tobacco smoking was 42.8%; cannabis (2%), cocaine (0.6%). Tobacco use higher among males compared to females (p < 0.05). 75.1% introduced to substances by a friend Reasons for use: to relax (62.2%) or relieve stress (60.8%).
Kinoti et al. 2011 [114]	Cross-sectional	Adults (Community)	217	Mean age: 34.2 years males 70.5%	Alcohol, khat	None	Prevalence of use for bottled beer: 64.8%; local brew– 41.6%; khat chewing– 41.6%; cannabis -13.7% Males significantly more likely to use bottled beer (p<0.01) and local brew (p<0.01) and khat (p<0.01) Unemployment associated with use of bottled beer (p<0.05) and local brew (p<0.01) and khat (p<0.01)
Luchters et al. 2011 [129]	Cross-sectional	MSW (Community)	442	Mean age: 24.6 (SD 5.2) All males	Alcohol and others (Khat, rohypnol, heroin or cocaine)	AUDIT	Alcohol: overall prevalence of use 70%; 35% of participants who drink had hazardous drinking, 15% harmful drinking and 21% alcohol dependence. Binge drinking prevalence of 38.9% Prevalence of other substances (khat 75.5%, cocaine/heroin 7.7%, rohypnol 14.9%) Alcohol dependence was associated with inconsistent condom use (AOR = 2.5, 95%CI = 1.3–4.6), penile or anal discharge (AOR = 1.9, 95% CI = 1.0–3.8), and two-fold higher odds of sexual violence (AOR = 2.0, 95%CI = 0.9–4.9).
Muture et al. 2011 [148]	Case-control	Cases were patients on treatment for tuberculosis (Hospital)	1978 cases and 945 controls	Mean age/age range: mean 31.2 years for cases and 29.5 years for controls Males 59.4% in cases and 53% of controls	Alcohol	None	Alcohol abuse was found to be a predictive factor for defaulting from TB treatment (OR 4.97; CI 1.56–15.9).
Ndugwa et al. 2011 [156]	Cross-sectional	Adolescents living in an informal settlement (community)	1722	Mean age: 12–19 years Males 47.2%	Alcohol, tobacco, miraa, glue illicit drugs (not specified)	MPBI	Lifetime prevalence of alcohol use was 6.0%; tobacco smoking was 2.6%; other illicit drugs (not specified) 6.8%
Peltzer et al. 2011 [181]	Cross-sectional	Pupils (Primary school)		Mean age/range: 13–15 years Gender distribution: 47.7%	Tobacco smoking	GSHS core questionnaire	Lifetime smoking prior to age 14 years reported by 15.5% (20.1% boys and 10.9% girls) early smoking initiation was among boys associated with ever drunk from alcohol use (OR = 4.73, p = 0.001), ever used drugs (OR = 2.36, p = 0.04) and ever had sex (OR = 1.63, p = 0.04). Among girls, it was associated with higher education (OR = 5.77, p = 0.001), ever drunk from alcohol use (OR = 4.76, p = 0.002), parental or guardian tobacco use (OR = 2.83, p = 0.001) and suicide ideation (OR = 2.05, p = 0.02)
Embleton et al. 2012 [85]	Cross-sectional	Children living in the streets	146	Age range: 10–19 Males 78%	Alcohol, glue, tobacco, cannabis, khat, prescription medication, petrol	None	Lifetime substance use was 74%, current substance use was 62% Lifetime and current prevalence for specific substances respectively was: glue 67%, 58%; alcohol 47%, 16%; tobacco 45% 21%; khat 33%,7%; cannabis 29%,11%; petrol 24%,5%; and pharmaceuticals 8%,<1% Factors associated with having any lifetime drug use were increasing age (adjusted odds ratio [AOR] = 1.47, 95% CI = 1.15–1.87), having a family member who used alcohol, tobacco, or other drugs (AOR = 3.43, 95% CI = 1.15–10.21), staying in a communally rented shelter (AOR = 3.64, 95% CI = 1.13–11.73), and being street-involved for greater than 2 years (AOR = 3.69, 95% CI = 1.22–11.18).
Kuria et al. 2012 [120]	Cross-sectional	Persons with alcohol use disorder in an informal settlement (community)	188	Mean age: 31.9 years Male 91.5%	Alcohol	CIDI, ASSIST and AUDIT	Tobacco—50% of the participants Cannabis—21.3% There was a statistically significant association (P value 0.002) between depression and the level of alcohol dependence at intake. And at 6 months
Menach et al. 2012 [136]	Case-control	Cases were adults with laryngeal cancer (Hospital)	100 (50 cases, 50 controls)	Mean age: 61years in cases and 63years in control group 96% males	Alcohol, tobacco	None	Being a current smoker increased laryngeal cancer risk with an odds ratio (OR) of 30.4 (*P* < 0.0001; 95% CI: 8.2–112.2).
Ndetei et al. 2012 [155]	Cross-sectional	Psychiatric Patients (Hospital)	691	Schizoaffective disorder: Mean age 33.1 years; Males 52.2% Schizophrenia mean age: 33.5 years; Males:62.9% Mood disorders: mean age 33.2 years; Males: 58.4%	Alcohol, drugs (not specified)	SCID-I for DSM IV	Comorbidity with alcohol dependence disorder was more common in schizoaffective disorder than with schizophrenia (p = 0.008)
Ayah et al. 2013 [73]	Cross-sectional	Adults living in informal settlements (community)	2061	Mean age 33.4 years Males 50.9%	Alcohol, tobacco	WHO STEPS survey instrument	Tobacco use Current smoking 13.1% of whom 84.8% were daily smokers. The mean age of smoking commencement and duration of smoking was 19.7 years and 16.5 years Respectively Alcohol use Lifetime prevalence 30%, of whom 74.9% used in past 12 months and 62.2% in the previous 30 days Daily use was 19.7% and use 1–6 days per week among 43.4% Duration of smoking (p = 0.001) and number of pack years(p = 0.049) associated with diagnosis of diabetes
Embleton et al. 2013 [86]	Mixed-methods (cross-sectional and qualitative)	Children living in the streets	146	Age range: 10–19 years males 85%	Alcohol, glue, tobacco, khat, cannabis, petrol, prescription medication	None	Prevalence of substance use was as follows: glue 67%; alcohol 47%; tobaccos 45%; khat 33%; cannabis 29%; petrol 24%; and pharmaceuticals 8%;
khasakala et al. 2013 [108]	Cross-sectional	Youth attending an out-patient clinic (Hospital)	250	Mean age: 16.92 years Males 59.1	Alcohol, other substances (not specified)	MINI (DSM IV)	Any drug use prevalence was 62.4% Alcohol abuse prevalence was 47.8% associations between major depressive disorders and any drug abuse (OR = 3.40, 95% CI 2.01 to 5.76, p < 0.001), or alcohol use (OR = 3.29, 95% CI 1.94 to 5.57, p < 0.001),
khasakala et al. 2013 [109]	Cross-sectional	Youth and biological parents attending a youth clinic (Hospital)	678 (250 youth, 226 biological mothers, 202 biological fathers)	Mean age youth 16.92years males 59.1% (youth)	Alcohol, other substances (not specified)	MINI (DSM IV)	Alcohol use—46.8% of youth, 1.2% mothers and 39.2% of fathers Multiple drug use identified in 9% of youth Significant statistical association between alcohol abuse (p <0.001), substance abuse (p < 0.001) and suicidal behaviour in youths.
Kinaynjui & Atwoli 2013 [115]	Cross-sectional	Inmates (Prison)	395	Mean age: 33.3 years Males 68.6%	Alcohol, tobacco, cannabis, amphetamines, inhalants, sedatives, tranquillizers, cocaine, heroin.	WHO Model Core questionnaire	Lifetime prevalence of any substance use was 66.1% Lifetime prevalence: alcohol 65.1%, tobacco use 32.7%, tobacco chewing 22.5% admitted to chewing tobacco, cannabis 21%, amphetamines (9.4%), volatile inhalants (9.1%), sedatives (3.8%), tranquillizers (2.3%), cocaine (2.3%), and heroin (1.3%). Substance use associated with male gender (p<0.001), urban residence (p<0.001).
Lo et al. 2013 [128]	Cross-sectional	Adults (Community)	72292	Modal age group: 18–29 years males 43.1%	Alcohol, tobacco	None	Prevalence of ever smoking was 11.2% and of ever drinking, 20.7%. Percentage of current smokers rose with the number of drinking days in a month (P < 0.0001). Tobacco and alcohol use increased with decreasing socio-economic status and amongst women in the oldest age group (P < 0.0001).
Mundan et al. 2013 [142]	Cross-sectional	Military personnel attending a clinic (Hospital)	340	Mean age: hypertensives 45.1(SD 7.7); normotensive 40.8 (SD 7.3) Males 91.6%	Alcohol, tobacco	None	Alcohol use in 63% of hypertensive patients and 52.07% of normotensive patients Smoking prevalence was 11% among those with hypertension and 4.2% among normotensives. hypertension associated with daily (*P* < 0.01) and 1–3 times per week (*P* < 0.05), consumption of alcohol daily Smoking duration is significantly (*P* < 0.05) longer among participants with hypertension compared to normotensives.
Njoroge et al. 2017 [162]	Cross-sectional study	ART-naïve HIV-1 sero-discordant couples attending a clinic (Hospital)	196 (99 HIV-infected and 97 HIV-uninfected)	Median age 32 years Males 50%	Tobacco, smoking	None	Smoking: prevalence among those HIV positive was 10% current and past was 22%; among those HIV negative was 11% current and 9% past
Njuguna et al. 2013 [25]	Cross-sectional	Adults (Community)	75	Mean age: 28.3 Males 100%	Khat	None	Overall prevalence of khat use was 68% Khat use was associated with being employed (OR = 2.8, 95% CI 1.03–7.6) Reasons for starting to chew khat included peer influence (40.4%), idleness (23.1%), easy access to khat (19.2%), and curiosity (17.3%)
Okal et al. 2013 [164]	A combination of ‘multiplier method’, the ‘Wisdom of the Crowds’ (WOTC) method and a published literature review.	MSM, PWID, FSWs (Community)	Not reported	Age and gender distribution data not given	Injection drugs (not specified)	None	Approximately 6107 IDU and (plausibly 5031–10 937) IDU living in Nairobi.
Patel et al. 2013 [179]	Case-control	Cases were adults with oesophageal cancer (Hospital)	159 cases and 159 controls	Mean age for males 56.09 years and females was 54.5 years Males 57.9%	Alcohol, snuff, tobacco smoking	None	Smoking, use of snuff and alcohol were associated with increased risk of esophageal cancer (OR = 2.51, 4.74 and 2.64 respectively)
Ploubidis et al. 2013 [184]	cross-sectional	Adults (Community)	4314	Mean age: 60.8 years Males 49.2%%	Alcohol, tobacco smoking	None	Prevalence of alcohol was 17.7% and smoking prevalence was 6.8%
Balogun et al., 2014 [75]	cross-sectional	Pupils (Primary School)	3666	Age range: 13–15 years Males 49.1%	Alcohol	None	Past 30-day alcohol use was 17.9% Lifetime drunkenness was 22.5% Past 30-day alcohol use associated with increased odd sleeplessness; Lifetime drunkenness associated with both depression and sleeplessness
Bengston et al., 2014 [77]	Cross-sectional	FSWs (Community)	818	Age distribution: 30% aged 18–23 All female	Alcohol	AUDIT	Prevalence of hazardous drinking was 64.6%; harmful drinking was 35.5% Higher levels alcohol consumption associated with having never tested for HIV (PR 1.60; 95% CI: 1.07, 2.40).
Chersich et al., 2014 [80]	Cross-sectional	FSWS (Community)	602	Mean age: 25.1 years Female 100%	Alcohol	AUDIT	Prevalence of hazardous drinking was 17.3% and harmful drinking was 9.3% Harmful drinking associated with increased odds sexual (95% CI adjusted odds ratio [AOR] = 1.9–8.9) and physical violence (95% CI AOR = 3.9–18.0); while hazardous drinkers had 3.1-fold higher physical violence (95% CI AOR = 1.7–5.6).
De Menil et al., 2014 [83]	Cross-sectional	Psychiatric patients (Hospital)	455	Mean age/range: 36.3 years Gender distribution: males 66.4	Alcohol, other substances (not specified)	None	Prevalence of alcohol use disorder was 21.2% and other drug use was 10.4%
Joshi et al., 2014 [98]	cross-sectional	Adults living in informal settlements (Community)	2061	Mean age: 33.4 (SD 11.6) years Males 50.9%	Alcohol, tobacco	WHO STEPS	Alcohol use: 30.1% reported lifetime alcohol use; 81% alcohol use in past 12 months; 76.8% reported using alcohol in the past 30 days; harmful use by 52% Tobacco: 13.1% reported current smoking (84% of whom used daily) Current smoking (p = 0.018), years of smoking (p = 0.001) associated with having hypertension
Medley et al., 2014 [135]	Cross-sectional	PLHIV (Hospital)	1156	Mean age: 37.2 Gender distribution not reported	Alcohol	None	Overall, 14.6% of participants reported alcohol use in the past 6 months; 8.8% were categorized as non-harmful drinkers and 5.9% as harmful/likely dependent drinkers. Binge drinking reported in 5.4%
Othieno et al., 2014 [169]	Cross-sectional	Students (University)	923	Mean age: age 23 (SD4.0) males 56.9%	Alcohol, tobacco	None	Students who used tobacco (p = 0.0001) and engaged in binge drinking (p = 0.0029) were more likely to be depressed
Pack et al., 2014 [174]	Cross-sectional	FSW (Community)	619	18 years and older Female 100%	Alcohol	AUDIT Tool not specified for other drug use	Hazardous alcohol use 36.0%; harmful alcohol use 64.0%; other drug use 34.1%
Were et al., 2014 [199]	Cross-sectional	PWID (Community)	61	Age range: 29–33 years Gender distribution: not reported	Brown sugar, rohypnol, khat, tobacco, cocktail, alcohol, injection drugs (heroin, diazepam)	None	Prevalence of substance use was as follows: 43%, brown sugar 16%, rohypnol 61%, tobacco 61%, khat 26%, cocktail 39%, alcohol 52%; injection drugs heroin 100%, diazepam 18%
Widmann et al., 2014 [201]	Case-control	Cases were male khat chewers (Community)	48 (cases = 33, controls = 15)	Mean age: 34 years for cases, 35.1 for controls Males 100%	Alcohol, khat, tobacco, tranquilizers	MINI	Khat chewers experienced more traumatic event types than non-chewers (*p* = 0.007), more PTSD symptoms than non-chewers (*p* = 0.002) and more psychotic symptoms (p = 0.044).
Goldblatt et al., 2015 [91]	Cross-sectional	Children living in the streets	296	Age range: 13-21years All males	Alcohol, tobacco, khat, glue, fuel	None	Weekly alcohol use reported by 49%;93% reported weekly tobacco use; and 39% reported weekly Cannabis use; 46% reported lifetime use of glue; 8% reported lifetime inhalation of fuel
Hulzelbosch et al., 2015 [96]	Cross-sectional	Persons with hypertension in an informal settlement (Community)	440	Age: 35 years and above males 42%	Alcohol, tobacco, khat, glue, fuel	WHO STEPS survey instrument	Tobacco use: current 8.4%, former 11.8% Alcohol use: low 84.8%, moderate 6.8%, high 8.4%
Kurth et al., 2015 [121]	Cross-sectional	PWID (Community)	1785	Mean age 31.7 years in Coast and 30.4 in Nairobi Males 82.4–89.0%	Injection drugs (heroin)	None	93% injected heroin in the past 30 days.
Lukandu et al., 2015 [130]	Case-control	Cases were dental patients (Hospital)	42 (34 cases, 8 controls)	mean age 28.9 years all males	Alcohol, khat, tobacco,	None	Oral epithelium thicker in khat chewers compared non-chewers (p<0.05);
Maina et al., 2015 [132]	Cross-sectional	PLHIV (Hospital)	200	Modal age group 34–41 years (27.4%) males 49.7%	Alcohol, tobacco, cocaine, amphetamines, inhalants, sedatives, opioids, hallucinogens, others (not specified)	ASSIST, ASI	Lifetime prevalence of any substance use was 63.1%; alcohol 94.4%; tobacco 49.7%; cocaine 6.7%; amphetamine type stimulants 19.6%; inhalants 3.4%; sedatives 1.7%; opioids 1.1%; hallucinogens 6.6%; others 4.2% 50.3% wrongly identified the alcohol use vignette problem as stress
Micheni et al., 2015 [33]	Cohort	MSM and FSW (Community)	1425	Median age was 25 for MSM and 26 for FSW Males 50.9%	Alcohol, injection drugs (not specified)	None	Recent alcohol use was associated with reporting of all forms of assault by MSM [(AOR) 1.8, CI 0.9–3.5] and FSW (AOR 4.4, CI 1.41–14.0),
Muraguri et al., 2015 [144]	Cross-sectional	MSM (Community)	563	MSM who did not sell sex: 30% in the 35 and older age group; MSM who sell sex: 30.8% in the 25–29 age group Males 100%	Alcohol, illicit drugs (not specified)	AUDIT for alcohol use; tool not specified for illicit substances	62.9% of MSM who did not sell sex had used illicit drugs in the past 12 months while those who sold sex were 78.7%. Possible alcohol dependence was 21.4% among those who did not sell sex while those who sold sex were 33%.
Olack et al., 2015 [165]	Cross-sectional	Adults living in informal settlements (Community)	1528	Mean age: 46.7 years Males 42%	Alcohol, tobacco smoking	WHO STEPS survey questionnaire	Prevalence of smoking: Current smokers 8.5% and past Smokers 5.1%; Alcohol: Ever Consumed was 30.4%; In the past 12 months was 17% and In the past 30 days was 6.5%
Onsomu et al., 2015 [167]	Cross-sectional	Adult women (Community)	2227	Age range not reported Females 100%	Alcohol use in husband	None	385 of women reported that husband uses alcohol
Othieno et al., 2015 [170]	Cross-sectional	Students (University)	923	Mean age: age 23 (SD4.0) Males 56.9%	Alcohol, tobacco	None	Alcohol use (p<0.001), binge drinking (p<0.01), tobacco use (p<0.001), were significantly associated with increased odds of having multiple sexual partners.
Othieno et al., 2015b [171]	Cross-sectional	Students (University)	923	Mean age: age 23 (SD4.0) Males 56.9%	Alcohol, tobacco	None	Prevalence of binge drinking was 38.85%; Tobacco use prevalence not reported Binge drinking and tobacco use were significantly associated with injury in the last 12 months (AOR 5.87 and 4.02, p<0.05, respectively)
Secor et al., 2015 [189]	Cross-sectional	MSM Community)	112	Median age: 26 years Males 100%	Alcohol, other drugs (not specified)	AUDIT, DAST	Prevalence of hazardous or harmful alcohol use was 45%; prevalence harmful use of other drugs 59.8% Alcohol abuse associated with higher PHQ-9 scores (p = 0.02).
Syvertsen et al., 2015 [190]	Cross-sectional	PWID (Community)	151	Mean age: 28.8 (SD 6.2) years Males 84%	Alcohol, cannabis, prescription pills, cocaine, heroin	None	Prevalence of substance use was: Alcohol at 92.4%; cannabis at 67.6%; prescription pills at 21.2%; cocaine injection at 76.2%; Heroin injection at 29.1% The mean years of injecting was 6.2;
Tun et al., 2015 [197]	Cross-sectional	PWID (Community)	269	Median age 31 years Males 92.5%	Injection drugs, cannabis, khat, cocaine, tranquilizers	None	Past month injecting drug use (white heroin-97%; other 3%); past month use: cannabis -66.5%; Khat- 10.8%; cocaine 3.7%; tranquilizers- 58.0% HIV infection was associated with having first injected drugs 5 or more years ago (aOR, 4.3, p = 0.002), and ever having practiced receptive syringe sharing (aOR, 6.2; p = 0.001)
Winston et al., 2015 [204]	Cross-sectional	Children living in the streets	200	Mean age: 16 years Males 59%	Alcohol and other drugs (not specified)	None	Prevalence of alcohol use was 45.5%; and any drug use was 77.0% Among females, those with HIV infection more frequently reported drug use (91.7% vs 56.5%, p = 0.02),
Mokaya et al., 2016 [140]	Cross-sectional	Health care workers (Hospital)	206	Mean age: 35.3 years (SD 10.1) Males 36.9%	Alcohol, tobacco, sedatives, cocaine, amphetamine-like stimulants, hallucinogens, inhalants,	ASSIST	Lifetime use was 35.8% for alcohol, 23.5% for tobacco, 9.3% for sedatives, 8.8% for cocaine, 6.4% for amphetamine-like stimulants, 5.4% for hallucinogens, 3.4% for inhalants, and 3.9% for opioids Being male associated with lifetime tobacco (p<0.01), alcohol (p<0.01) and cannabis (p<0.01) use.
Papas et al., 2016 [176]	Mixed methods	PLHIV (Hospital)	127	Median age 37.0 years (IQR 32.0–43.0) Males 48.2%	Alcohol, kuber, tobacco, cannabis, khat,	AUDIT-C	Prevalence of substance use was as follows: alcohol: ≥6 drinks per occasion at least monthly in the past year was 51.2%; Past 30 days other drug use: Tobacco—25.2%; cannabis—3.9%; khat- 8.7%; kuber -10.2% No agreement between self-reported alcohol use and PETH
White et al., 2016 [200]	Cohort	FSW (community)	405	Modal age group 40–49 years All female	Alcohol	AUDIT	Hazardous/harmful alcohol use significantly associated with a lower likelihood of self-reported sexual abstinence (aRR 0.58; 95% CI 0.45–0.74)
Wilson et al., 2016 [203]	Cross-sectional	FSWs who are PLHIV (hospital)	357	Age range: 20–61 years Females 100%	alcohol	AUDIT	Any alcohol use was 48.7%; Among those using 59.1% had drinking behaviour consistent with minimal alcohol use problems, 32.8% moderate problems and 8% had severe alcohol problems or possible alcohol use disorder Women with severe alcohol problems (adjusted odds ratio 4.39, 1.16–16.61) were significantly more likely to report recent intimate partner violence.
Embleton et al., 2017 [87]	Cross-sectional	Orphaned and separated children (Community, charitable institutions)	1365	Mean age 13.9 years Males 52%	Alcohol, drugs (not specified)	None	Prevalence of alcohol and drug use was 8.9%
Goodman et al., 2017 [92]	Cross-sectional	Mothers (Community)	1976	Mean age: 38.2 years Females 100%	Alcohol	None	7.95% reported any alcohol consumption and 5% reported weekly alcohol consumption Physical abuse (OR) = 2; 95% CI: (1–4.2)), emotional neglect (OR = 3.18; 95% CI: (1.47–6.91), and living with someone with a mental illness or depression (OR = 2.14; 95% CI: (1.05–4.34)) during the first 18 years of life significantly increased the odds of reporting weekly alcohol consumption.
Jenkins et al., 2017 [97]	Cross-sectional	Adults (Community)	1147	Age range: 18–60 years Gender distribution: not reported	Alcohol	AUDIT	Lifetime alcohol use was 14.5% for men and 6.8% for women; Hazardous drinking was 9.5% of men and 2.9% of women. Risk of hazardous drinking was increased in men (OR 0.3, C.I. = 0.17 to 0.58 p < 0.001), people living in larger households (OR 1.8, C.I. = 1.09 to 2.97, p = 0.021), people who were single (OR 1.7, C.I. = 0.92 to 3.04, p = 0.093), and those who are self-employed (OR 1.8, C.I. = 1.04 to 2.99, p = 0.036).
Kamau et al., 2017 [101]	Cross-sectional	Children and adolescents attending a psychiatry out-patient clinic (Hospital)	166	mean age: 13.6 (SD 4.16) years males 56%	Alcohol, tobacco, stimulants, cocaine	KSADS and DSM-IV Criteria	Substance use disorder (30.1%) most prevalent presentation. Prevalence tobacco use -6.0%; Alcohol abuse & dependence—7.2%; cannabis abuse and dependence—14.5%; Stimulant abuse 1.8%; cocaine dependence 0.6%
Kimando et al., 2017 [111]	Cross-sectional	Patients with diabetes (Hospital)	385	Mean age 63.3 years Males 34.5%	Tobacco smoking, alcohol	None	Tobacco smoking was 23.6%; alcohol prevalence was 26.5% Alcohol influences cardiovascular risk factor control (p<0.001)
Kunzweiler et al., 2017 [118]	Cohort	MSM (Community)	711	Median age (IQR): 24[21–28]	Alcohol	AUDIT-C	Previously diagnosed HIV-positive and out-of-care status was more likely than HIV-negative status among men who did not report harmful alcohol use (p = 0.28)
Kwobah et al., 2017 [126]	Cross-sectional	Adults (Community)	420	Median age 34 years, IQR 27–46 Males 48.6%	Alcohol and other substances (not specified)	MINI-7	Alcohol/ Substance Use Disorders (11.7%). Other substances were not specified.
Muthumbi et al., 2017 [146]	Case-control	Cases were patients with pneumonia (Hospital)	281 cases and 1202 controls	Among the 281 cases: 63% were male and 23% aged 15–24 years.	Alcohol, tobacco, snuff, khat	None	Pneumonia associated current smoking (2.19, 95% CI 1.39–3.70), use of khat (OR 3.44, 95% CI 1.72–7.15), use of snuff (OR 2.67, 95% CI 1.35–5.49)
Papas et al., 2017 [177]	Cross-sectional	PLHIV with active alcohol use (Hospital)	614	Mean age: Male 40.3, Female 37.5 Male 48.5%	Alcohol	AUDIT-C	Alcohol use not associated with physical and sexual violence among both men (p = 0.434) and women (p = 0.449)
Roth et al., 2017 [185]	Cross-sectional	Adult males who use alcohol (community)	220	Mean age: 35.2 years all males	Alcohol	None	Drinking alcohol with FSWs associated with ever having commercial sex (p<0.001), fighting with FSWs (p<0.01), being physically hurt by FSWs (p<0.01), physically hurting FSWs (p<0.001), being robbed by FSWs (p<0.001)
Takahashi et al., 2017 [192]	Cross-sectional	Adults (Community)	478	Mean age: 41(SD 14) Males: females 41.4%	Alcohol, tobacco	AUDIT	Alcohol: prevalence of current drinking was 31.7% and hazardous drinking was 28.7% Tobacco use prevalence was 14.4% Current (p<0.001) and hazardous alcohol use (p<0.001) associated with being male
Tsuei et al.,2017 [196]	Cross-sectional	Health care workers (Hospital)	206	Mean age: 35.0 years (SD 10.1) Males 37.2%	Alcohol, tobacco	ASSIST	Prevalence moderate risk alcohol use (3.0%); moderate and high risk tobacco use (11.8% and 0.5%) respectively; moderate risk cannabis use (3.4%) Self-efficacy for SUD was lower in those practicing in public facilities and perceiving a need for AUD training; while higher self-efficacy correlated with a higher proportion of patients with AUD in one’s setting, access to mental health worker support, cannabis use at a moderate risk level, and belief that AUD is manageable in outpatient settings.
Asiki et al., 2018 [70]	Cross-sectional	Adults living in informal settlements (community)	1942	Mean age of women was 48.3 (SD 5.30), and of men was 48.8(SD 5.6) Males 45.6%	alcohol, tobacco,	CAGE	BMI among men negatively associated with current tobacco smoking,
Budambula et al., 2018 [78]	Cross-sectional	PWID use, non-injecting drug users, non-drug users, with and without HIV (Community)	451	Among PWID (HIV positive): Median age 30.6; Males 45.2% Among PWID (HIV negative): Median age 26.8; Males 64.1%	Injection drugs, non-injection drugs (not specified)	None	Occurrence of early age sexual debut, >1 sexual partners, unprotected sex and history of STIs (all p<0.0001) was significantly higher in HIV-infected PWID use than in non-injection drug users and non-drug users Frequency of bisexuality, homosexuality, sex for police protection, sex for drugs was (all p<0.0001) significantly higher in HIV-infected PWIDs as compared to non-injection drug users and non-drug users
Cagle et al.,2018 [79]	Cohort	PLHIVV (Hospital)	854	Age: 15 years and above 61% females	Alcohol	AUDIT	CD4 count increase was associated with alcohol use (p = 0.051) following ART initiation in ART naïve patients
Gathecha et al., 2018 [88]	Cross-sectional	Adults (community)	4484	Age range 18-69years Males 60.3%	Alcohol, tobacco	WHO STEPS survey questionnaire	Smokers (p = 0.001) were significantly more likely to be injured in a road traffic crash. Heavy episodic drinking (p = 0.001) and smoking (p < 0.05) were associated with increased likelihood of occurrence of a violent injury.
Kaduka et al., 2018 [100]	Cohort	Patients with stroke (Hospital)	691	Median age 60 years Males 42.4%	Tobacco, cocaine	WHO STEPS survey	Tobacco smoking risk factor for ischemic stroke (*p* < 0.001).
Kendagor et al., 2018 [107]	Cross-sectional	Adults (Community)	4203	Age range: 18–69 years Males 60%	Alcohol, tobacco	WHO STEPS survey questionnaire	12.7% reported heavy episodic drinking, Respondents who were separated had three times higher odds of HED compared to married counterparts (OR 2.7, 95% CI 1.3–5.7). Tobacco consumption was associated with higher odds of HED (unadjusted OR 6.9, 95% CI 4.4–10.8)
Kiburi et al., 2018 [110]	Cross-sectional	Psychiatric in-patients (Hospital)	134	Modal age group 31–40 Males 88.1%	Alcohol, tobacco, opioids, cocaine, amphetamines, inhalants, sedatives, khat	ASSIST	Lifetime: prevalence tobacco 84.3%, alcohol 91.8%, cannabis 64.2%, cocaine 5.2%, amphetamine 3%, inhalants 5.2%, sedatives 22.4%, hallucinogens 3.7%, opioids 8.2%, khat 55.2%; 90% had poly-substance use Emotional abuse significantly predicted tobacco (A.O.R = 5.3 (1.2–23.9) and sedative (A.O.R = 4.1 (1.2–14.2) use. Childhood exposure to physical abuse was associated with cannabis use [A.O.R = 2.9 (1.0–7.9)].
Kimbui et al., 2018 [113]	Cross-sectional	Pregnant adolescents (Hospital)	212	Mean age: 17.3 years Males 88.1%	alcohol	AUDIT	43.9% had used alcohol Depression was associated with ever use of alcohol (p = 0.038), and alcohol dependence (p = 0.004)
Korhonen et al., 2018 [117]	Cross-sectional	Gay, bisexual and other MSM (Community)	1476	Median age (IQR 22–29), Males 100%	Alcohol, other substances (not specified)	AUDIT, DAST	Prevalence for hazardous alcohol use was 44% and for problematic substance use was 51% Transactional sex was associated with hazardous alcohol use [adjusted prevalence ratio (aPR) 1.34, 95% confidence interval (CI) 1.12–1.60]. Childhood abuse and recent trauma were associated with hazardous alcohol use (aPR 1.36, 95% CI 1.10–1.68 and aPR 1.60, 95% CI 1.33–1.93, respectively), and problematic substance use (aPR 1.32, 95% CI 1.09–1.60 and aPR 1.35, 95% CI 1.14–1.59, respectively).
Kunzweiler et al., 2018 [119]	Cross-sectional	MSM (Community)	711	Median age 24 years Males 100%	Alcohol, other substances (not specified)	AUDIT, DAST	Prevalence of harmful alcohol use was 50.1% and prevalence of moderate substance abuse was 23.8% Depressive symptoms were associated with harmful alcohol use (p<0.01) and moderate substance abuse (p = 0.02)
Magati et al., 2018 [131]	Cross-sectional	Adults & adolescents (community)	43898	Age range: 15–54 rears females 70.8%	Tobacco	None	Overall smoking and smokeless tobacco prevalence rate was 17.3% and 3.10% respectively among men. Lower rates in women with smoking and smokeless tobacco prevalence at 0.18% and 0.93%
Mannik et al., 2018 [133]	Cross-sectional	Adults (Community)	2865	Median age 50 years Males 45%	Tobacco	None	The point prevalence of tobacco use was 22%.
Mburu et al., 2018 [134]	Cohort	Patients with tuberculosis (Hospital)	347	Median age 31years Males 71.8%	Alcohol, tobacco	None	Alcohol use and smoking were associated with DM among TB patients (p<0.200) Number of cigarettes smoked per day and significant risk factors of developing DM among TB patients (p = 0.045)
Mkuu et al., 2018 [138]	Cross-sectional	Adults (Community)	718	Mean age 36.6 years Males 86%	Alcohol, tobacco smoking	AUDIT	An average of 2.5 drinking events and 4.3 binge-drinking occasions per month. 37% consumed unrecorded alcohol. Those who completed primary education or above less likely to report consuming unrecorded alcohol compared to those with incomplete primary education or lower, (OR = 0.22, 95% CI: 0.12–0.43). Compared to poorest and poor respondents, those identifying as middle class or above were less likely to consume unrecorded alcohol (OR = 0.47, 95% CI: 0.29–.78). Current smokers (OR = 2.19, 95% CI: 1.34–3.60) and those with higher binge drinking occasions in the past month (OR = 1.03, 95% CI: 1.004–1.07) were significantly more likely to consume unrecorded alcohol.
Mohammed et al., 2018 [139]	Cross-sectional	Adults (Community)	4484	Modal age group 18–29 (46%); Gender distribution: not reported	Alcohol, tobacco	WHO STEPS survey questionnaire	Prevalence of current tobacco use was 13.4% and harmful alcohol use was 14.4%. Harmful alcohol use was associated with hypertension (p < 0.001).
Ng’ang’a et al., 2018 [157]	Case-control	Cases were women screened for cervical cancer (Community)	1180 (194 cases, 986 controls)	Age range: 30–49 years Females 100%	alcohol, tobacco	None	Those with binge drinking more likely to be screened for cervical cancer [OR 5.94, 95%CI 1.52–23.15) p = 0.010]
Ngaruiya et al., 2018 [158]	Cross-sectional	Adults (Community)	4484	Age range: 18–69 years males 48.7%	Alcohol, tobacco	WHO STEPS survey questionnaire	Prevalence of tobacco use: current use was 13.5%; Lifetime alcohol use was 43.1% Men had nearly seven times higher odds of being tobacco users as compared to women (OR 7.63, 95% CI 5.63–10.33). current tobacco use associated with ever use alcohol (p<0.001)
Oyaro et al., 2018 [173]	Cross-sectional	PWID (Community)	673	Majority between 20–34 years Males 93%	Injection drugs (not specified)	None	IDU was positively associated with HCV (aOR = 5.37, 95% CI:2.61–11.06; p < 0.001)
Tang et al., 2018 [194]	Cross-sectional	Adult men (Community)	12815	Mean age: 30 (SD 10.9) Males 100%	Tobacco	None	Trends in tobacco use: the rates declined from 22.9% in 2003 to 18.8% in 2008–2009 and 17% in 2014.
Wekesah et al., 2018 [198]	Cross-sectional	Adults (community)	4066	Age: 18 years and above Male: 48.6%	Alcohol, tobacco	WHO STEPS survey questionnaire	Prevalence of smoking was 10.2% (17.9% males, 2.9% of females) Prevalence of harmful alcohol use 13.8% (24.5% of males and 3.7% of females)
Akiyama et al., 2019 [68]	Cross-sectional	PWID and illicit drug use (NSP sites within the community)	2188	Median age (IQR): 32 years (28–36) Males 91%	Injection drugs, illicit drugs (not specified)	None	Median (1QR) age at first injection 27 years (24–31), Median (1QR) number of injections per day in the past month: 2 (1–3); Median (1QR) years injecting 3(2–6) Needle sharing at last injection: receptive (3%); distributive (3%) More years of injecting and more injections in the past month was associated with increased odds of HIV–HCV co-infection (p>0.0001 in both cases)
Anundo 2019 [69]	Cross-sectional	Female PWID (Community)	149	Age range: 26–40 years Females 100%	Alcohol, tobacco, khat, heroin amphetamines, cocaine, hallucinogens, sedatives.	ASSIST	The substance specific risk scores for frequently used substances were as follows: heroin 38, tobacco 37, alcohol 35, khat 28, rohypnol 1, cocaine 1
Gitatui et al. 2019 [24]	Cross-sectional	Adults living in informal settlements (Community)	215	Age: above 18 years Males 80%	Alcohol	None	Alcohol use reported on average 4.15 ± 2.8 (Mean ± SD) days per week. Respondents who consumed more than three drinks were more likely (p < 0.05) to be older (OR = 5.8, 95% CI:2.3–14.2 and OR = 2.6, 95% CI: 1.1–6.4), married (OR = 8.3, 95% CI: 3.3–21.1), separated/divorced/widowed (OR = 2.8, 95% CI: 1.3–6.5), had attained post primary education (OR = 2.1, 05% CI: 1.1–3.8), and of income above 50 USD (OR = 5.8, 95% CI: 2.5–13.8 and OR = 8.8, 95% CI: 3.1–25.5)
Haregu et al. 2019 [95]	Cross-sectional	Adults living in informal settlements (Community)	5190	Age: 18 years and above Males 53.8%	Alcohol, tobacco	None	lifetime alcohol use was 16.4%; lifetime tobacco use 20.3%
Kaai et al. 2019 [99]	Cross-sectional	Adult smokers (Community)	1103	Age: 18 years and above males 91.5%	Tobacco	None	Quit intentions: 28% had tried to quit in past 12 months; 60.9% had never tried to quit, only 13.8% had ever heard of smoking cessation medication Factors associated with quit intentions: being younger (AOR 3.29 [18–24 years]; AOR 1.98 [25–39 years]), having tried to quit previously (AOR 3.63), perceiving that quitting smoking is beneficial to health (AOR 2.23 [moderately beneficial]; AOR 3.72 [very/extremely beneficial]), worrying about future health consequences of smoking (AOR 3.10 [little/moderately worried]; AOR 4.05 [very worried]), and being low in nicotine dependence (AOR 0.74).
Kamenderi et al. 2019 [102]	Cross-sectional	Students (Secondary schools)	3908	Age data not stated males 60%	Alcohol, khat, prescription medication, tobacco, cannabis, inhalants, heroin, cocaine	None	Lifetime use; alcohol (23.4%), khat (17.0%), prescription medication (16.1%), tobacco (14.5%), cannabis (7.5%), inhalants (2.3%), heroin (1.2%) and cocaine (1.1%);
Kamenderi et al. 2019 [103]	Cross-sectional	Adolescents and adults (Community)	3362 households	Age range: 15–65 years Gender distribution not reported	Alcohol, tobacco, cocaine, heroin, khat,	None	Lifetime prevalence of any substance was 62.5%; alcohol use disorder at 10.4%, tobacco use disorder at 6.8%, khat use disorder at 3.1 and heroin use disorder at 0.8%
Kamenderi et al. 2019 [104]	Cross-sectional	Adults and adolescents (community)	2136 households	Mean age/age range: range 15–65 Males 48.8%	Alcohol, tobacco, khat	DSM V Criteria	Prevalence of multi- substance use was 5.3%; Multiple substance use disorder pattern was as follows; alcohol and tobacco (2.5%); tobacco and khat (0.8%), alcohol and khat (0.7%); alcohol, tobacco and khat (0.5%); alcohol, tobacco, khat and bhang (0.3%), alcohol, khat and bhang (0.2%), alcohol, tobacco and bhang (0.2%); alcohol and bhang (0.1%). Predictors of multiple substance use disorder were: setting (more in urban versus rural area) p = 0.004 and gender (more in females) p = 0001
Kimani et al. 2019 [112]	Cross-sectional	Patients with hypertension (Hospital)	229	Modal age group: <50 years (40.2%) Males 44.5%	Alcohol, tobacco smoking,	None	Prevalence of tobacco smoking 8.3% and alcohol use 13.1% More males reported drinking alcohol and smoking (p<0.001). Higher BPs were observed in smokers and drinkers (p<0.05).
Kisilu et al. 2019 [29]	Cross-sectional	Persons on MMT (MMT clinics)	388	Age distribution not reported Males 93%	Alcohol, tobacco, khat, heroin, benzodiazepine, amphetamines, cocaine, barbiturates.	None	Type of substance first used: Cannabis 35.9%, tobacco 29.1%, alcohol 12%, heroin 11.3%, khat 5.9%, benzodiazepine 3%; glue 1.5%, amphetamines 0.3%, cocaine 0.3% and barbiturates 0.2%.
Kurui & Ogoncho 2019 [122]	Cross-sectional	Students (College)	303	Mean age: 21.96 years Males 49.5%	Alcohol, tobacco, khat, heroin, prescription drugs, emerging drugs (shisha, kuber, shashaman, others not specified)	None	Lifetime use of any substance 52.5%; alcohol 52.5%, Tobacco 12.2%, khat 17.5%, heroin 1.3%, prescription drug 12.5%, emerging drugs 11.2%
Menya et al. 2019 [137]	Case-control	Patients with esophageal cancer (Hospital)	836 (422cases, 414 controls)	Mean age 60 years Males 65% in cases and 61% in control	Alcohol, tobacco	None	For the same amount of ethanol intake, drinkers who had 10 percentage points more ethanol consumed as chang’aa had a 16% (95%CI: 7, 27) higher esophageal squamous cell carcinoma risk.
Mungai & Midigo 2019 [143]	Cross-sectional	Adults (Community)	385	Age range: 18–65 years Males 62.6%	alcohol	AUDIT	Alcohol use: 65% had hazardous or harmful drinking Harmful/hazardous alcohol use associated with having a family member struggling with alcohol use (p<0.001), alcohol being brewed in the home (p<0.001)
Mutiso et al. 2019 [147]	Cross-sectional	Students (Secondary schools)	471	Mean age was 16.33 Males 46.5%	Substances not specified	DUSI-R	No significant differences in the mean scores for substance use problems across all the categories, though the lowest scores were reported among those who had not experienced bullying problems
Mwangi et al. 2019 [149]	Cross-sectional	PWID, women (Community)	306	Mean age 30 years (SD 5.7) Females 100%	Injecting drugs (not specified)	DSM-5 Criteria	88% of participants had severe injecting drug use (IDU) IDU and depression were related to each other (P < 0.05) and each of them with risky sexual behavior (P < 0.05).
Nall et al. 2019 [150)]	Cross-sectional	Youth (Community)	651	Mean age: 16.7years Males 46.5%	Alcohol, tobacco	CRAFFT	A mean score of 1.39 (SD = 0.81) with 30.4% having a score of two or more on CRAFFT, which is the threshold for intervention Substance use predicted intent to test for HIV, (OR = 1.41, p = 0.007.)
Ngure et al. 2019 [160]	Cross-sectional	Students (University)	1438	Age range: 17–33 years Males 53%	Opioids, alcohol, tobacco, shisha, kuber^b^, khat, inhalants, amphetamines, cocaine, hallucinogens, sedatives	ASSIST	Lifetime prevalence of any substance was 48.6% and current prevalence was 37.9% Lifetime prevalence of tobacco -13%, shisha 17.8%, kuber 4.3%, alcohol 43.2%, 14.2%, cocaine 2.7%, amphetamines 1.7%, inhalants 0.8%, sedatives 0.8%, hallucinogens 1.4%, opioids 1.3%, khat 11.5%, muguka 8.1%
Ominde et al. 2019 [35]	Cross-sectional	In-patients with stroke (Hospital)	227	Mean age: 68.8(SD 6.8) Males 37.9%	Alcohol, tobacco	None	Prevalence for alcohol use was 63% and tobacco use was 48%
Ongeri et al. 2019 [166]	Cross-sectional	Adults (Community)	831	Mean age: 30 years Males 47.6%	Khat, tobacco, alcohol, other drugs (not specified)	ASSIST	Khat: lifetime use 44.6%, current use 36.8% Khat use associated with higher odds of reporting strange experiences (OR, 2.45; 95%CI, 1.13–5.34) and experiencing hallucinations (OR, 2.08; 95% C.I, 1.06–4.08) Khat use significantly associated with male sex (p < 0.001), younger age (less than 35 years) (p < 0001), higher level of income (p < 0.001) and comorbid alcohol (p = 0.001) and tobacco use (p < 0.001).
Owuor et al. 2019 [172]	Cross-sectional	Students (University)	404	Mean age: 22.42 (SD 2.45) Males 54.8%	Alcohol, tobacco, sedatives, others (not specified)	ASSIST	Lifetime use of at least one substance was 76% and current use was 46.3%.
Pengpid & Peltzer 2019 [182]	Cross-sectional	Adults (Community)	4469	Median age (38 years) Males 39.7%	Alcohol	WHO STEPS survey questionnaire	12.8% reported past month binge-drinking and 6.7% had hazardous or harmful alcohol use. Current tobacco and khat use was 12.8% and 6.8% respectively Being male (AOR 7.66 [3.92, 14.97]), tobacco use (AOR 6.72 [3.69, 12.2]), and having hypertension (AOR 2.28 [1.49, 3.48]) increased the odds for hazardous or harmful alcohol use.
Woldu et al. 2019 [206]	Cross-sectional	Adults living in informal settlements (community)	413	18 years and older	Alcohol, tobacco, cannabis, khat, cocaine, opioids, sedatives, hallucinogens	ASSIST	Use of any substance in past three months increased the odds of having concurrent sexual relationships (aOR 2.46; 95% CI 1.37–4.42, p < .01).
Kamenderi et al. 2020	Mixed methods (cross-sectional and qualitative)	Pupils (Primary school)	3307	Age distribution not reported Males 51.8%	Alcohol	None	Prevalence of alcohol use was 7.2%
Kurui & Ogoncho 2020 [123]	Cross-sectional	Students (College)	303	Mean age: 21.96 (SD 0.4) years Males 49.5%	Alcohol	None	Prevalence of lifetime alcohol use was 52.5% and current alcohol use was 27.4% Reasons for using alcohol included curiosity 24.1%, fun 12.2%, peer influence 11.6%; Average use- 1 unit 15.2%, 3–4 units 13.2%
Mutai et al. 2020 [39]	Mixed methods (cross-sectional and qualitative)	Adults living in informal settlements (community)	200	Modal age group 18–24 (74%) Males 60%	Alcohol, khat, kuber, heroin, tobacco	None	Prevalence of substance abuse: Cannabis 60%; alcohol 26.5%; khat 6%; kuber, heroin and tobacco 3% each
Ndegwa & Waiyaki 2020 [151]	Cross-sectional	Students (University)	407	Age range: 18–41 Males 41.3%	alcohol, tobacco	ASSIST	Tobacco use was reported by 95.7% (77.9% had low risk, 16.3% moderate risk and 1.5% high risk); Alcohol was reported by 95.7% (77.2% low risk; (16.0%) moderate risk; (2.5%) high risk:
Winter et al. 2020 [205]	Cross-sectional	Adults living in an informal settlement (community)	361	Modal age group: 25–44 years (80%) Female 100%	Alcohol, tobacco	None	Alcohol prevalence was 21.1%, Tobacco prevalence 7.8% Recent psychological IPV was associated with alcohol (OR = 2.6, p<0.05) and tobacco use (OR = 3.8, p<0.05)

^**a**^khat *(catha edulis)* is a plant with stimulant properties and is listed by WHO as a psychoactive substance. Its use is common in East Africa

^b^kuber is a type of smokeless tobacco product.

### Studies evaluating substance use or SUD programs and interventions

#### General description of studies evaluating programs and interventions

A total of eighteen studies evaluated specific interventions or programs for the treatment and prevention of substance use. These were carried out between 2009 and 2020. Eleven studies focused on individual-level interventions, 5 studies evaluated programs, and 2 studies evaluated population-level interventions. The studies used various approaches including randomized control trials (RCT) (n = 7), mixed methods (n = 3), non-concurrent multiple baseline design (n = 1), quasi experimental (n = 1), cross-sectional (n = 2), and qualitative (n = 3). One study employed a combination of qualitative methods and mathematical modeling.

#### Individual-level interventions

*Individual-level interventions for harmful alcohol use*. Nine studies evaluated either feasibility, acceptability, and or efficacy for individual-level interventions for harmful alcohol use [38, 40, 90, 94, 127, 141, 175, 178, 193]. All the interventions were tested among adult populations including persons attending a Voluntary Counseling & Testing (VCT) center (38), PLHIV [40, 175], and adult males and females drawn from the community [94, 141] and FSWs [127, 178].

Two studies evaluated a six session CBT intervention for harmful alcohol use among PLHIV. The intervention was reported as feasible, acceptable [40] and efficacious [175] in reducing alcohol consumption among PLHIV. The intervention was delivered by trained lay providers.

Giusto et al [90] evaluated the preliminary efficacy of an intervention aimed at reducing men’s alcohol use and improving family outcomes. The intervention was delivered in 5 sessions by trained lay-providers, and utilized a combination of behavioral activation, motivational interviewing (MI) and gender norm transformative strategies. The intervention showed preliminary efficacy for addressing alcohol use and family related problems.

Five studies evaluated brief interventions that ranged from 1 to 6 sessions and were delivered by primary HCWs, lay providers and specialist mental health professionals [38, 94, 127, 178, 193]. The brief interventions were reported as feasible, acceptable [38], and efficacious in reducing alcohol consumption [94, 127, 178, 193]. The brief interventions additionally resulted in reductions to IPV, participation in sex work [178], and risky sexual behavior [127].

One study evaluated the efficacy of a mobile delivered MI intervention and found that at 1 month, AUDIT-C scores were significantly higher for waiting-list controls compared to those who received the mobile MI [94].

Moscoe at al. [141] found no effect of a prize-linked savings account on alcohol, gambling and transactional sex expenditures among men.

*Individual-level interventions for khat use*. One study utilized a randomized control trial (RCT) approach to evaluate the effect of a three-session brief intervention for khat use on comorbid psychopathology (depression, PTSD, khat induced psychotic symptoms) and everyday functioning. The intervention was delivered by trained college graduates and was found to result in reduced khat use and increased functioning levels, but had no benefit for comorbidity symptoms (compared to assessments only) [202].

*Individual level intervention for any substance use*. One study evaluated the efficacy of a four-session psychoeducation intervention using an RCT approach. The study found that the intervention was effective in reducing the severity of symptoms of any substance abuse at 6 months compared to no intervention. The intervention was additionally effective in reducing symptoms for depression, hopelessness, suicidality, and anxiety [145].

#### Programs

*Methadone programs*. Two studies utilized qualitative methods to evaluate the perceptions of persons receiving methadone on the benefits of the programs [61, 62]. The methadone programs were perceived as having potential to aid in recovery from opioid use and to reduce HIV transmission among PWID [61, 62].

*Needle-syringe programs (NSPs)*. One paper explored the impact of NSPs programs on needle and syringe sharing among PWID. The study reported that the introduction of NSPs led to significant reductions in needle and syringe sharing [56].

*Tobacco cessation programs*. One study evaluated HCWs knowledge and practices on tobacco cessation and found that the knowledge and practice on tobacco cessation was inadequate [89].

*Out-patient SUD treatment programs*. One paper investigated the impact of community based outpatient SUD treatment services and reported a 42% substance use abstinence rate 0–36 months following treatment termination [84].

#### Population-level interventions

*Population-level tobacco interventions*. One study evaluated the appropriateness and effectiveness of HIC anti-tobacco adverts in the African context and found the adverts to be effective and appropriate [183].

*Population-level alcohol interventions*. One paper examined community members’ perspectives on the impact of the government’s public education messages on alcohol abuse and reported that the messages were ineffective and unpersuasive [55].

A complete description of studies investigating programs and interventions is in Table 3.

**Table 3:** Studies evaluating substance use or SUD interventions and programs.

Author, Year	Study design	Study objective	Sample size	Name of intervention/ program	Intervention delivered by	Outcomes and measures	Main Findings
**Individual-level interventions for harmful alcohol use**:							
Mackenzie et al. 2009 [38]	Mixed methods	Evaluate feasibility of an alcohol screening and brief intervention for adult clients attending HIV VCT centres	Intervention group: 456 Comparison group: 602	5–10 minute brief intervention.	Trained VCT service providers	Acceptability Change in AUDIT scores Proportion of respondents screened for alcohol use and offered feedback	Intervention feasible and acceptable
Papas et al. 2010 [40]	Mixed methods	Cultural adaptation and pilot testing of CBT for alcohol use among HIV-infected outpatients	Focus group 1; 8 Focus group 2; 27	6 sessions of CBT delivered by non-professionals	Paraprofessionals	Treatment attendance Treatment acceptability, -- Alcohol use assessment using the TLFB method	Culturally adapted CBT was feasible, acceptable, and demonstrated preliminary efficacy
Papas et al. 2011 [175]	RCT	Efficacy of CBT for HIV-infected outpatients with hazardous/ binge drinking alcohol	75	6 weekly CBT sessions Control: Usual care	Paraprofessionals	Percent drinking days and mean drinks per drinking days measured using the TLFB method	CBT efficacious
Harder et al. 2020 [94]	RCT	To test the effectiveness of a MI intervention using the mobile phone among adults with alcohol use problems.	Intervention group: 89 Control group 1: 65 Control group 2: 76	Mobile MI–single session MI delivered via mobile phone call upon enrolment Control 1: in-person MI Control 2: delayed mobile MI	Three clinicians with Master’s degree in nursing, doctoral degree in clinical psychology and a medical degree	Change in AUDIT-C scores	AUDIT-C scores significantly higher for waiting-list controls after 1 month of no intervention versus mobile MI 1 month after intervention. no difference between in-person and mobile MI at 1 month
Moscoe et al. 2019 [141]	RCT	To evaluate the effect of prize-linked savings accounts on men’s expenditure on alcohol use and risky sexual behaviors	Intervention: group: 152 Control group: 148	Intervention: Reward for saving any amount in the bank Control: No reward standard interest	-	Whether a participant saved any money in the bank account during the study period; total amount saved in the bank account; expenditures on alcohol, gambling, and transactional sex.	The intervention did not have a significant effect on alcohol, gambling, and transactional sex expenditures.
Giusto et al. 2020 [90]	Non-concurrent multiple baseline design	To evaluate the preliminary efficacy of an intervention aimed at reducing men’s alcohol use and improving family outcomes	9	5 session brief intervention combining behavioral activation, MI and gender norm transformative strategies Control: None	Trained lay counselors	Changes in daily alcohol use (TLFB) Changes in PHQ-9 scores Changes in family-oriented behavior	Intervention showed preliminary efficacy for addressing alcohol use and family-related problems
L’Engle et al. 2014 [127]	RCT	Efficacy of a brief intervention for harmful alcohol use for female sex workers	Intervention group: 410 Control group: 408	Intervention group: 6 counselling sessions based on WHO Brief Intervention for alcohol use Control: 6 sessions Nutritional counselling	Trained nurses	Difference in AUDIT scores and laboratory STI results between intervention and control groups	Intervention efficacious in reducing alcohol use and risky sexual behavior.
Parcesepe et al. 2016 [178]	RCT	To document the impact of an alcohol harm reduction intervention on IPV and engagement in sex work among FSWs	Intervention group: 410 Control group: 408	Intervention: 6 sessions of contextualized WHO Brief Intervention Control: 6 sessions of non-alcohol related nutrition intervention	Trained nurses	Differences in interpersonal violence and engagement in sex work between intervention and control groups	Intervention resulted in reduction in IPV, reduction in sexual partners and reduction in participation in sex work
Takahashi et al. 2018 [193]	3-arm quasi experimental	To assess the effectiveness of community-based alcohol brief intervention with and without motivational talks by former drinkers, in reducing harmful and hazardous alcohol use	Control group: 52 Intervention group 1: 52 Intervention group 2: 57	Intervention 1: 3 sessions brief intervention based on FRAMES model Intervention 2: 3 sessions BI plus group Motivational talks Control: general health information on alcohol consumption.	Trained community-health workers	Differences in the mean AUDIT scores between the control group and each of the intervention groups at 1, 3 and 6 months,	Greater reduction in adjusted mean AUDIT scores in intervention groups compared to controls
**Individual-level interventions for khat use**							
Widmann et al. 2017 [202]	RCT	To evaluate impact of a brief intervention for khat use on comorbid psychopathology (depression, PTSD, khat induced psychotic symptoms) and everyday functioning	Intervention group: 161 Control group: 169	Intervention: 3 sessions Screening and Brief Intervention Control: Assessments for comorbidity and SBI after 2 months	Trained college graduates	Differences in PHQ-9; Post-traumatic diagnostic Scale, ASSIST and everyday functioning scores	Intervention reduced khat use and increased functioning levels but had no benefit for comorbidity symptoms
**Individual-level interventions for any substance use**							
Muriungi & Ndetei 2013 [145]	RCT	Effectiveness of psycho-education on depression, hopelessness, suicidality, anxiety and substance use among college students	Intervention group: 1,181 Control group: 1,926	4 Psycho education sessions Control: No intervention	Clinical psychologist	Differences in BDI, BHS, BSIS, BAI, ASSIST scores between intervention and control group	Psycho-education was effective in reducing the severity of depression symptoms, hopelessness, suicidality, anxiety and risk of substance abuse at 6 months.
**Programs**							
Methadone programs							
Rhodes 2018 [62]	Qualitative	To evaluate perceptions of persons receiving methadone as regards benefits of the methadone programs	30	Methadone programs	-	Perceptions on the recovery potential of methadone programs	Methadone perceived as having recovery potential.
Rhodes et. al 2015 [61]	Qualitative methods and mathematical modeling	To document the HIV prevention impact of Opioid Substitution Therapy with methadone form the perspective of PWID use	109	Opioid substitution therapy with methadone	-	Perceptions of PWID on promise of methadone Projected HIV effects of methadone	Methadone could be an important component of any intervention package aiming to reduce HIV transmission among PWID in Kenya.
Needle syringe programs							
Ndimbii et al. 2015 [56]	Qualitative	To explore the impact of needle and syringe programs on needle and syringe sharing among PWID use	109	Needle and syringe programs	-	Needle and syringe sharing practices before and after needle and syringe programs	Introduction of needle and syringe programs led to significant reductions in needle and syringe sharing.
Tobacco cessation programs							
Gichuki et al. 2016 [89]	Cross-sectional	To determine the smoking cessation practices of healthcare providers working in public health facilities; training received and barriers to provision of interventions	400	Smoking cessation practices	-	Smoking cessation practices; training received; barriers to practice	Practice of smoking cessation interventions was sub-optimal; insufficient training was reported as an important barrier
Substance use out-patient programs							
Deveau et al. 2010 [84]	Cross-sectional	Evaluate utilization of out-patient addiction services at 4 community-based clinics	1,847	Addiction out-patient treatment services	-	Number of clients utilizing services over a 4-year period Abstinence rates	Number of clients participating in treatment services increased from 35 to 479 over the 4-year period 42% reported abstinence from substance use over a 0-36-month period
**Population level-interventions for tobacco use**							
Perl et al. 2015 [183]	Mixed methods	An assessment of effectiveness and ease of adaptation of anti-tobacco adverts developed in HICs from the perspective of adult smokers and non-smokers	1078	Radio and TV anti-tobacco adverts	-	Ratings of effectiveness and ease of adaptation of anti-tobacco ads	Adverts developed in High Income Countries are viable in tobacco control in Africa
**Population level-interventions for alcohol use**							
Muturi et al. 2016 [55]	Qualitative	To explore community perspectives on alcohol abuse prevention strategies in rural Kenya	60	Alcohol abuse prevention strategies	-	Perspectives on alcohol abuse prevention strategies in rural Kenya	Rural communities viewed alcohol abuse prevention interventions as ineffective and messages as unpersuasive in changing this high-risk behavior.

### Studies qualitatively exploring various substance use or SUD topics (other than interventions)

#### General description of qualitative studies

There were 23 qualitative studies included in our review. The studies were conducted between 2004 and 2020. Data was collected using several approaches including in-depth interviews (IDIs) only (n = 6), focus group discussions (FGDs) only (n = 2), a combination of FGDs and IDIs (n = 10), a combination of observation and individual IDIs (n = 2), a combination of observation, IDIs and FGDs (n = 1), a combination of literature review, observation, IDIs and FGDs (n = 1). One study utilized the participatory research and action approach [60]. The target populations for the qualitative studies included persons using heroin (n = 3), males and females with IDU (n = 11) adolescents and youth (n = 3), FSWs (n = 2), refugees and Internally Displaced Persons (IDPs) (n = 1), and PLHIV (n = 2).

#### Injecting drug use and heroin use

Thirteen studies explored various themes related to IDU and heroin use with most of them (n = 8) focusing on issues related to women. Three studies explored the drivers of IDU among women and found them to include influence of intimate partners [48, 49], stress of unexpected pregnancies [49], gender inequality, and social suffering [67]. One study found that IDU among women interfered with utilization of antenatal and maternal and child health services [57], while another reported that women who inject drugs linked IDU to amenorrhea hence did not perceive the need for contraception [51].

Mburu et al [47] explored the social contexts of women who inject drugs and found that these women experienced internal and external stigma of being injecting drug users, and external gender-related stigma of being female injecting drug users. Using a socio-ecological approach, Mburu et al [50] reported that IDU during sex work was an important HIV risk behavior. In another study, FSWs reported that they used heroin to boost courage to engage in sex work [65].

Other than IDU and heroin use among women, five studies investigated other themes. One study explored the experiences of injecting heroin users and found that the participants perceived heroin injection as cool [42]. Guise et al. 2015 [44] conducted a study to explore transitions from smoking to injecting and reported that transitions from smoking to IDU were experienced as a process of managing resource constraints, or of curiosity, or search for pleasure. One study explored the experiences of persons on MMT as regards integration of MMT with HIV treatment. The study was guided by the material perspective in sociology theory and Annmarie’s Mol’s analysis of logic of care. Persons on MMT preferred that they have choice over whether to seek care for HIV and MMT in a single, or in separate settings.

#### Alcohol use

Six studies focused on alcohol use. Three studies explored perceptions of service providers and communities on the effects of alcohol use. Alcohol use was perceived as having a negative impact on sexual and reproductive health [53, 54] and on socio-economic status [43, 46]. One study explored the reasons for alcohol use among PLHIV and found that reasons for alcohol use included stigma and psychological problems, perceived medicinal value, and poverty [60].

#### Youth and adolescent substance use

Three studies focused on substance use among youth and adolescents. In one study, the adolescents perceived that substance use contributed to risky sexual behavior including unprotected sex, transactional sex, and multiple partner sex [58]. The youth identified porn video shows and local brew dens as places where risky sexual encounters between adolescents occurred [59]. Ssewanyana et al. [63] utilized the socio-ecological model to explore perceptions of adolescents and stakeholders on the factors predisposing and contributing to substance use. Substance use among adolescents was perceived to be common and to be due to several socio-cultural factors e.g. access to disposable income, idleness, academic pressure, low self-esteem etc.

#### Other topics

Utilizing the syndemic theory, one study explored how substance use, violence and HIV risk affect PrEP (Pre-exposure prophylaxis) acceptability, access and intervention needs among male and female sex workers. The study found that co-occurring substance use, and violence experienced by sex workers posed important barriers to PrEP access [41].

A complete description of included qualitative studies is in Table 4.

**Table 4:** Studies qualitatively exploring various substance use or SUD related themes.

Author, Year	Study objective	Methods of data collection; Study setting & study population	Age and gender distribution	Theoretical frameworks employed	Main findings
**Injecting drug use and heroin use**					
Yotebieng et al. 2016 [67]	To explore the reproductive health of women of childbearing age who inject drugs and its implications for healthcare	IDIs with 17 women who inject drugs	Age range 20–35 years	Social-ecological theory	Gender inequality and social suffering were reported as driving factors of continued use during pregnancy; healthcare interactions reported as biased toward HIV screening over alcohol and drug screening and education.
Beckerleg 2004 [42]	To describe the experiences of injecting heroin users	A combination of anthropology and ethnographic approaches IDIs with 40 persons with injecting heroin use Observation of injecting users in streets and alleys	Age and gender distribution not reported	No theoretical framework mentioned	Heroin injection was perceived as “cool”; Most users were ill-informed on risk of transmission of HIV through injecting practices.
Guise et al. 2015 [44]	To explore accounts of transitions from smoking to injecting to understand the role of individual, social and structural processes	The study combined data from two separate studies conducted in Kenya: 1) an in-depth qualitative study of HIV care access for people who inject drugs (n = 118) 2) an ethnographic study of the political economy of the heroin trade in Kenya (n = 92)	Study 1: Age range: 19–49 years; Male 72% Study 2: Age distribution not reported; Male 94%	No theoretical framework mentioned	Transitions from smoking to IDU are experienced as a process of managing a series of resource constraints or of curiosity or search for pleasure.
Mburu et al. 2018a [51]	To explore perspectives of women and stakeholders on the intersection between drug use and contraceptive use	IDIs and FGDs with 45 women who inject drugs and 5 stakeholders involved in service provision	Age range 19–56 years Gender distribution of stakeholders not reported	No theoretical framework mentioned	Women linked drug use to amenorrhea hence did not perceive need for contraception
Mburu et al. 2018b [47]	to explore the needs and social contexts of women who inject drugs in coastal Kenya	IDIs and FGDs with 45 women who inject drugs and 5 stakeholders involved in service provision	Age range for women & stakeholders 19–56 years PWID 100% female; gender distribution of stakeholders not reported	No theoretical framework mentioned	Several forms of external and self-stigma are experienced by women with IDU. These included internal and external stigma of being a drug user, external gender-related stigma of being a female injecting drug user and external stigma of being HIV positive among participants living with HIV.
Mburu et al. 2019a [48]	To document the role of intimate partners in influencing IDU among women	Secondary analysis of a cross sectional qualitative study by Mburu et al 2018 [47] Original study involved IDIs and FGDs with 45 women who inject drugs and 5 stakeholders involved in service provision	Age range for women & stakeholders 19–56 years PWID 100% female; gender distribution of stakeholders not reported	Social-ecological theory	Intimate partners wield significant influence, on the initiation and maintenance of drug use by women; this influence is mediated by inequitable economic and gender-power.
Mburu et al. 2020 [49]	To explore factors influencing women’s decisions to use drugs during pregnancy	Secondary analysis of a cross sectional qualitative study by Mburu et al 2018 [47] IDIs and FGDs with 45 women who inject drugs and 5 stakeholders involved in service provision	Age range for women & stakeholders 19–56 years PWID 100% female; gender distribution of stakeholders not reported	No theoretical framework mentioned	Women used drugs to cope with stress of unexpected pregnancies, to manage withdrawals. Intimate partners also played roles in facilitating or limiting substance use.
Mburu et al. 2019b [50]	To document HIV risks among women who inject drugs in coastal Kenya	Secondary analysis of a cross sectional qualitative study by Mburu et al 2018 [47] IDIs and FGDs with 45 women who inject drugs and 5 stakeholders involved in service provision	Age range for women & stakeholders 19–56 years PWID 100% female; gender distribution of stakeholders not reported	Social-ecological theory	IDU during sex work emerged as an important HIV risk behavior
Ndimbii et al. 2018 [57]	To explore utilization of reproductive, maternal, neonatal and child health services among women who inject drugs in coastal Kenya	IDIs and FGDs with 45 women who inject drugs and 5 stakeholders involved in service provision in two coastal towns.	Age range 19–56 years Gender distribution of stakeholders not reported	No theoretical framework mentioned	Drug use interfered with utilization of antenatal and maternal and child health services
Syvertsen et al. 2016 [64]	To explore the emergent drug market in Kisumu, western Kenya, from the perspective of PWIDs	Ethnographic methods; 29 IDIs; 151 quantitative surveys with community members reporting IDU	151 survey participants: mean age 28.8 years; Male 84% Qualitative sample: Mean age 26.7 years; Male 55%	No theoretical framework mentioned	The drug market in Kisumu is dynamic and chaotic reflecting the fluid and adaptive characteristics typical of new drug markets. The drug market is also hidden, erratic, and expensive
Mital et al. 2016 [52]	To describe heroin user’s experiences during a period of heroin shortage	Rapid assessment methods: 66 KIIs and 15 FGDs with heroin users	At least 18 years of age. Gender distribution not reported	No theoretical framework mentioned	During the shortage, there was desperation and uncertainty, prices for heroin increased, purity decreased, and drug substitution and poly-drug use were practiced. Users transitioned from smoking to injection of heroin during the shortage to compensate for the low quality and quantity.
Guise et al. 2019 [45]	To explore experiences of service users on integrated HIV care and methadone treatment	30 persons on MMT	Mean age: 34 years Male 70%	Material perspective in sociology and Annemarie Mol’s analysis of logic of care	Service users preferred that they have choice over whether to seek care for HIV and MMT in a single setting, or separate settings.
Syvertsen et al. 2019 [65]	To explore heroin use among FSWs in Kenya to inform services	IDIs with 45 FSWs	Age range: 18–37 years Female 100%	Addiction trajectories concept	Women commonly smoked cocktails containing heroin while using alcohol and other drugs prior to sex work. Most women perceived heroin to boost courage to engage in sex work. Sex work reinforced drug use in ways that both managed and created new risks.
Alcohol use					
Ezard et al. 2011 [43]	To describe the burden and pattern of substance use among refugees and IDPs from the perspective of community members and service providers, and identify available resources and interventions for managing the substance use in this population	Rapid assessment and response (RAR) Literature review; 20 Key informant interviews, 14 FGDs (n = 5–12) and 3 group discussions (n-20-34) with substance users; service providers; sex workers; young people; teachers; PLHIV; post-voluntary counselling and testing groups; health workers; pre-formed community groups Direct observation at refugee/IDP sites.	Gender distribution not reported Age range: 17–57 years	No theoretical framework mentioned	Use of alcohol within these populations was widespread and was linked to a range of health and socio-economic problems. Displacement experiences, may make communities vulnerable to substance use and its impact. Access to health services for this population was limited.
Muturi 2014 [53]	To explore the perceived reproductive health risks associated with alcoholism from the perspective of rural communities in Kenya	Culture-centred approach that emphasizes community engagement in development of interventions; IDIs with 12 opinion leaders and 7 FGDs with 60 community members	Opinion leaders: Age distribution not reported 67% male Community members: Age range 25–57 years 50% male	No theoretical framework mentioned	Heavy alcohol use has severe consequences on sexual and reproductive health
Muturi 2015 [54]	To explore rural communities’ perspectives on the risk factors for HIV infection among women in alcohol discordant relationships	60 participants recruited from community-based organizations participated in 7 FGDs	Age range 27–57 years Males 50%	Protection motivation theory	The perceived impact of alcoholism on men’s reproductive health and the unmet sexual and reproductive needs of women in alcohol discordant relationships drive women to engage in risky sexual behaviors.
Kibicho & Campbell 2019 [46]	To explore the effect of second-generation alcohol consumption on sexual risk behaviors, alcohol misuse, violence and economic stress factors, and HIV infection risk.	12 FGDs of 80 people from established support groups	At least 18 years of age Male 57.5%	Social-ecological theory and syndemic theory	Second-generation alcohol consumption is prevalent and has profound socio-economic and health effects on households.
Velloza et al. 2015 [66]	To describe the stages and processes of change utilized by FSWs participating in an alcohol-reduction intervention	IDIs with 45 FSWs	Age range: 19–48 years Female 100%	Stages of change model	In sessions 1–3, most participants were in the pre-contemplation, contemplation, or preparation stages. In sessions 4–6, most participants were in the action and maintenance stages. In the pre-contemplation stage, participants reported using environmental re-evaluation, consciousness raising, and dramatic relief techniques. In contemplation/ preparation phase, participants said they used self-reevaluation and self-liberation techniques. In action/maintenance, participants reported using helping relationships, counter-conditioning, reinforcement management, and stimulus control strategies.
Othieno et al. 2012 [60]	To explore the factors related to harmful alcohol use and identify interventions aimed at improving adherence to antiretroviral drugs among PLHIV who also use alcohol in a harmful way	Participatory Action Research tools; FGDs with 67 PLHIV and also abusing alcohol and 19 community members drawn from support groups working with PLHIV	Age and gender distribution not reported	No theoretical framework mentioned	Reasons for alcohol use included stigma, to gain social acceptance, to deal with psychological problems, perceived medicinal value, and physical addiction and poverty. Screening and treatment interventions within the community were scarce
Youth and adolescent substance use					
Njue et al. 2009 [58]	To describe the phenomenon of disco funerals as the setting of risky sexual encounters among youth.	IDIs with 150 adolescents drawn from the community; Observation at 6 disco funerals and 42 places where youth hang-out.	Age range: 15–20 years Male 50%	No theoretical framework mentioned	Drugs and alcohol seemed to facilitate risky unprotected, multiple-partner, coerced, and transactional sex.
Njue et al. 2011 [59]	To explore risk situations that can explain the high HIV prevalence among youth in Kisumu town, Kenya	IDIs with 150 adolescents; 4 FGDs and 48 observations at places where youth spend their free time.	Age range: 15–20 years Male 50%	No theoretical framework mentioned	Porn video shows and local brew dens were identified as popular events where unprotected multi-partner, concurrent, coerced and transactional sex occurs between adolescents.
Ssewanyana et al. 2018 [63]	To explore perceptions of young people and stakeholders on the types of substances used and the predisposing and protective factors	11 FGDs with 85 young people (78 adolescents and 7 young adult community representatives); IDIs with 10 stakeholders	Adolescents: aged 10–19 years; 42 males and 36 females Young adult representatives: aged 22–28 years; 3 males and 4 females Stakeholders: Aged 27–51 years; 4 male and 6 females	Social-ecological theory	The use of various substances was common among adolescents. Substance use was due to several interacting social, cultural and community factors e.g. access to disposable income, idleness, academic pressure, low self-esteem, use by close family members etc.
Other topics explored					
Bazzi et al. 2019 [41]	To explore how substance use, violence and HIV risk shape PrEP acceptability, access and intervention needs among sex workers	73 Female and male sex workers	Median age (IQR): Female 28 (18 to 42), Male 25 (19 to 41) Male 38.4%	Syndemic theory	Syndemic substance use and violence experienced by sex workers posed important barriers to PrEP access for sex workers.

## Discussion

This is to our knowledge, the first study to summarize empirical work done on substance use and SUDs in Kenya. More than half (77.8%) of the reviewed studies investigated the area of prevalence and risk factors for substance use. Less common were qualitative studies exploring various themes (12.4%) and studies evaluating interventions and programs (9.7%). The first study was conducted in 1982 and since then the number of publications has gradually risen. Most of the research papers (92.4%) were of moderate to high quality. In comparison to two recent scoping reviews conducted in South Africa and Botswana, more research work has been done on substance use in Kenya. Our study found that 185 papers on substance use among Kenyans had been published by the time of the search while Opondo et al. [11] and Tran et al. [10] reported that only 53 and 7 papers focusing on substance use had been published in South Africa (between 1971 and 2017) and in Botswana (between 1983 and 2020) respectively.

### Epidemiology of substance use or SUD

Studies investigating the prevalence, and risk factors for substance use dominated the literature. The studies, which were conducted across a broad range of settings and populations, focused on various substances including alcohol, tobacco, cannabis, opioids, cocaine, sedatives, inhalants, hallucinogens, prescription medication, and ecstasy. In addition, a wide range of important health and socio-demographic factors were examined for their association with substance use. Most studies had robust sample sizes and were conducted using diverse designs including cross-sectional, case-control and cohort. The studies showed a significant burden of substance use among both adults and children and adolescents. In addition, substance use increased the odds of negative mental and physical health outcomes consistent with findings documented in global reports [2, 3]. These findings highlight the importance of making the treatment and prevention for substance use and SUDs of high priority in Kenya.

- Two main evidence gaps were identified within this category: The prevalence and risk factors for substance use among certain vulnerable populations for whom substance use can have severe negative consequences, had not been investigated. For example, no study had included police officers or persons with physical disability, only one study had its participants as pregnant women [113], and only 2 studies had been conducted among HCWs [140, 196].
- Few studies had explored the epidemiology of hallucinogens, prescription medication, ecstasy, IDU, and emerging substances e.g. synthetic cannabinoids. These substances are a public health threat globally [207, 208] yet their use remains poorly documented in Kenya.

### Interventions and programs

Given the significant documented burden of substance use and SUDs in Kenya, it was surprising that few studies had focused on developing and testing treatment and prevention interventions for SUDs. A possible reason for this is limited expertise in the area of intervention development and testing. For example, research capacity in implementation science has been shown to be limited in resource-poor settings such as ours [209].

Of note is that most of the tested interventions had been delivered by lay providers [40, 90, 175] and primary HCWs [38, 127, 178] indicating a recognition of task-shifting as a strategy for filling the mental health human resource gap in Kenya.

Several research gaps were identified within this category.

- Out of the 11 individual-level interventions tested, nine had targeted harmful alcohol use except one which focused on khat [202] and another that targeted several substances [145]. No studies had evaluated individual-level interventions targeting tobacco and cannabis use, despite the two being the second and third most commonly used substances in Kenya [8]. Further, no individual-level interventions had focused on other important SUDs like opioid, sedative and cocaine use disorders.
- Few studies had evaluated the impact of substance use population-level interventions [55, 183]. Several cost-effective population-level interventions have been recommended by WHO e.g. mass media education and national toll free quit line services for tobacco use, and brief interventions integrated into all levels of primary care for harmful alcohol use [210]. Such strategies need to be tested for scaling up in Kenya.
- None of the interventions had been tested among important vulnerable populations for whom local research already shows a significant burden e.g. children and adolescents, the Lesbian Gay Bisexual Transgender & Queer (LGBTQ) community, HCWs, prisoners, refugees, and IDPs. In addition, no interventions had been tested for police officers and pregnant women, and no studies had evaluated interventions to curb workplace substance use.
- Only one study evaluated digital strategies for delivering substance use interventions [94] yet the feasibility of such strategies has been demonstrated for other mental health disorders in Kenya [211]. Moreover, the time is ripe for adopting such an approach to substance use treatment given the fact that the country currently has a mobile subscriptions penetration of greater than 90% [212].
- No studies had evaluated the impact of other interventions such as mindfulness and physical exercise. Meta-analytic evidence suggests that such strategies hold promise for reducing the frequency and severity of substance use and craving [213, 214].

### Qualitative studies

The qualitative studies focused on a broad range of themes including drivers and impact of substance use, drug markets, patterns of substance use, stigma, and access to treatment. Most of the work however focused on PWID and heroin users. Future qualitative work should explore issues relating to other populations for example persons with other mental disorders, persons with physical disabilities, police officers, and persons using other commonly used substances such as tobacco, khat, and cannabis.

### Limitations

The aim of this systematic review was to provide an overview of the existing literature on substance use and SUD research in Kenya. We therefore did not undertake a meta-analysis and detailed synthesis of the findings of studies included in this review. In addition, variability in measurements of substance use outcomes precluded our ability to more comprehensively summarize the study findings. For quality assessment, detailed assessments using design specific tools were not possible given the diverse methodological approaches utilized in the studies. We therefore used a single tool for the quality assessment of all studies. The results of the quality assessment are therefore to be interpreted with caution. Nonetheless this review describes for the first time the breadth of existing literature on substance use and SUDs in Kenya, identifies research gaps, and provides important directions for future research.

## Conclusion

The purpose of this systematic review was to map the research that has been undertaken on substance use and SUDs in Kenya. Epidemiological studies dominated the literature and indicated a significant burden of substance use among both adults and adolescents. Our findings indicate that there is a dearth of literature regarding interventions for substance use and we are calling for further research in this area. Specifically, interventions ought to be tested not just for alcohol but for other substances as well, and among important at risk populations. In addition, future research ought to explore the feasibility of delivering substance use interventions using digital means, and the benefit of other interventions such as mindfulness and physical exercise. Future qualitative work should aim at providing in-depth perspectives on substance use among populations excluded from existing literature e.g. police officers, persons using other substances such as tobacco, cannabis and khat, and persons with physical disability.

## Supporting information

S1 Checklist PRISMA checklist. (DOCX)

S1 File Search terms for PsychINFO. (PDF)

## Abbreviations

ASI: Addiction Severity Index
ASSIST: Alcohol Smoking and Substance Involvement Screening Test
AUD: Alcohol Use Disorder
AUDIT: Alcohol Use Identification Test
AUDIT-C: Alcohol Use Identification Test–Concise
BAI: Beck Anxiety Inventory
BDI: Beck Depression Inventory
BHS: Behavioral Health Screen
BMI: Body Mass index
BSIS: Beck Suicidal Intent Scale
CAD: Coronary Artery Disease
CAGE: Cut, Annoyed, Guilty, Eye-opener
CIDI: Composite International Diagnostic Interview
CINAHL: Cumulative Index of Nursing and Allied Professionals
CRAFFT: Car, Relax, Alone, Forget, Friends, Trouble
DAST: Drug Abuse Screening Test
DSM-III: Diagnostic & Statistical Manual Third Edition
DSM-III R: Diagnostic & Statistical Manual Third Edition Revised
DSM-IV: Diagnostic & Statistical Manual Fourth Edition
DSM-V: Diagnostic & Statistical Manual Fifth Edition
DUSI-R: Drug Use Screening Inventory—Revised
FGD: Focus Group Discussion
FSW: Female Sex Workers
GSHS: Global School-based Health Survey
HCV: Hepatitis C Virus
HCW: Healthcare worker
HIC: High Income Country
HIV: Human Immunodeficiency Virus
ICD: International Classification of Disease
IDI: In-depth Interviews
IDP: Internally Displaced Persons
IPV: Intimate Partner Violence
KIIs: Key Informant Interviews
K-SADS: Kiddie-Schedule for Affective Disorders
LGBTQ: Lesbian, Gay, Bisexual, Transgender, Queer
LMIC: Low and Middle Income Country
MAST: Michigan Alcohol Screening Test
MI: Motivational Interviewing
MINI: Mini International Neuropsychiatric Interview
MMT: Methadone Maintenance Therapy
MPBI: Multiple Problem Behavior Inventory
MSM: Men who have Sex with Men
MSME: Men who have Sex with Men Exclusively
MSMW: Men who have Sex with Men & Women
NIH: National Institute of Health
NSP: Needle Syringe Program
OST: Opioid Substitution Therapy
PLHIV: People Living with HIV
PrEP: Pre-exposure Prophylaxis
PTSD: Post-Traumatic Stress Disorder
PWID: People Who Inject Drugs
QATSDD: Quality Assessment Tool for Studies with Diverse Designs
RCT: Randomized controlled trial
RTAs: Road Traffic Accidents
SCID: Structured Clinical interview for DSM
SES: Socio-economic Status
SSA: Sub-Saharan Africa
TB: Tuberculosis
UNODC: United Nations Office on Drugs and Crime
VCT: Voluntary Counseling & Testing
WOTC: Wisdom of the Crowds

## References

[1] James SL, Abate D, Abate KH, Abay SM, Abbafati C, Abbasi N, et al. Global, regional, and national incidence, prevalence, and years lived with disability for 354 Diseases and Injuries for 195 countries and territories, 1990–2017: A systematic analysis for the Global Burden of Disease Study 2017. Lancet. 2018 Nov 10;392(10159):1789–858. doi: 10.1016/S0140-6736(18)32279-7 30496104 PMC6227754

[2] Hammer JH, Parent MC, Spiker DA, World Health Organization. Global status report on alcohol and health 2018. https://apps.who.int/iris/bitstream/handle/10665/274603/9789241565639-eng.pdf?ua=1. Accessed 12 June 2021

[3] World Health Organisation. Tobacco [Internet]. 2020. https://www.who.int/news-room/fact-sheets/detail/tobacco. Accessed 12 June 2021

[4] World Health Organization. Alcohol and drug use disorders: Global health estimates. 2017. http://www.who.int/substance_abuse/activities/fadab/msb_adab_2017_GHE_23June2017.pdf. Accessed 12 June 2021

[5] McKetin R, Leung J, Stockings E, Huo Y, Foulds J, Lappin JM, et al. Mental health outcomes associated with of the use of amphetamines: A systematic review and meta-analysis. EClinicalMedicine. 2019;16:81–97. Accessed 11 May 2022 doi: 10.1016/j.eclinm.2019.09.014 31832623 PMC6890973

[6] Lowe DJE, Sasiadek JD, Coles AS, George TP. Cannabis and mental illness: a review. European Archives of Psychiatry and Clinical Neuroscience. 2019; 269,107–20. doi: 10.1007/s00406-018-0970-7 30564886 PMC6397076

[7] International Narcotics Control Board. Chapter 1: Economic consequences of drug abuse. 2013. https://www.incb.org/documents/Publications/AnnualReports/AR2013/English/AR_2013_E_Chapter_I.pdf. Accessed 12 June 2021

[8] National Authority for the Campaign Against alcohol and Drug Abuse. Rapid Situation Assessment of Drugs abd Substance Abuse in Kenya. 2017. https://nacada.go.ke/sites/default/files/2019-10/National%20ADA%20Survey%20Report%202017_2_2.pdf. Accessed 12 June 2021

[9] National Authority for the Campaign Against alcohol and Drug Abuse, Kenya Institute for Public Policy Research and Analysis. Status of Drugs and Substance Abuse among Primary School Pupils in Kenya. 2019. https://nacada.go.ke/sites/default/files/2019-10/Report%20on%20the%20Status%20of%20Drugs%20and%20Substance%20Abuse%20among%20Primary%20School%20Pupils%20in%20Kenya.pdf. Accessed 12 June 2021

[10] Opondo PR, Olashore AA, Molebatsi K, Othieno CJ, Ayugi JO. Mental health research in Botswana: a semi-systematic scoping review. *J Int Med Res*. 2020;48[10]:300060520966458. doi: 10.1177/0300060520966458 33115301 PMC7607297

[11] Tran BX, Moir M, Latkin CA, Hall BJ, Nguyen CT, Ha GH, et al. Global research mapping of substance use disorder and treatment 1971–2017: Implications for priority setting. *Subst Abus Treat Prev Policy*. 2019; 14[1]:21. doi: 10.1186/s13011-019-0204-7 31101059 PMC6525403

[12] Dhadphale M, Mengech HN, Syme D, Acuda SW. Drug abuse among secondary school students in Kenya: a preliminary survey. East Afr Med J. 1982;59[2]:152–6. . 6982160

[13] The National Authority for the Campaign aganist Alcohol and Drug Abuse [Internet]. http://www.nacada.go.ke. Accessed 12 June 2021

[14] The National Authority for the Campaign Aganist Alcohol and Drug Abuse. African Journal of Alcohol & Drug Abuse (Volume 2). https://nacada.go.ke/sites/default/files/AJADA/AJADA%202%20ammended/AJADA%20Volume%20II%20(Full%20Booklet).pdf. Accessed 12 june 2021

[15] Republic of Kenya. The National Treasury and Planning. Third Medium Term Plan 2018–2022. Kenya Vision 2030. https://planning.go.ke/wp-content/uploads/2018/12/THIRD-MEDIUM-TERM-PLAN-2018-2022.pdf. Accessed 20 June 2020

[16] United Nations Development Programme. The 2030 Agenda for Sustainable Development, A/RES/70/1. Undp. 2015.

[17] Liberati A, Altman D G, Tetzlaff J, Mulrow C, GÃ¸tzsche P C, Ioannidis J P A et al. The PRISMA statement for reporting systematic reviews and meta-analyses of studies that evaluate healthcare interventions: explanation and elaboration. BMJ. 2009; 339: b2700 doi: 10.1136/bmj.b2700 19622552 PMC2714672

[18] The National Authority for the Campaign Aganist Alcohol and Drug Abuse. African Journal of Alcohol & Drug Abuse (Volume 3). https://nacada.go.ke/sites/default/files/AJADA/AJADA%203/NACADA%20AJADA%20Vol%203-%20Full%20Booket.pdf. Accessed 12 june 2021

[19] The National Authority for the Campaign Aganist Alcohol and Drug Abuse. African Journal of Alcohol & Drug Abuse (Volume 1). https://nacada.go.ke/sites/default/files/AJADA/AJADA%201%20ammended/AJADA%20Volume%20I%20(Full%20Booklet).pdf. Accessed 12 june 2021

[20] Ouzzani M, Hammady H, Fedorowicz Z, Elmagarmid A. Rayyan—a web and mobile app for systematic reviews. Systematic Reviews (2016) 5:210, doi: 10.1186/s13643-016-0384-4 27919275 PMC5139140

[21] Sirriyeh R, Lawton R, Gardner P, Armitage G. Reviewing studies with diverse designs: The development and evaluation of a new tool. J Eval Clin Pract. 2012;18[4]:746–52. doi: 10.1111/j.1365-2753.2011.01662.x 21410846

[22] Alele F, Malau-Aduli B, Malau-Aduli A, Crowe M. Systematic review of gender differences in the epidemiology and risk factors of exertional heat illness and heat tolerance in the armed forces. BMJ Open. 2020;10[4]:1–10. doi: 10.1136/bmjopen-2019-031825 32265238 PMC7245403

[23] Aden A, Dimba EAO, Ndolo UM, Chindia ML. Socio-economic effects of khat chewing in North Eastern Kenya. East Afr Med J. 2006;83[3]:69–73. doi: 10.4314/eamj.v83i3.9400 16771102

[24] Gitatui M, Kimani S, Muniu S, Okube O. Determinants of harmful use of alcohol among urban slum dwelling adults in Kenya. Afr Health Sci. 2019;19[4]:2906–25. doi: 10.4314/ahs.v19i4.12 32127866 PMC7040319

[25] Njuguna J, Olieva S, Muruka C, Owek C. Khat Consumption in Masalani Town, Northeastern Kenya. J Psychoactive Drugs. 2013;45[4]:355–9. doi: 10.1080/02791072.2013.825516 24377175

[26] Thuo J, Ndetei DM, Maru H, Kuria M. The prevalence of personality disorders in a Kenyan inpatient sample. J Pers Disord [Internet]. 2008;22[2]:217–20. doi: 10.1521/pedi.2008.22.2.217 18419240

[27] Dhadphale M. Alcoholism among outpatients with psychiatric morbidity. Indian J Psychiatry. 1997;39[4]:300–3. 21584096 PMC2967162

[28] Ndetei DM, Khasakhala L, Maru H, Pizzo M, Mutiso V, Ongecha-Owuor FA, et al. Clinical epidemiology in patients admitted at Mathari Psychiatric Hospital, Nairobi, Kenya. Soc Psychiatry Psychiatr Epidemiol. 2008;43[9]:736–42. doi: 10.1007/s00127-008-0360-y 18465102

[29] Kisilu J., Ayuya S., Ndolo J., Mwavua S. Prevalence And Patterns Of Early Drug Abuse Among Clients Attending Ngara Medically Assisted Therapy Clinic Nairobi, Kenya—A Retrospective Study. AJADA. 2019;1.

[30] Maru HM, Kathuku DM, Ndetei DM. Substance use among children and young persons appearing in the Nairobi Juvenile Court, Kenya. East Afr Med J. 2003; 80: 598–602. 15248681

[31] Kanyanya IM, Othieno CJ, Ndetei DM. Psychiatric morbidity among convicted male sex offenders at Kamiti Prison, Kenya. East Afr Med J. 2007;84[4]:151–5. doi: 10.4314/eamj.v84i4.9518 17894248

[32] Macigo FG, Gathece LW, Guthua SW, Njeru EK, Wagaiyu EG, Mulli TK. Oral hygiene practices and risk of oral leukoplakia. East Afr Med J. 2006;83[4]:73–8. doi: 10.4314/eamj.v83i4.9419 16863001

[33] Micheni M, Rogers S, Wahome E, Darwinkel M, Van Der Elst E, Gichuru E, et al. Risk of sexual, physical and verbal assaults on men who have sex with men and female sex workers in coastal Kenya. Aids. 2015;29[0 3]:S231–6. doi: 10.1097/QAD.0000000000000912 26562812 PMC4706373

[34] Ndetei DM, Khasakhala LI, Mutiso V, Ongecha-Owuor FA, Kokonya DA. Drug use in a rural secondary school in Kenya. Subst Abus. 2010; 31[3]:170–3. doi: 10.1080/08897077.2010.495313 20687005

[35] Ominde BS, Ogeng’o JA, Misiani MK, Kariuki BN. Pattern of stroke in a rural Kenyan hospital. Malawi Med J. 2019;31[1]:50–5. doi: 10.4314/mmj.v31i1.9 31143397 PMC6526339

[36] Omolo OE, Dhadphale M. Prevalence of khat chewers among primary health clinic attenders in Kenya. Acta Psychiatr Scand. 1987;75[3]:318–20. doi: 10.1111/j.1600-0447.1987.tb02795.x 3591416

[37] Kamenderi. Effects of Environment and Parenting Practices on Alcohol Use among Primary School Pupils in Kenya. AJADA. 2020;3.

[38] MacKenzie C, Kiragu K, Odingo G, Yassin R, Shikuku P, Angala P, et al. The feasibility of integrating alcohol risk-reduction counseling into existing VCT services in Kenya. Afr J Drug Alcohol Stud. 2009;8[2]:73–80.

[39] Mutai. Innovations and Opportunities In Social Media For Management Of Drug And Substance Abuse In Selected Informal Settlements of Nairobi County, Kenya. AJADA. 2020;3.

[40] Papas RK, Sidle JE, Martino S, Baliddawa JB, Songole R, Omolo OE, et al. Systematic cultural adaptation of cognitive-behavioral therapy to reduce alcohol use among HIV-infected outpatients in Western Kenya. AIDS Behav. 2010. doi: 10.1007/s10461-009-9647-6 19967441 PMC2949418

[41] Bazzi AR, Yotebieng K, Otticha S, Rota G, Agot K, Ohaga S, et al. PrEP and the syndemic of substance use, violence, and HIV among female and male sex workers: a qualitative study in Kisumu, Kenya. J Int AIDS Soc. 2019;22[4]. 10.1002/jia2.25266 PMC6462807 30983147

[42] Beckerleg S. How “Cool” is Heroin Injection at the Kenya Coast. Drugs Educ Prev Policy. 2004;11[1]:67–77.

[43] Ezard N, Oppenheimer E, Burton A, Schilperoord M, MacDonald D, Adelekan M, et al. Six rapid assessments of alcohol and other substance use in populations displaced by conflict. Confl Health. 2011;5[1]:1–15. doi: 10.1186/1752-1505-5-1 21310092 PMC3050731

[44] Guise A, Dimova M, Ndimbii J, Clark P, Rhodes T. A qualitative analysis of transitions to heroin injection in Kenya: Implications for HIV prevention and harm reduction. Harm Reduct J. 2015;12[1]:1–9. doi: 10.1186/s12954-015-0061-2 26337729 PMC4558953

[45] Guise A, Ndimbii J, Igonya EK, Owiti F, Strathdee SA, Rhodes T. Integrated and differentiated methadone and HIV care for people who use drugs: A qualitative study in Kenya with implications for implementation science. Health Policy Plan. 2019. 2021;34[2]:110–9. doi: 10.1093/heapol/czz002 30789208 PMC6481284

[46] Kibicho J, Campbell JK. Community perspectives of second-generation alcohol misuse and HIV risk in rural Kenya: A gendered syndemic lens. Glob Public Health. 2019;14[12]:1733–43. doi: 10.1080/17441692.2019.1638958 31291832

[47] Mburu G, Ayon S, Tsai AC, Ndimbii J, Wang B, Strathdee S, et al. “Who has ever loved a drug addict? It’s a lie. They think a ‘teja’ is as bad person”: Multiple stigmas faced by women who inject drugs in coastal Kenya. Harm Reduct J. 2018;15[1]:1–8. 29801494 10.1186/s12954-018-0235-9 PMC5970466

[48] Mburu G, Limmer M, Holland P. Role of boyfriends and intimate sexual partners in the initiation and maintenance of injecting drug use among women in coastal Kenya. Addict Behav. 2019;93:20–8. doi: 10.1016/j.addbeh.2019.01.013 30682678

[49] Mburu G, Ayon S, Mahinda S, Kaveh K. Determinants of Women’s Drug Use During Pregnancy: Perspectives from a Qualitative Study. Matern Child Health J. 2020;24[9]:1170–8. doi: 10.1007/s10995-020-02910-w 32754861 PMC7419458

[50] Mburu G, Limmer M, Holland P. HIV risk behaviours among women who inject drugs in coastal Kenya: Findings from secondary analysis of qualitative data. Harm Reduct J. 2019;16[1]:10. doi: 10.1186/s12954-019-0281-y 30728012 PMC6364406

[51] Mburu G, Ndimbii J, Ayon S, Mlewa O, Mbizvo M, Kihara C, et al. Contraceptive Use Among Women Who Inject Drugs: Motivators, Barriers, and Unmet Needs. Women’s Reprod Heal [Internet]. 2018;5[2]:99–116.

[52] Mital S, Miles G, McLellan-Lemal E, Muthui M, Needle R. Heroin shortage in Coastal Kenya: A rapid assessment and qualitative analysis of heroin users’ experiences. *Int J Drug Policy*. 2016;30:91–98. doi: 10.1016/j.drugpo.2015.08.010 26470646 PMC4762754

[53] Muturi N. Alcohol consumption and reproductive health risks in rural Central Kenya. Sex Reprod Healthc. 2014;5[2]:41–6. doi: 10.1016/j.srhc.2014.01.002 24814437

[54] Gender Muturi N. and HIV infection in the context of alcoholism in Kenya. African J AIDS Res. 2015;14[1]:57–65. doi: 10.2989/16085906.2015.1016986 25920984

[55] Muturi N. Community Perspectives on Communication Strategies for Alcohol Abuse Prevention in Rural Central Kenya. J Health Commun. 2016;21[3]:309–17. doi: 10.1080/10810730.2015.1064496 26192335

[56] Ndimbii JN, Guise A, Ayon S, Kalama M, McLean S, Rhodes T. Implementing needle and syringe programmes in Kenya: Changes, opportunities and challenges in HIV prevention. Afr J Drug Alcohol Stud. 2015;14[2]:95–103.

[57] Ndimbii J, Ayon S, Abdulrahman T, Mahinda S, Jeneby F, Armstrong G, et al. Access and utilisation of reproductive, maternal, neonatal and child health services among women who inject drugs in coastal Kenya: Findings from a qualitative study. Sex Reprod Healthc. 2018;18:48–55. doi: 10.1016/j.srhc.2018.10.002 30420087

[58] Njue C, Voeten HACM, Remes P. Disco funerals: A risk situation for HIV infection among youth in Kisumu, Kenya. Aids. 2009;23[4]:505–9. doi: 10.1097/QAD.0b013e32832605d0 19165086 PMC2675523

[59] Njue C., Voeten H.A. & Remes P. Porn video shows, local brew, and transactional sex: HIV risk among youth in Kisumu, Kenya. BMC Public Health 2011; 11:635. doi: 10.1186/1471-2458-11-635 21824393 PMC3199602

[60] Othieno CJ, Obondo A, Mathai M. Improving adherence to ante-retroviral treatment for people with harmful alcohol use in Kariobangi, Kenya through participatory research and action. BMC Public Health. 2012;12[1]:1. doi: 10.1186/1471-2458-12-677 22905910 PMC3575338

[61] Rhodes T, Guise A, Ndimbii J, Strathdee S, Ngugi E, Platt L, et al. Is the promise of methadone Kenya’s solution to managing HIV and addiction? A mixed-method mathematical modelling and qualitative study. BMJ Open. 2015;5[3]. doi: 10.1136/bmjopen-2014-007198 25748417 PMC4360822

[62] Rhodes T. The becoming of methadone in Kenya: How an intervention’s implementation constitutes recovery potential. Soc Sci Med. 2018 Mar;201:71–79. doi: 10.1016/j.socscimed.2018.02.007 29455053 PMC5922264

[63] Ssewanyana D, Mwangala PN, Marsh V, Jao I, van Baar A, Newton CR, et al. Socio-ecological determinants of alcohol, tobacco, and drug use behavior of adolescents in Kilifi County at the Kenyan coast. J Health Psychol. 2018;25[12]. 10.1177/1359105318782594 PMC7116417 29944006

[64] Syvertsen JL, Ohaga S, Agot K, Dimova M, Guise A, Rhodes T, et al. An ethnographic exploration of drug markets in Kisumu, Kenya. Int J Drug Policy. 2016.;30:82–90. doi: 10.1016/j.drugpo.2016.01.001 26838470 PMC4845648

[65] Syvertsen JL, Agot K, Ohaga S, Bazzi AR. You can’t do this job when you are sober: Heroin use among female sex workers and the need for comprehensive drug treatment programming in Kenya. *Drug Alcohol Depend*. 2019;194:495–499. doi: 10.1016/j.drugalcdep.2018.10.019 30529906 PMC6334295

[66] Velloza J, L’Engle K, Mwarogo P, Chokwe J, Magaria L, Sinkele W, et al. Stages and Processes of Change Utilized by Female Sex Workers Participating in an Alcohol-Reduction Intervention in Mombasa, Kenya. Subst Use Misuse. 2015;50[13]:1728–37. doi: 10.3109/10826084.2015.1037397 26595484

[67] Yotebieng KA, Agot K, Rota G, Cohen CR, Syvertsen JL. A qualitative study of substance use during pregnancy: Implications for reproductive healthcare in Western Kenya. Afr J Reprod Health [Internet]. 2016;20[4]:51–9. doi: 10.29063/ajrh2016/v20i4.5 29566319 PMC6076375

[68] Akiyama MJ, Cleland CM, Lizcano JA, Cherutich P, Kurth AE. Prevalence, estimated incidence, risk behaviours, and genotypic distribution of hepatitis C virus among people who inject drugs accessing harm-reduction services in Kenya: a retrospective cohort study. Lancet Infect Dis. 2019;19[11]:1255–63. doi: 10.1016/S1473-3099(19)30264-6 31540840 PMC7099605

[69] Anundo. Prevalence of Depression among Female Injecting Drug Users (FIDUs): Study of a Drop-in Rehabilitation Center in Nairobi County, Kenya. AJADA. 2019;1.

[70] Asiki G, Mohamed SF, Wambui D, Wainana C, Muthuri S, Ramsay M, et al. Sociodemographic and behavioural factors associated with body mass index among men and women in Nairobi slums: AWI-Gen Project. Glob Health Action. 2018;11(sup2). doi: 10.1080/16549716.2018.1470738 29966508 PMC6032012

[71] Åstrøm AN, Ogwell EA. Use of tobacco in Kenya: Sources of information, beliefs and attitudes toward tobacco control measures among primary school students. J Adolesc Heal. 2004;35[3]:234–7. doi: 10.1016/j.jadohealth.2004.02.017 15313506

[72] Atwoli L, Mungla PA, Ndung’u MN, Kinoti KC, Ogot EM. Prevalence of substance use among college students in Eldoret, western Kenya. BMC Psychiatry. 2011;11[1]:34. doi: 10.1186/1471-244X-11-34 21356035 PMC3053226

[73] Ayah R, Joshi MD, Wanjiru R, Njau EK, Otieno CF, Njeru EK, et al. A population-based survey of prevalence of diabetes and correlates in an urban slum community in Nairobi, Kenya. BMC Public Health. 2013;13[1]. 10.1186/1471-2458-13-371 PMC3641964 23601475

[74] Ayaya SO, Esamai FO. Health problems of street children in Eldoret, Kenya. East Afr Med J. 2001;78[12]:624–9. doi: 10.4314/eamj.v78i12.8930 12199442

[75] Balogun O, Koyanagi A, Stickley A, Gilmour S, Shibuya K. Alcohol consumption and psychological distress in adolescents: A multi-country study. J Adolesc Heal. 2014;54[2]:228–34. doi: 10.1016/j.jadohealth.2013.07.034 24064281

[76] Beckerleg S, Telfer M, Sadiq A. A rapid assessment of heroin use in Mombasa, Kenya. Subst Use Misuse. 2006;41[6–7]:1029–44. doi: 10.1080/10826080600667193 16809185

[77] Bengtson AM, L’Engle K, Mwarogo P, King’Ola N. Levels of alcohol use and history of HIV testing among female sex workers in Mombasa, Kenya. AIDS Care—Psychol Socio-Medical Asp AIDS/HIV. 2014;26[12]:1619–24. doi: 10.1080/09540121.2014.938013 25040114 PMC4320941

[78] Budambula V, Matoka C, Ouma J, Ahmed AA, Otieno MF, Were T. Socio-demographic and sexual practices associated with HIV infection in Kenyan injection and non-injection drug users. BMC Public Health [Internet]. 2018;18[1]:193. doi: 10.1186/s12889-018-5100-y 29378631 PMC5789578

[79] Cagle A, Mcgrath C, Richardson BA, Donovan D, Yatich N, Ngomoa R, et al. Alcohol use and immune reconstitution among HIV-infected patients on antiretroviral therapy in Nairobi, Kenya Anthony. AIDS Care. 2018;29[9]:1192–7. 10.1080/09540121.2017.1281881 PMC6009824 28132519

[80] Chersich MF, Bosire W, King’ola N, Temmerman M, Luchters S. Effects of hazardous and harmful alcohol use on HIV incidence and sexual behaviour: A cohort study of Kenyan female sex workers. Global Health. 2014;10[1]:1–11. doi: 10.1186/1744-8603-10-22 24708844 PMC3985581

[81] Christensen DL, Friis H, Mwaniki DL, Kilonzo B, Tetens I, Boit MK, et al. Prevalence of glucose intolerance and associated risk factors in rural and urban populations of different ethnic groups in Kenya. Diabetes Res Clin Pract. 2009;84[3]:303–10. doi: 10.1016/j.diabres.2009.03.007 19361878

[82] Cleland CM, Des Jarlais DC, Perlis TE, Stimson G, Poznyak V, Adelekan M, et al. HIV risk behaviors among female IDUs in developing and transitional countries. BMC Public Health [Internet]. 2007; 7[1]:271. doi: 10.1186/1471-2458-7-271 17908299 PMC2140060

[83] De Menil VP, Knapp M, McDaid D, Njenga FG. Service use, charge, and access to mental healthcare in a private kenyan inpatient setting: The effects of insurance. PLoS One. 2014;9[3]:1–7. 10.1371/journal.pone.0090297 PMC3961252 24651115

[84] Deveau CS, Tengia L, Mutua C, Njoroge S, Dajoh L, Singer B. Utilisation of community-based addiction out-patient treatment programmes in Kenya. Afr J Drug Alcohol Stud. 2010;9[2].

[85] Embleton L, Ayuku D, Atwoli L, Vreeman R, Braitstein P. Knowledge, attitudes, and substance use practices among street children in Western Kenya. Subst Use Misuse. 2012;47[11]:1234–47. doi: 10.3109/10826084.2012.700678 22780841 PMC3749375

[86] Embleton L, Atwoli L, Ayuku D, Braitstein P. The Journey of Addiction: Barriers to and Facilitators of Drug Use Cessation among Street Children and Youths in Western Kenya. PLoS One. 2013;8[1]. doi: 10.1371/journal.pone.0053435 23326428 PMC3541137

[87] Embleton L, Nyandat J, Ayuku D, Sang E, Kamanda A, Ayaya S, et al. Sexual Behavior Among Orphaned Adolescents in Western Kenya: A Comparison of Institutional- and Family-Based Care Settings. J Adolesc Heal. 2017;60[4]:417–24. doi: 10.1016/j.jadohealth.2016.11.015 28110864 PMC5389113

[88] Gathecha GK, Ngaruiya C, Mwai W, Kendagor A, Owondo S, Nyanjau L, et al. Prevalence and predictors of injuries in Kenya: Findings from the national STEPs survey 11 Medical and Health Sciences 1117 Public Health and Health Services. BMC Public Health. 2018;18(Suppl 3). 10.1186/s12889-018-6061-x PMC6219001 30400906

[89] Gichuki JW, Opiyo R, Mugyenyi P, Namusisi K. Healthcare providers’ level of involvement in provision of smoking cessation interventions in public health facilities in Kenya. J Public Health Africa. 2016;6[2]:62–7. 10.4081/jphia.2015.523 PMC5349271 28299144

[90] Giusto A, Green EP, Simmons RA, Ayuku D, Patel P, Puffer ES. A multiple baseline study of a brief alcohol reduction and family engagement intervention for fathers in Kenya. J Consult Clin Psychol. 2020;88[8]:708–25. doi: 10.1037/ccp0000559 32700954 PMC7413306

[91] Goldblatt A, Kwena Z, Lahiff M, Agot K, Minnis A, Prata N, et al. Prevalence and correlates of HIV infection among street boys in Kisumu, Kenya. PLoS One. 2015;10[10]:1–22. 10.1371/journal.pone.0140005 PMC4604137 26461494

[92] Goodman ML, Grouls A, Chen CX, Keiser PH, Gitari S. Adverse Childhood Experiences Predict Alcohol Consumption Patterns Among Kenyan Mothers. Subst Use Misuse. 2017;52[5]:632–8. doi: 10.1080/10826084.2016.1245748 28026977

[93] Hall W, Saunders JB, Babor TF, Aasland OG, Amundsen A, Hodgson R, et al. The structure and correlates of alcohol dependence: WHO collaborative project on the early detection of persons with harmful alcohol consumption—III. Addiction. 1993;88[12]:1627–36. doi: 10.1111/j.1360-0443.1993.tb02037.x 7907509

[94] Harder VS, Musau AM, Musyimi CW, Ndetei DM, Mutiso VN. A randomized clinical trial of mobile phone motivational interviewing for alcohol use problems in Kenya. Addiction. 2020;115[6]:1050–60. doi: 10.1111/add.14903 31782966 PMC8353663

[95] Haregu TN, Oti S, Egondi T, Kyobutungi C. Co-occurrence of behavioral risk factors of common non-communicable diseases among urban slum dwellers in Nairobi, Kenya. Glob Health Action. 2015;8[1]. doi: 10.3402/gha.v8.28697 26385542 PMC4575413

[96] Hulzebosch A, van de Vijver S, Oti SO, Egondi T, Kyobutungi C. Profile of people with hypertension in Nairobi’s slums: A descriptive study. Global Health. 2015;11[1]:1–7. doi: 10.1186/s12992-015-0112-1 26116577 PMC4491223

[97] Jenkins R, Othieno C, Ongeri L, Kiima D, Sifuna P, Kingora J, et al. Alcohol consumption and hazardous drinking in western Kenya-A household survey in a health and demographic surveillance site. BMC Psychiatry. 2015;15[1]:1–10. doi: 10.1186/s12888-015-0603-x 26408143 PMC4582617

[98] Joshi MD, Ayah R, Njau EK, Wanjiru R, Kayima JK, Njeru EK, et al. Prevalence of hypertension and associated cardiovascular risk factors in an urban slum in Nairobi, Kenya: A population-based survey. BMC Public Health. 2014;14[1]:1–10. 25407513 10.1186/1471-2458-14-1177 PMC4246542

[99] Kaai SC, Fong GT, Goma F, Meng G, Ikamari L, Ong’ang’o JR, et al. Identifying factors associated with quit intentions among smokers from two nationally representative samples in Africa: Findings from the ITC Kenya and Zambia Surveys. Prev Med Reports. 2019;15:100951. doi: 10.1016/j.pmedr.2019.100951 31372329 PMC6660566

[100] Kaduka L, Korir A, Oduor CO, Kwasa J, Mbui J, Wabwire S, et al. Stroke distribution patterns and characteristics in Kenya’s leading public health tertiary institutions: Kenyatta National hospital and moi teaching and referral hospital. Cardiovasc J Afr. 2018;29[2]:68–72. doi: 10.5830/CVJA-2017-046 29745965 PMC6008906

[101] Kamau JW, Omigbodun OO, Bella-Awusah T, Adedokun B. Who seeks child and adolescent mental health care in Kenya? A descriptive clinic profile at a tertiary referral facility. Child Adolesc Psychiatry Ment Health. 2017;11[1]:14. 28293286 10.1186/s13034-017-0151-x PMC5348803

[102] Kamenderi M, Muteti J, Okioma V, Nyamongo I, Kimani S, Kanana F, et al. Status of Drugs and Substance Use among Secondary School Students in Kenya. AJADA. 2019; 1

[103] Kamenderi M, Muteti J, Okioma V, Kimani S, Kanana F, Kahiu C. Status of Drugs and Substance Abuse among the General Population in Kenya. AJADA. 2019;1.

[104] Kamenderi M, Muteti J, Okioma V, Kimani S. Prevalence and Predictors of Multiple Substance Use Disorders in Kenya. AJADA. 2019;2.

[105] Kamotho C, Ogola EO, Joshi M, Gikonyo D. Cardiovascular risk factor profile of Black Africans undergoing coronary angiography. East Afr Med J. 2004;81[2]:82–6. doi: 10.4314/eamj.v81i2.9130 15125091

[106] Kaplan M, Carriker L, Waldron I. Gender differences in tobacco use in Kenya. Soc Sci Med. 1990;30[3]:305–10. doi: 10.1016/0277-9536(90)90186-v 2309128

[107] Kendagor A, Gathecha G, Ntakuka MW, Nyakundi P, Gathere S, Kiptui D, et al. Prevalence and determinants of heavy episodic drinking among adults in Kenya: Analysis of the STEPwise survey, 2015. BMC Public Health. 2018;18(Suppl 3). doi: 10.1186/s12889-018-6057-6 30400910 PMC6219062

[108] Khasakhala LI, Ndetei DM, Mathai M, Harder V. Major depressive disorder in a Kenyan youth sample: Relationship with parenting behavior and parental psychiatric disorders. Ann Gen Psychiatry [Internet]. 2013;12[1]:1–10. 23663452 10.1186/1744-859X-12-15 PMC3660220

[109] Khasakhala LI, Ndetei DM, Mathai M. Suicidal behaviour among youths associated with psychopathology in both parents and youths attending outpatient psychiatric clinic in Kenya. Ann Gen Psychiatry. 2013;12[1]. doi: 10.1186/1744-859X-12-13 23622559 PMC3644274

[110] Kiburi SK, Molebatsi K, Obondo A, Kuria MW. Adverse childhood experiences among patients with substance use disorders at a referral psychiatric hospital in Kenya. BMC Psychiatry. 2018;18[1]. 10.1186/s12888-018-1780-1 PMC6007077 29914409

[111] Kimando MW, Otieno FCF, Ogola EN, Mutai K. Adequacy of control of cardiovascular risk factors in ambulatory patients with type 2 diabetes attending diabetes out-patients clinic at a county hospital, Kenya. BMC Endocr Disord. 2017;17[1]:1–11. 29191193 10.1186/s12902-017-0223-1 PMC5709860

[112] Kimani S, Mirie W, Chege M, Okube OT, Muniu S. Association of lifestyle modification and pharmacological adherence on blood pressure control among patients with hypertension at Kenyatta National Hospital, Kenya: A cross-sectional study. BMJ Open. 2019;9[1]. doi: 10.1136/bmjopen-2018-023995 30782721 PMC6340423

[113] Kimbui E, Kuria M, Yator O, Kumar M. A cross-sectional study of depression with comorbid substance use dependency in pregnant adolescents from an informal settlement of Nairobi: Drawing implications for treatment and prevention work. Ann Gen Psychiatry.2018;17[1]:1–15. doi: 10.1186/s12991-018-0222-2 30598688 PMC6300883

[114] Kinoti KE, Jason LA, Harper GW. Determinants of Alcohol, Khat, and Bhang Use in Rural Kenya. Afr J Drug Alcohol Stud. 2011;10[2]:107–18. 23348827 PMC3551616

[115] Kinyanjui DWC, Atwoli L. Substance use among inmates at the Eldoret prison in Western Kenya. BMC Psychiatry. 2013;13. doi: 10.1186/1471-244X-13-53 23406288 PMC3576302

[116] Komu P, Dimba EAO, Macigo FG, Ogwell AEO. Cigarette smoking and oral health among healthcare students. East Afr Med J. 2009;86[4]:178–82. doi: 10.4314/eamj.v86i4.46948 20085002

[117] Korhonen C, Kimani M, Wahome E, Otieno F, Okall D, Bailey RC, et al. Depressive symptoms and problematic alcohol and other substance use in 1476 gay, bisexual, and other MSM at three research sites in Kenya. Aids. 2018;32[11]:1507–15. doi: 10.1097/QAD.0000000000001847 29734218 PMC6150184

[118] Kunzweiler CP, Bailey RC, Okall DO, Graham SM, Mehta SD, Otieno FO. Factors Associated with Prevalent HIV Infection among Kenyan MSM: The Anza Mapema Study. J Acquir Immune Defic Syndr. 2017;76[3]:241–9. doi: 10.1097/QAI.0000000000001512 28746167

[119] Kunzweiler CP, Bailey RC, Okall DO, Graham SM, Mehta SD, Otieno FO. Depressive Symptoms, Alcohol and Drug Use, and Physical and Sexual Abuse Among Men Who Have Sex with Men in Kisumu, Kenya: The Anza Mapema Study. AIDS Behav. 2018;22[5]:1517–29. doi: 10.1007/s10461-017-1941-0 29079946

[120] Kuria MW, Ndetei DM, Obot IS, Khasakhala LI, Bagaka BM, Mbugua MN, et al. The Association between Alcohol Dependence and Depression before and after Treatment for Alcohol Dependence. ISRN Psychiatry. 2012;1–6. 10.5402/2012/482802 PMC3658562 23738204

[121] Kurth AE, Cleland CM, Des Jarlais DC, Musyoki H, Lizcano JA, Chhun N, et al. HIV prevalence, estimated incidence, and risk behaviors among people who inject drugs in Kenya. J Acquir Immune Defic Syndr. 2015;70[4]:420–7. doi: 10.1097/QAI.0000000000000769 26226249 PMC4624615

[122] Kurui DK, Ogoncho IM. Prevalence of Substance Abuse among Students in Medical Training Colleges in South Nyanza Region, Kenya. AJADA. 2019;2.

[123] Kurui DK, Ogoncho IM. Determinants of Alcohol Use by Students in Medical Training Colleges in South Nyanza Region, Kenya. AJADA. 2020;3.

[124] Kwamanga DHO, Odhiambo JA, Gicheha C. Tobacco consumption among primary school teachers in nairobi. East Afr Med J. 2001;78[3]:119–23. doi: 10.4314/eamj.v78i3.9075 12002049

[125] Kwamanga DHO, Odhiambo JA, Amukoye EI. Prevalence and risk factors of smoking among secondary school students in Nairobi. East Afr Med J. 2003;80[4]:207–12. doi: 10.4314/eamj.v80i4.8644 12918805

[126] Kwobah E, Epstein S, Mwangi A, Litzelman D, Atwoli L. PREVALENCE of psychiatric morbidity in a community sample in Western Kenya. BMC Psychiatry.2017;17[1]. 10.1186/s12888-017-1202-9 PMC5242046 28100210

[127] L’Engle KL, Mwarogo P, Kingola N, Sinkele W, Weiner DH. A randomized controlled trial of a brief intervention to reduce alcohol use among female sex workers in Mombasa, Kenya. J Acquir Immune Defic Syndr. 2014;67[4]:446–53. doi: 10.1097/QAI.0000000000000335 25197826

[128] Lo TQ, Oeltmann JE, Odhiambo FO, Beynon C, Pevzner E, Cain KP, et al. Alcohol use, drunkenness and tobacco smoking in rural western Kenya. Trop Med Int Heal. 2013;18[4]:506–15. doi: 10.1111/tmi.12066 23489316 PMC8961680

[129] Luchters S, Geibel S, Syengo M, Lango D, King’Ola N, Temmerman M, et al. Use of AUDIT, and measures of drinking frequency and patterns to detect associations between alcohol and sexual behaviour in male sex workers in Kenya. BMC Public Health. 2011;11:1–8. 21609499 10.1186/1471-2458-11-384 PMC3128017

[130] Lukandu OM, Koech LS, Kiarie PN. Oral Lesions Induced by Chronic Khat Use Consist Essentially of Thickened Hyperkeratinized Epithelium. Int J Dent. 2015;104812. doi: 10.1155/2015/104812 26491446 PMC4600501

[131] Magati P, Drope J, Mureithi L, Lencucha R. Socio-economic and demographic determinants of tobacco use in Kenya: Findings from the Kenya demographic and health survey 2014. Pan Afr Med J. 2018;30:1–10. doi: 10.11604/pamj.2018.30.166.14771 30455795 PMC6235476

[132] Maina RW, Obondo AA, Kuria MW, Donovan DM. Substance use literacy: Implications for HIV medication adherence and addiction severity among substance users. Afr J Drug Alcohol Stud. 2015;14[2]:137–51.

[133] Mannik JR, Figol A, Churchill V, Aw J, Francis S, Karino E, et al. Innovation in cardiovascular risk using a novel mHealth tool in rural Kenya. J Innov Heal Inf. 2018;25[3]:176–82. 10.14236/jhi.v25i3.1012 30398461

[134] Mburu JW, Kingwara L, Ester M, Andrew N. Prognostic factors among TB and TB/DM comorbidity among patients on short course regimen within Nairobi and Kiambu counties in Kenya. J Clin Tuberc Other Mycobact Dis. 2018;12:9–13. doi: 10.1016/j.jctube.2018.04.005 31720392 PMC6830184

[135] Medley A, Seth P, Pathak S, Howard AA, Deluca N, Matiko E, et al. Alcohol use and its association with HIV risk behaviors among a cohort of patients attending HIV clinical care in Tanzania, Kenya, and Namibia. AIDS Care—Psychol Socio-Medical Asp AIDS/HIV. 2014;26[10]:1288–97. 10.1080/09540121.2014.911809 PMC4664067 24773163

[136] Menach P, Oburra HO, Patel A. Cigarette Smoking and Alcohol Ingestion as Risk Factors for Laryngeal Squamous Cell Carcinoma at Kenyatta National Hospital, Kenya. Clin Med Insights Ear, Nose Throat. 2012;5 doi: 10.4137/CMENT.S8610 24179405 PMC3791957

[137] Menya D, Kigen N, Oduor M, Maina SK, Some F, Chumba D, et al. Traditional and commercial alcohols and esophageal cancer risk in Kenya. Int J Cancer. 2019;144[3]:459–69. doi: 10.1002/ijc.31804 30117158 PMC6294681

[138] Mkuu RS, Barry AE, Montiel Ishino FA, Amuta AO. Examining characteristics of recorded and unrecorded alcohol consumers in Kenya. BMC Public Health. 2018;18[1]:1–8. doi: 10.1186/s12889-018-5960-1 30139353 PMC6108107

[139] Mohamed SF, Mutua MK, Wamai R, Wekesah F, Haregu T, Juma P, et al. Prevalence, awareness, treatment and control of hypertension and their determinants: Results from a national survey in Kenya. BMC Public Health. 2018;18(Suppl 3). 10.1186/s12889-018-6052-y PMC6219055 30400858

[140] Mokaya AG, Mutiso V, Musau A, Tele A, Kombe Y, Ng’ang’a Z, et al. Substance Use among a Sample of Healthcare Workers in Kenya: A Cross-Sectional Study. J Psychoactive Drugs. 2016;48[4]:310–9. doi: 10.1080/02791072.2016.1211352 27485987 PMC5020342

[141] Moscoe E, Agot K, Thirumurthy H. Effect of a Prize-Linked Savings Intervention on Savings and Healthy Behaviors Among Men in Kenya: A Randomized Clinical Trial. JAMA Netw open. 2019;2[9] doi: 10.1001/jamanetworkopen.2019.11162 31517964 PMC6745050

[142] Mundan V, Muiva M, Kimani S. Physiological, Behavioral, and Dietary Characteristics Associated with Hypertension among Kenyan Defence Forces. ISRN Prev Med. 2013;2013:1–8. 10.5402/2013/740143 PMC4062863 24977096

[143] Mungai D, Midigo R. Social and cultural determinants of health; understanding the persisting Alcohol Use Disorder (AUD) in the rural populations in central Kenya. AIMS Public Heal. 2019;6[4]:600–14. 10.3934/publichealth.2019.4.600 PMC6940580 31909079

[144] Muraguri N, Tun W, Okal J, Broz D, Fisher Raymond H, Kellogg T, et al. HIV and STI prevalence and risk factors among male sex workers and other men who have sex with men in nairobi, kenya. J Acquir Immune Defic Syndr [Internet]. 2015;68[1]:91–6. doi: 10.1097/QAI.0000000000000368 25501346 PMC4973514

[145] Muriungi SK, Ndetei DM. Effectiveness of psycho-education on depression, hopelessness, suicidality, anxiety and substance use among basic diploma students at Kenya Medical Training College. South African J Psychiatry. 2013;19[2]:41–50.

[146] Muthumbi E, Lowe BS, Muyodi C, Getambu E, Gleeson F, Scott JAG. Risk factors for community-acquired pneumonia among adults in Kenya: a case–control study. Pneumonia. 2017;9[1]:1–9. doi: 10.1186/s41479-017-0041-2 29209590 PMC5702239

[147] Mutiso VN, Musyimi CW, Krolinski P, Neher CM, Musau AM, Tele A, et al. Relationship between Bullying, Substance Use, Psychiatric Disorders, and Social Problems in a Sample of Kenyan Secondary Schools. Prev Sci.2019;20[4]:544–54. doi: 10.1007/s11121-019-01014-4 30993591

[148] Muture BN, Keraka MN, Kimuu PK, Kabiru EW, Ombeka VO, Oguya F. Factors associated with default from treatment among tuberculosis patients in nairobi province, Kenya: A case control study. BMC Public Health.2011;11. doi: 10.1186/1471-2458-11-696 21906291 PMC3224095

[149] Mwangi C, Karanja S, Gachohi J, Wanjihia V, Ngang’A Z. Depression, injecting drug use, and risky sexual behavior syndemic among women who inject drugs in Kenya: A cross-sectional survey. Harm Reduct J. 2019;16[1]:1–11. 31146748 10.1186/s12954-019-0307-5 PMC6543607

[150] Nall A, Chenneville T, Rodriguez LM, O’Brien JL. Factors affecting hiv testing among youth in kenya. Int J Environ Res Public Health. 2019;16[8]:1–14. doi: 10.3390/ijerph16081450 31022872 PMC6517959

[151] Ndegwa S, Waiyaki W. Effects of Parental Abandonment and Strife on Youth Drug Use. AJADA. 2020;3.

[152] Ndetei D, Khasakhala L, Ong’echa F, Kokonya D, Mutiso V, Kuria M, et al. A study of drug use in five urban centres in Kenya. Afr J Drug Alcohol Stud. 2009;7[1].

[153] Ndetei DM, Khasakhala LI, Mutiso V, Ongecha-Owuor FA, Kokonya DA. Patterns of drug abuse in public secondary schools in Kenya. Subst Abus. 2009;30[1]:69–78. doi: 10.1080/08897070802606436 19197783

[154] Ndetei DM, Khasakhala LI, Mutiso V, Ongecha-Owuor FA, Kokonya DA. Psychosocial and health aspects of drug use by students in public secondary schools in Nairobi, Kenya. Subst Abus. 2009;30[1]:61–8. doi: 10.1080/08897070802606410 19197782

[155] Ndetei DM, Khasakhala L, Meneghini L, Aillon JL. The_relationship_between_schizo-affective and mood disorders in patients. 2013;(March):110–7. 10.4314/ajpsy.v16i2.14 23595530

[156] Ndugwa RP, Kabiru CW, Cleland J, Beguy D, Egondi T, Zulu EM, et al. Adolescent problem behavior in nairobi’s informal settlements: Applying problem behavior theory in Sub-Saharan Africa. J Urban Heal. 2011;88(SUPPL. 2):298–317. doi: 10.1007/s11524-010-9462-4 20499192 PMC3132234

[157] Ng’Ang’A A, Nyangasi M, Nkonge NG, Gathitu E, Kibachio J, Gichangi P, et al. Predictors of cervical cancer screening among Kenyan women: Results of a nested case-control study in a nationally representative survey. BMC Public Health.2018;18(Suppl 3). 10.1186/s12889-018-6054-9 PMC6219012 30400916

[158] Ngaruiya C, Abubakar H, Kiptui D, Kendagor A, Ntakuka MW, Nyakundi P, et al. Tobacco use and its determinants in the 2015 Kenya WHO STEPS survey. BMC Public Health. 2018;18(Suppl 3):14–6. doi: 10.1186/s12889-018-6058-5 30400915 PMC6219013

[159] Nguchu HK, Joshi MD, Otieno CF. Acute coronary syndromes amongst type 2 diabetics with ischaemic electrocardiograms presenting to Accident and Emergency department of a Kenyan Tertiary Institution. East Afr Med J. 2009;86(10):463–8. doi: 10.4314/eamj.v86i10.54972 21650069

[160] Ngure J, Omulema B, Chepchieng M. Level of risk in substance use among undergraduate students in Kenya: Implications for prevention intervention. AJADA. 2019;1.

[161] Nielsen MFJ, Resnick CA, Acuda SW. Alcoholism Among Outpatients of a Rural District General Hospital in Kenya. Br J Addict. 1989;84(11):1343–51. doi: 10.1111/j.1360-0443.1989.tb00736.x 2597810

[162] Njoroge A, Guthrie BL, Bosire R, Wener M, Kiarie J, Farquhar C. Low HDL-cholesterol among HIV-1 infected and HIV-1 uninfected individuals in Nairobi, Kenya. Lipids Health Dis. 2017;16[1]:110. doi: 10.1186/s12944-017-0503-9 28599673 PMC5466788

[163] Ogwell AEO, Aström AN, Haugejorden O. Socio-demographic factors of pupils who use tobacco in randomly-selected primary schools in Nairobi Province, Kenya. East Afr Med J. 2003;80: 235–41. doi: 10.4314/eamj.v80i5.8693 16167738

[164] Okal J, Geibel S, Muraguri N, Musyoki H, Tun W, Broz D, et al. Estimates of the Size of key populations at risk for HIV infection: Men who have sex with men, female sex workers and injecting drug users in Nairobi, Kenya. Sex Transm Infect. 2013;89[5]:366–71. doi: 10.1136/sextrans-2013-051071 23761166 PMC4784698

[165] Olack B, Wabwire-Mangen F, Smeeth L, Montgomery JM, Kiwanuka N, Breiman RF. Risk factors of hypertension among adults aged 35–64 years living in an urban slum Nairobi, Kenya. BMC Public Health. 2015;15[1]:1–9. 26679701 10.1186/s12889-015-2610-8 PMC4683777

[166] Ongeri L, Kirui F, Muniu E, Manduku V, Kirumbi L, Atwoli L, et al. Khat use and psychotic symptoms in a rural Khat growing population in Kenya: A household survey. BMC Psychiatry. 2019;19[1]:1–10. 31064338 10.1186/s12888-019-2118-3 PMC6505064

[167] Onsomu EO, Abuya BA, Okech IN, Rosen DL, Duren-Winfield V, Simmons AC. Association Between Domestic Violence and HIV Serostatus Among Married and Formerly Married Women in Kenya. Health Care Women Int. 2015;36[2]:205–28. doi: 10.1080/07399332.2014.943840 25127397 PMC4312516

[168] Othieno CJ, Kathuku DM, Ndetei DM. Substance abuse in outpatients attending rural and urban health centres in Kenya. East Afr Med J. 2000;77[11]:592–5. doi: 10.4314/eamj.v77i11.46728 12862104

[169] Othieno CJ, Okoth RO, Peltzer K, Pengpid S, Malla LO. Depression among university students in Kenya: Prevalence and sociodemographic correlates. J Affect Disord.2014;165:120–5. doi: 10.1016/j.jad.2014.04.070 24882188

[170] Othieno CJ, Okoth R, Peltzer K, Pengpid S, Malla LO. Risky HIV sexual behaviour and depression among University of Nairobi students. Ann Gen Psychiatry. 2015;14[1]:1–8. 25873984 10.1186/s12991-015-0054-2 PMC4396741

[171] Othieno CJ, Okoth R, Peltzer K, Pengpid S, Malla LO. Traumatic experiences, posttraumatic stress symptoms, depression, and health-risk behavior in relation to injury among University of Nairobi students in Kenya. Int J Psychiatry Med. 2015;50[3]:299–316. doi: 10.1177/0091217415610310 26561275

[172] Owuor HA, Karega M. Relationship Between Attachment Styles And Risk For Problematic Drug Use Among Undergraduate Students In Selected Universities In Kenya. AJADA. 2019;2.

[173] Oyaro M, Wylie J, Chen CY, Ondondo RO, Kramvis A. Human immunodeficiency virus infection predictors and genetic diversity of hepatitis B virus and hepatitis C virus co-infections among drug users in three major Kenyan cities. South Afr J HIV Med. 2018;19[1]:1–9. doi: 10.4102/sajhivmed.v19i1.737 29707384 PMC5913779

[174] Pack AP, L’Engle K, Mwarogo P, Kingola N. Intimate partner violence against female sex workers in Mombasa, Kenya. Cult Heal Sex.2014;16[3]:217–30. 10.1080/13691058.2013.857046 24329103

[175] Papas RK, Sidle JE, Gakinya BN, Baliddawa JB, Martino S, Mwaniki MM, et al. Treatment outcomes of a stage 1 cognitive-behavioral trial to reduce alcohol use among human immunodeficiency virus-infected out-patients in western Kenya. Addiction. 2011;106[12]:2156–66. doi: 10.1111/j.1360-0443.2011.03518.x 21631622 PMC3208780

[176] Papas RK, Gakinya BN, Mwaniki MM, Keter AK, Lee H, Loxley MP, et al. Associations Between the Phosphatidylethanol Alcohol Biomarker and Self-Reported Alcohol Use in a Sample of HIV-Infected Outpatient Drinkers in Western Kenya. Alcohol Clin Exp Res.2016;40[8]:1779–87. doi: 10.1111/acer.13132 27426424 PMC4961598

[177] Papas RK, Gakinya BN, Mwaniki MM, Lee H, Kiarie SW, Martino S, et al. Rates and Covariates of Recent Sexual and Physical Violence Against HIV-Infected Outpatient Drinkers in Western Kenya. AIDS Behav. 2017;21[8]:2243–52. doi: 10.1007/s10461-017-1684-y 28097617 PMC5513791

[178] Parcesepe AM, L’Engle KL, Martin SL, Green S, Suchindran C, Mwarogo P. Early sex work initiation and condom use among alcohol-using female sex workers in Mombasa, Kenya: a cross-sectional analysis. Sex Transm Infect. 2016;92[8]:593–598. doi: 10.1136/sextrans-2016-052549 27217378 PMC5215884

[179] Patel K, Wakhisi J, Mining S, Mwangi A, Patel R. Esophageal Cancer, the Topmost Cancer at MTRH in the Rift Valley, Kenya, and Its Potential Risk Factors. ISRN Oncol. 2013;2013:1–9. 10.1155/2013/503249 PMC3893746 24490085

[180] Peltzer K. Prevalence and correlates of substance use among school children in six African countries. Int J Psychol [Internet]. 2009;44[5]:378–86. doi: 10.1080/00207590802511742 22029616

[181] Peltzer K. Early smoking initiation and associated factors among in-school male and female adolescents in seven African countries. Afr Health Sci. 2011;11[3]:320–8. 22275919 PMC3261015

[182] Pengpid S, Peltzer K. Alcohol use among adults in Kenya: Results from the National Non-Communicable Diseases Risk Factor survey, 2015. J Psychol Africa. 2019;29[1]:49–53.

[183] Perl R, Murukutla N, Occleston J, Bayly M, Lien M, Wakefield M, et al. Responses to antismoking radio and television advertisements among adult smokers and non-smokers across Africa: Message-testing results from Senegal, Nigeria and Kenya. Tob Control. 2015;24[6]:601–8. doi: 10.1136/tobaccocontrol-2014-051682 25184685

[184] Ploubidis GB, Mathenge W, De Stavola B, Grundy E, Foster A, Kuper H. Socioeconomic position and later life prevalence of hypertension, diabetes and visual impairment in Nakuru, Kenya. Int J Public Health. 2013;58[1]:133–41. doi: 10.1007/s00038-012-0389-2 22814479

[185] Roth EA, Benoit C, Jansson M, Hallsgrimdottir H. Public Drinking Venues as Risk Environments: Commercial Sex, Alcohol and Violence in a Large Informal Settlement in Nairobi, Kenya. Hum Ecol [Internet]. 2017;45[2]:277–83. doi: 10.1007/s10745-017-9897-2 28983133 PMC5624530

[186] Rudatsikira E, Ogwell AE, Siziya S, Muula AS. Prevalence of sexual intercourse among school-going adolescents in Coast Province, Kenya. Tanzan Health Res Bull. 2007;9[3]:159–63. doi: 10.4314/thrb.v9i3.14322 18087892

[187] Sanders EJ, Graham SM, Okuku HS, Van Der Elst EM, Muhaari A, Davies A, et al. HIV-1 infection in high risk men who have sex with men in Mombasa, Kenya. Aids. 2007;21[18]:2513–20. doi: 10.1097/QAD.0b013e3282f2704a 18025888

[188] Saunders JB, Aasland OG, Amundsen A, Grant M. Alcohol consumption and related problems among primary health care patients: WHO Collaborative Project on Early Detection of Persons with Harmful Alcohol Consumption—I. Addiction. 1993;88[3]:349–62. doi: 10.1111/j.1360-0443.1993.tb00822.x 8461852

[189] Secor AM, Wahome E, Micheni M, Rao D, Simoni JM, Sanders EJ, et al. Depression, substance abuse and stigma among men who have sex with men in coastal Kenya. Aids. 2015;29[0 3]:S251–9. 26562814 10.1097/QAD.0000000000000846 PMC4706380

[190] Syvertsen JL, Agot K, Ohaga S, Strathdee SA, Camlin CS, Omanga E, et al. Evidence of injection drug use in Kisumu, Kenya: Implications for HIV prevention. Drug Alcohol Depend. 2015;151:262–6. doi: 10.1016/j.drugalcdep.2015.02.037 25861945 PMC4447587

[191] Shaffer DN, Njeri R, Justice AC, Odero WW, Tierney WM. Alcohol abuse among patients with and without HIV infection attending public clinics in western Kenya. East Afr Med J. 2004;81[11]:594–8. 15868970

[192] Takahashi R, Wilunda C, Magutah K, Mwaura-Tenambergen W, Wilunda B, Perngparn U. Correlates of alcohol consumption in rural western Kenya: A cross-sectional study. BMC Psychiatry [Internet]. 2017;17[1]:175. doi: 10.1186/s12888-017-1344-9 28486959 PMC5424353

[193] Takahashi R, Wilunda C, Magutah K, Mwaura-Tenambergen W, Atwoli L, Perngparn U. Evaluation of alcohol screening and community-based brief interventions in rural western Kenya: A quasi-experimental study. Alcohol Alcohol. 2018;53[1]:121–8. doi: 10.1093/alcalc/agx083 29087434

[194] Tang S, Bishwajit G, Luba TR, Yaya S. Prevalence of smoking among men in Ethiopia and Kenya: A cross-sectional study. Int J Environ Res Public Health. 2018;15[6]. doi: 10.3390/ijerph15061232 29891795 PMC6025624

[195] Tegang SP, Abdallah S, Emukule G, Luchters S, Kingola N, Barasa M, et al. Concurrent sexual and substance-use risk behaviors. 2010;7[4]:10–6. doi: 10.1080/17290376.2010.9724972 21409306

[196] Tsuei SH, Clair V, Mutiso V, Musau A, Tele A. Factors Influencing Lay and Professional Health Workers ‘ Self-Efficacy in Identification and. 2019;15[4]:766–81. PMC6761831 31558889

[197] Tun W, Sheehy M, Broz D, Okal J, Muraguri N, Raymond HF, et al. HIV and STI Prevalence and Injection Behaviors Among People Who Inject Drugs in Nairobi: Results from a 2011 Bio-behavioral Study Using Respondent-Driven Sampling. AIDS Behav. 2015;19[1]:24–35. doi: 10.1007/s10461-014-0936-3 25398417 PMC4352193

[198] Wekesah FM, Nyanjau L, Kibachio J, Mutua MK, Mohamed SF, Grobbee DE, et al. Individual and household level factors associated with presence of multiple non-communicable disease risk factors in Kenyan adults. BMC Public Health. 2018;18(Suppl 3). doi: 10.1186/s12889-018-6055-8 30400905 PMC6219015

[199] Were T, Wesongah JO, Munde E, Ouma C, Kahiga TM, Ongecha-Owuor F, et al. Clinical chemistry profiles in injection heroin users from Coastal Region, Kenya. BMC Clin Pathol. 2014;14[1]:1–9. 25057262 10.1186/1472-6890-14-32 PMC4107560

[200] White D, Wilson KS, Masese LN, Wanje G, Jaoko W, Mandaliya K, et al. Alcohol use and associations with biological markers and self-reported indicators of unprotected sex in human immunodeficiency virus-positive female sex workers in Mombasa, Kenya. Sex Transm Dis. 2016;43[10]:642–7. doi: 10.1097/OLQ.0000000000000502 27631360 PMC5026390

[201] Widmann M, Warsame AH, Mikulica J, von Beust J, Isse MM, Ndetei D, et al. Khat use, PTSD and psychotic symptoms among somali refugees in Nairobi—a pilot study. Front Public Heal. 2014;2:1–10. doi: 10.3389/fpubh.2014.00071 25072043 PMC4075009

[202] Widmann M, Apondi B, Musau A, Warsame AH, Isse M, Mutiso V, et al. Comorbid psychopathology and everyday functioning in a brief intervention study to reduce khat use among Somalis living in Kenya: description of baseline multimorbidity, its effects of intervention and its moderation effects on substance use. Soc Psychiatry Psychiatr Epidemiol. 2017;52[11]:1425–34. doi: 10.1007/s00127-017-1368-y 28321455

[203] Wilson KS, Deya R, Masese L, Simoni JM, Stoep A Vander, Shafi J, et al. Prevalence and correlates of intimate partner violence in HIV-positive women engaged in transactional sex in Mombasa, Kenya. Int J STD AIDS. 2016;27[13]:1194–203. doi: 10.1177/0956462415611514 26464502 PMC4829471

[204] Winston SE, Chirchir AK, Muthoni LN, Ayuku D, Koech J, Nyandiko W, et al. Prevalence of sexually transmitted infections including HIV in street-connected adolescents in western Kenya. Sex Transm Infect. 2015;91[5]:353–9. doi: 10.1136/sextrans-2014-051797 25714102 PMC4518741

[205] Winter SC, Obara LM, McMahon S. Intimate partner violence: A key correlate of women’s physical and mental health in informal settlements in Nairobi, Kenya. PLoS One. 2020;15[4]:1–18. doi: 10.1371/journal.pone.0230894 32240207 PMC7117691

[206] Woldu DO, Haile ZT, Howard S, Walther C, Otieno A, Lado B. Association between substance use and concurrent sexual relationships among urban slum dwellers in Nairobi, Kenya. AIDS Care—Psychol Socio-Medical Asp AIDS/HIV. 2019;31[11]:1454–60. doi: 10.1080/09540121.2019.1595519 30894010

[207] United Nations Office on Drugs and Crime. New psychoactive substances.2020. https://www.unodc.org/documents/scientific/NPS-Leaflet_WEB_2020.pdf. Accessed 12 June 2021

[208] UNODC World Drug Report 2020: Global drug use rising; while COVID-19 has far reaching impact on global drug markets. 2020. https://www.unodc.org/unodc/press/releases/2020/June/media-advisory—global-launch-of-the-2020-world-drug-report.html

[209] Yapa HM, Bärnighausen T. Implementation science in resource-poor countries and communities [Internet]. Vol. 13, Implementation Science. 2018;13:1–13. doi: 10.1186/s13012-018-0847-1 30587195 PMC6307212

[210] World Health Organisation 2017. ‘ Best Buys ‘ and Other Recommended Interventions for the Prevention and Control of Noncommunicable Diseases; “the updated Appendix 3 of the WHO Global NCD Action Plan 2013–2020. https://www.who.int/ncds/management/WHO_Appendix_BestBuys.pdf. Accessed 12 June 2021

[211] Green EP, Lai Y, Pearson N, Rajasekharan S, Rauws M, Joerin A, et al. Expanding access to perinatal depression treatment in Kenya through automated psychological support: Development and usability study. JMIR Form Res. 2020;4[10]. doi: 10.2196/17895 33016883 PMC7573703

[212] Business Today. Kenya leads Africa in smartphone usage [Internet]. 2020. https://businesstoday.co.ke/kenya-leads-africa-smartphone-usage/. Accessed 12 June 2021.

[213] Wang D, Wang Y, Wang Y, Li R, Zhou C. Impact of physical exercise on substance use disorders: A meta-analysis. PLoS One. 2014;9[10]:110728. doi: 10.1371/journal.pone.0110728 25330437 PMC4199732

[214] Li W, Howard MO, Garland EL, McGovern P, Lazar M. Mindfulness treatment for substance misuse: A systematic review and meta-analysis. J Subst Abuse Treat. 2017;75:62–96. doi: 10.1016/j.jsat.2017.01.008 28153483

