# Supplementary material for: A systematic review of substance use and substance use disorder research in Kenya
Source: PLoS One. 2022 Jun 9;17(6):e0269340. doi: 10.1371/journal.pone.0269340 (PMC9186181; doi:10.1371/journal.pone.0269340)
Supplement: S1 File — (PDF) [file pone.0269340.s002.pdf]

Thursday, August 20, 2020 12:54:35 PM

#	Query	Limiters/Expanders	Last Run Via	Results
S4	(Kenya OR eldoret OR nairobi OR mombasa) AND (S1 AND S2 AND S3)	Expanders - Apply equivalent subjects Search modes - Boolean/Phrase	Interface - EBSCOhost Research Databases Search Screen - Advanced Search Database - APA PsycInfo	173
S3	Kenya OR eldoret OR nairobi OR mombasa	Expanders - Apply equivalent subjects Search modes - Boolean/Phrase	Interface - EBSCOhost Research Databases Search Screen - Advanced Search Database - APA PsycInfo	4,551
S2	Prevalence OR "pattern of use" OR treatment OR prevention OR intervention	Expanders - Apply equivalent subjects Search modes - Boolean/Phrase	Interface - EBSCOhost Research Databases Search Screen - Advanced Search Database - APA PsycInfo	1,302,607
S1	"Substance use disorder" OR "substance abuse" OR "substance dependence" OR addiction OR addicts OR "alcohol use disorders" OR "alcohol abuse" OR "alcohol dependence" OR "alcohol addiction" OR "tobacco use" OR cigarette OR smoking OR "nicotine addiction" OR "cannabis use" OR marijuana OR bhang OR miraa OR khat OR shisha OR opioid OR "heroin users" OR "injecting drug users" OR "people who inject drugs" OR "people who use drugs"	Expanders - Apply equivalent subjects Search modes - Boolean/Phrase	Interface - EBSCOhost Research Databases Search Screen - Advanced Search Database - APA PsycInfo	264,483
