# Supplementary material for: A systematic review of substance use and substance use disorder research in Kenya
Source: PLoS One. 2022 Jun 9;17(6):e0269340. doi: 10.1371/journal.pone.0269340 (PMC9186181; doi:10.1371/journal.pone.0269340)
Supplement: Attachment — Submitted filename: PONE-D-22-00681.pdf [file pone.0269340.s003.pdf]

Manuscript Number: PONE-D-22-00681

Title: A systematic review of substance use and substance use disorder research in Kenya

1. Recommendation: Major Revision
2. Is the manuscript technically sound, and do the data support the conclusions? Partly
3. Has the statistical analysis been performed appropriately and rigorously? N/A
4. Does the manuscript adhere to the PLOS Data Policy? Yes
5. Is the manuscript presented in an intelligible fashion and written in standard English? Yes
6. Review Comments to the Author: See below
7. Would you like your identity revealed to the authors of this submission? No
8. Do you have any potentially competing interests? None

Summary
This systematic review highlights several gaps in licit and illicit substance use (SU) and substance use disorder (SUD) literature within Kenya, with the goal of summarizing research within three broad domains: (1) epidemiologic studies, (2) intervention and/or programs and (3) qualitative studies. The authors apply sound methods, with attention to details around decision-making processes when including articles in their review. The attention to target study populations (e.g., community, hospitals, prisons, etc.) is extremely valuable and calls for additional studies within specific populations. In addition, the authors make the case that their review is needed in order to address Kenya's Vision 2030 and moves towards accomplishing SDG's. I commend the authors for completing this large undertaking and offer feedback to strengthen and improve their paper.

Major Edits

- There is an absolute need for SU and SUD systematic review; however, this paper may have limited applications in its current state. In the introduction, the authors state this paper will "guide future research efforts"; however, most SUD researchers work with one substance or one category of substances. It would be helpful within the key findings sections to expand on SU categories, which are discussed briefly in the introduction (e.g., tobacco, alcohol, opioids, cannabis, and stimulants.) Another option may be to reformat the paragraphs according to SU categories and discuss the current epidemiologic, interventions/programs, and qualitative studies.
- In your criteria, you do not mention whether you included studies conducted out of methadone clinics or harm reduction sites (i.e., drop-in centres, NSPs), specifically. However, when I look over the publications, several were conducted within these sites. Please clarify whether these terms were part of your search categories and include them on Page 11, lines 215-217.
- Throughout the descriptions and key findings sections, there should be more syntheses of the data instead of frequencies, which are already conveyed in your tables. For example, under the epidemiology section of SU/SUD, you say that 47% of the studies used evidence-based diagnostic tools, but this should be followed by the key findings of those studies (i.e., X-X% of participants indicated hazardous or harmful alcohol consumption, and X-X% of participants indicated alcohol dependence.) This is just one example, but all of the key finding's sections should provide more data syntheses.
- As it stands, the key findings and other findings sections are a little difficult to follow and are heavily focused on alcohol and tobacco use. For example, in the epidemiologic key findings section the paragraphs are organized as follows: (1) youth and substance use, (2) adults and tobacco use, (3) adults and alcohol use, and (4) two case control studies. Again, this may have a better flow if the authors organized the key findings by SU categories (e.g., tobacco, alcohol, opioids, cannabis, and stimulants.) By structuring the paragraphs by SU categories, the reader is able to quickly decipher where there are gaps in the literature. Alternatively, the authors may

want to consider narrowing the scope of their paper by solely focusing on alcohol and tobacco use, which seem to be the main focus throughout the paper.

- In the qualitative study key findings section, most of the studies apply frameworks and/or theories to their analysis (e.g., stages of change, risk environment framework), which should be synthesized and included as a column in Additional File 5/Qualitative Studies.

<u>Minor Edits</u>

- Please review the PLOS ONE Guidelines on formatting references and edit references.
- Page 11 (line 220) "People with injecting drug use" should be "people (or persons) who inject drugs."
- Page 11 (line 221) "Men who have Sex with Men" should not contain capital letters.
- Page 11 (lines 218-225) This section does not sum up to the total studies in the epidemiology section n=144.
- Page 11 (line 210-213) Please be consistent in how you mention the study designs with corresponding references. This was completed in the interventions and programs section, but not for the epidemiological studies.
- Page 15 (lines 299-303) Conversely, please indicate in the programs and intervention section, how may studies were included in each of the study designs.
- Page 12 (line 229) typo, please change to "opioids (n=21)"
- In the findings section, please define "hospital," and whether this includes methadone clinics.
- Page 20 (line 398) "Substance use" should be "substance use disorder."
- Page 21 (line 423-424) "Mental disorders" should be "mental health disorders."
- Additional File 3/Epidemiological Studies: The SU category should not include how people consume their drugs ("injection drugs"), which is only seen a few times, but what drugs categories were examined. Please be more specific than "illicit drugs."
- Additional File 4/Interventions and Program: Please review the sample sizes for each study, particularly for those with "not reported."
